# Epidemics, Lockdown Measures and Vulnerable Populations: A Mixed-Methods Systematic Review of the Evidence of Impacts on Mother and Child Health in Low-and Lower-Middle-Income Countries

**DOI:** 10.34172/ijhpm.2021.155

**Published:** 2021-11-07

**Authors:** Giuliano Russo, Tiago Silva Jesus, Kevin Deane, Abdinasir Yusuf Osman, David McCoy

**Affiliations:** ^1^Wolfson Institute of Population Health, Queen Mary University of London, London, UK.; ^2^Global Health & Tropical Medicine, Instituto de Higiene e Medicina Tropical, Nova University of Lisbon, Lisbon, Portugal.; ^3^Faculty of Arts & Social Sciences, School of Social Sciences & Global Studies, Open University, Milton Keynes, UK.; ^4^Royal Veterinary College, University of London, London, UK.

**Keywords:** Lockdown Measures, Epidemics in LMICs, Mother and Child Health, COVID-19, Public Health and Quarantine, Non-Pharmaceutical Interventions

## Abstract

**Background:** The aim of this research was to synthetise the existing evidence on the impact of epidemic-related lockdown measures on women and children’s health in low- and lower-middle-income countries (LLMICs).

**Methods:** A mixed-methods systematic review was conducted of qualitative, quantitative and mixed-methods evidence. Between 1st and 10th of November 2021, seven scientific databases were searched. The inclusion criteria were that the paper provided evidence on the impact of lockdown and related measures, focused on LLMICs, addressed impacts on women and child’s health, addressed epidemics from 2000-2020, was peer-reviewed, provided original evidence, and was published in English. The Joanne Briggs Institute’s critical appraisal tools were used to assess the quality of the studies, and the Preferred Reporting Items for Systematic Reviews and Meta-Analyses (PRISMA) guidelines for reporting. The evidence from the papers was grouped by type of lockdown measure and categories of impact, using a narrative data-based convergent synthesis design.

**Results: **The review process identified 46 papers meeting the inclusion criteria from 17 countries that focussed on the coronavirus disease 2019 (COVID-19) and Ebola epidemics. The evidence on the decrease of utilisation of health services showed plummeting immunisation rates and faltering use of maternal and perinatal services, which was linked to a growth of premature deaths. Impacts on the mental health of children and women were convincingly established, with lockdowns associated with surges in depression, anxiety and low life satisfaction. Vulnerability may be compounded by lockdowns, as livelihoods were disrupted, and poverty levels increased.

**Conclusion:** Limitations included that searches were conducted in late-2020 as new research was being published, and that some evidence not published in English may have been excluded. Epidemic-related lockdown measures carry consequences for the health of women and children in lower-income settings. Governments will need to weigh the trade-offs of introducing such measures and consider policies to mitigate their impacts on the most vulnerable.

## Introduction

 Prohibition of social gathering, trade and travelling restrictions, border closures, quarantines and curfews are some of the public health measures that have been used historically to contain the spread of infectious diseases. *Cordons sanitaire* – the deployment of physical barriers around towns and villages to restrict entrance – were implemented to stop the spread of bubonic plague, yellow fever and cholera in Europe since the 17th century.^1^ The word quarantine comes from the 40-day isolation periods that were imposed on traders in Venice travelling from regions affected by the plague in the 14th century. More recently, such measures have been described as non-pharmaceutical interventions (NPIs) and have been adopted as part of the response to epidemics of severe acute respiratory syndrome (SARS) (2003), H1N1 influenza (2009), Ebola (2014) and, currently in the containment of the coronavirus disease 2019 (COVID-19) pandemic.^2,3^ The current pandemic has also seen the widespread adoption of the term ‘lockdown’ to describe those NPIs that involve mandatory restrictions on normal social and economic life that are applied indiscriminately to whole populations.^4^

 Although such measures are effective at containing the spread of infectious diseases, they may also be harmful.^5^ They can cause significant disruption to social and economic life, impinge upon civil liberties and personal freedoms, and produce illness and direct harm. With COVID-19, where strict disease control measures have been implemented for prolonged periods of time, many concerns have been raised about the collateral damage or undesirable effects of these measures, notwithstanding their importance in containing the spread of the disease. Concerns include the social and economic harms of these measures.^6,7^ Evidence of the unintended or harmful health effects of epidemic control measures include reports of increasing food insecurity, particularly for families with children in low-income settings, deteriorating mental health, poorer eating habits and a lowering of physical activity, as well as reducing access to and utilisation of healthcare services.^8-11^ Disruption of livelihoods and of social determinants of health is also believed to carry a longer-term impact on population health.^12^

 It has also been noted that such adverse effects are unevenly experienced between and within populations.^13^ On the one hand, low- and lower-middle-income countries (LLMICs) are widely believed will fare worse than high-income countries (HICs), given the more limited fiscal room for governments to provide welfare and economic support for the millions already on the breadline, and with housing conditions of millions of indigent households making some lockdown measures particularly challenging.^14^ On the other hand, poorer and marginalised population sub-groups are likely to be more exposed and vulnerable to the harms of these measures, such as informal workers, ethnic minorities, and people with no qualifications or low literacy.^15^ The well-being of women and children is also feared to be particularly at risk during the current COVID-19 epidemic, because of the suspension of essential services, school closures and potential of domestic violence from the imposed lockdowns.^16^

 Although the current data suggest that many LLMICs may have been less directly affected by the virus, with lower rates of severe infections, possibly in connection to the different demographics and the lesser capacity of local systems to report cases, they are more vulnerable to the harms of lockdown measures, defined as those mandatory or voluntary measures taken to control travel and the environment to contain contagion during epidemics.^17-19^ Either way, governments in LLMICs have a particular need to carefully assess and monitor the balance between the health protection provided by COVID-19 control measures, and the harms and collateral damage of those same measures, particularly as it affects vulnerable populations, including women and children. This is additionally important given that vaccine roll-out across LLMICs is likely to be delayed.

 We set out to synthetise the quantitative and qualitative evidence on the health impacts on children and women in LLMICs of so-called lockdown measures associated with the major acute epidemics of the last 20 years. This review aims to contribute to current debates about the best way to minimise the harms associated with communicable disease control measures, with a particular focus on the needs of vulnerable populations in low-income settings.

## Methods

###  Approach and Design of the Review

 We conducted a mixed-methods systematic review synthesizing diverse types of knowledge (such as qualitative, quantitative and mixed-method evidence) with configurative (that is, not aggregative) purposes.^20-23^ For reporting, we used the guidelines of the Preferred Reporting Items for Systematic Reviews and Meta-Analyses (PRISMA) statement,^24^ and prospectively submitted the systematic review protocol for registration on PROSPERO (CRD42020220324).

 We drew from existing conceptualisations of the direct and indirect effects of lockdown measures used to control the transmission of epidemics to frame the review design as well as to identify the search terms for the health repercussions on women and children in low-income settings.^25,26^ We also used a recently published framework for identifying the equity harms of epidemic-related interventions for vulnerable populations in low-income countries.^13^ A preliminary search of public health literature also helped in identifying both the short- and long-term impacts of such policies and informed the development of the search strategy and eligibility criteria.

 Although the literature does not always specify which of the different types of lockdown measure carry a health impact, we drew from our previous work on this subject and focused on synthesising the effects of stay-at-home measures, restrictions on movement and trade, prohibition of mass gatherings, suspension of health and education services on the health of women and children.^27^

###  Search Strategy

 Seven scientific databases (PubMed; ISI Web of Science – Core Collection; Scopus; Cochrane Library; SciELO, Econ Lit, and PDQ-Evidence) were searched for, one more (ie, the PDQ-Evidence) than originally planned in our PROSPERO protocol. Altogether, these databases cover the mainstream health, public health, health systems, multidisciplinary, and economic literature. The full search strategies employed for each database are provided in Supplementary file 1. Database searches were conducted between 1st and 10th of November 2020, covering studies published no earlier than the year 2000 and with title and/or abstract available in English.

 Besides database searches, snowballing searches (that is, citations tracking; authors tracking; references list consultation) were conducted over the articles selected through the database searches. Secondary searches also entailed searching websites and/or databases (for example, using key search terms) of the following outlets: the World Bank’s; the Organization for Economic Co-operation and Development’s; Health Policy and Planning’s resources for epidemics.

###  Selection Criteria

 We included primary research studies published in peer-reviewed journals, using quantitative, qualitative or mixed-methods, existing systematic reviews of primary research, original analyses of secondary data, including modelling studies, country-specific or cross-country reports, and cost-effectiveness studies. We excluded: unpublished study reports, papers published in non-peer-reviewed journals, commentaries and opinion pieces, and non-systematic reviews. The population of interest was children (<18) and women in LLMICs as per the World Bank’s 2021 income classification.^28^

 We included impacts and exposure to lockdown measures taken to prevent or contain epidemic or pandemic events between 2000 and 2020 such as COVID-19, Ebola, SARS, Middle East respiratory syndrome (MERS), Zika, Swine flu, and Avian flu. We only included evidence related to the list of 29 low-income and 50 lower-middle-income countries as defined by the World Bank in 2021.^28^ Drawing from the classification from a recent Cochrane Review on the subject,^19^ the following measures were considered: (*a*) Geographic containment policies for example, border closures, international travelling bans; in-country travelling restrictions; (*b*) Closures and prohibitions (such as closures of schools; prohibitions of gatherings; closures of non-essential shops and businesses; restrictions of gatherings and opening hours in markets and worship venues); (*c*) Home confinement measures (such as: advise to stay at home; curfews); (*d*) Suspension of services (such as: immunisation campaigns; restrictions in accessing health facilities; restriction in accessing welfare services; suspension in the provision of healthcare services like check-up, screening, treatments and preventive services) (Table 1).

**Table 1:** Inclusion and Exclusion Criteria for the Selection of the Identified Records

**Inclusion**		**Exclusion **	
**Criteria**	**Example**	**Criteria**	**Example**
Impacts of epidemic-related lockdown measures, NPI	Impact of closures, bans and curfews	Not a lockdown measure of interest – eg, Direct effects of epidemic(s) or pharmaceutical interventions	Impact of COVID-19 on children’s health; impact of hydroxychloroquine on COVID-19
Effects of lockdown measures on health and social determinants of health	Mental health impact of lockdown measures; impact on education, poverty, employment or inequalities	Impacts of lockdown measure(s) not on non-health-related domains	Impact of COVID-19 on research, theatres or performing arts
Explicit evidence of effects on children or women	Effects of suspension of immunisation campaigns on children	Not specific to children and women - General evidence of impact on population or professionals	Effects on population health of suspension of PHC services; effects in health workers
LLMICs	29 Low-income countries from World Bank list; 50 lower-middle income countries from the World Bank 2021 list	Not from LLMICs. From upper-middle income and HICs	
Lockdown measures applied during epidemics in the last 20 years	Isolation measures taken during Ebola and MERS outbreaks	Not from epidemics in last 20 years - Measures taken during non-epidemic outbreaks	Impact of curfews during conflicts
Research pieces	Original research; Analysis	Not a research article	Commentaries, editorials, letters, non-systematic reviews, reflections, perspectives, viewpoints
Peer-reviewed evidence	Papers published in peer-reviewed, indexed journals	Not from peer-reviewed journal	Unpublished reports, blogs, newspapers pieces
Primary quantitative, qualitative and mixed-methods evidence of effects	Surveys, secondary analysis of large datasets, interviews, focus group, modelling, case-studies, systematic reviews	Not producing original scientific evidence	Quoting secondary data, non-systematic reviews
Articles in English	Articles with at least titles and abstract in the English language	Articles not in English	Articles with only a title in English, but abstract and body text in a foreign language

Abbreviations: NPIs, Non-Pharmaceutical interventions; COVID-19, coronavirus disease 2019; HICs, high-income countries; MERS, Middle East respiratory syndrome; LLMICs, low- and lower-middle-income countries; PHC, primary healthcare.

 The effects and outcomes of interest included those related to mental health, nutrition; physical activity, chronic, maternal or neonatal conditions and illnesses, interpersonal violence, children’s learning and development, poverty and social vulnerability. These criteria were applied both to the Level 1 (titles-and-abstract) and Level 2 (full-text) screenings.

###  Data Collection and Risk of Bias Assessment 

 The database searches were conducted by TSJ. The initial Level 1 screening was conducted by GR to identify sources that may be of relevance to our study objectives. Level 2 screening was conducted by KD and AYO, with each reviewer independently looking at the abstract and full text to assess whether the source met the inclusion criteria. The Level 2 reviewers returned three possible decisions: ‘included,’ ‘excluded’ or for ‘Uncertain: seek further information.’ For each source that was excluded, the reviewers recorded a reason from related to the inclusion and exclusion criteria. For each source that was judged as ‘seek further information,’ a third reviewer (GR) made an independent assessment regarding whether the sources should be included.

 Where studies met the eligibility criteria, their methods’ quality was also appraised using the Joanne Briggs Institute’s critical appraisal tools covering 12 different types of study designs.^29^ The reviewers who performed the Level 2 screenings also performed this assessment, using one checklist per paper, appropriate for the study design. This process was instrumental to exclude any papers with important methodological shortcomings from the synthesis, as recommended by the reviewers after completing the appraisal checklists, and further appraised by GR, extensive to the whole team as necessary. All the methodological shortcomings identified through this process were presented in either the results or limitations sections.

###  Data Extraction and Analysis

 Every paper passing the Level 2 screening and quality appraisal was subject to data extraction. Data extraction was split between GR, KD and AO. The research team developed an original data extraction table and coding structure addressing the paper’s geographic and demographic coverage, as well as their methodological features (eg, study design, type of evidence, sampling, outcomes assessed, analytical methods, variables controlled for). Key findings on the outcome measures, in turn, were extracted for free text boxes. Descriptive statistics (eg, counts, rates) were applied to describe the outcomes of the papers included. In turn, the findings on the impact of the lockdown measures were tabulated per type of measure, per population covered (ie, children and women), before being narratively summarized. The bibliographic references were managed in Endnote and Zotero software; characteristics of each studies and respective key findings were organised in an Excel database and analysed through pivot dynamic tables.

 In the analytical process within each category of impact, we applied a ‘data-based convergent synthesis design,’ with all types of data (ie, quantitative and qualitative) synthesized under the same method, this time narratively.^20,22,23^

## Results

Figure 1 presents the PRISMA flowchart of the review. From 880 records initially detected (751 after duplicates were removed), 63 full texts were deemed eligible, of which 46 papers were finally included. Supplementary file 2 provides a list of the papers finally included. Supplementary file 3 provides the finalised PRISMA checklist. Supplementary file 4 provides the list of papers excluded after full-text review, along with the reasons for their exclusion. Finally, Supplementary file 5 provides the data extraction forms and quality appraisals.

**Figure:**
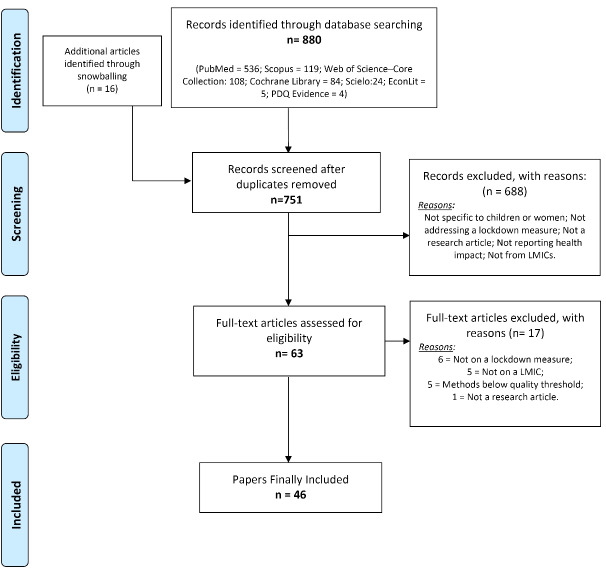
PRISMA Flowchart for the Systematic Review. Abbreviations: PRISMA, Preferred Reporting Items for Systematic Reviews and Meta-Analyses; LMIC, low- and middle-income country.

###  Scope of the Evidence Included

 Forty-six research papers, all published between 2015 and 2020, met the inclusion criteria. Of these, 31 focused on the recent COVID-19 pandemic, and 15 on the 2014-2015 Ebola epidemic in West Africa (Table 2). The papers covered 17 countries across 3 continents. There were no relevant papers covering the SARS, Zika, MERS, Swine flu or Avian flu epidemics. Of the papers, 16 (34.8%) were based on primary surveys, 11 reported on modelling exercises, 6 were based on an analysis of routinely collected surveillance and healthcare utilisation data, 4 were based on qualitative research, 4 used mixed-methods, 2 reported on a time-series analysis and there was one economic evaluation.

**Table 2:** Papers Included in the Review, by Epidemic Considered and Type of Evidence

**Type of Evidence**	**COVID-19**	**Ebola**	**Total**
Analysis of routinely collected surveillance and utilisation data	2	4	6
Economic evaluation	0	1	1
Mixed methods	2	2	4
Modelling	10	2	12
Qualitative evidence from interviews	2	2	4
Survey data	14	2	16
Time-series analysis	1	2	2
**Grand total**	**31**	**15**	**46**

Abbreviation: COVID-19, coronavirus disease 2019.

Table 3 summarises the evidence and shows that the disruption in the provision of routine services was the most common health impact identified in 14 of our papers, leading in four cases to the identification of potential deaths.^30-33^ Lockdown’s impact on the utilisation of healthcare services was the subject of eight of the papers, particularly for those referring to the Ebola epidemic.^34-36^ Evidence of mental health impacts from lockdown measures was also discussed in eight papers,^37,38^ followed by social vulnerability effects including increase in poverty levels, reduced income or livelihood was covered in six papers.^39,40^ The rest of the papers focussed on altered dietary patterns, nutrition and lack of physical exercise,^41^ domestic violence,^42^ and disrupted sleeping patterns among children and adults.^43^

**Table 3:** Papers Identified for the Review, by Lockdown Measures and Health Impacts

**Health Impact/Main Lockdown Measure Reported**	**Restrictions on Trade**	**School Closures**	**Social Distancing**	**Stay at Home Restrictions**	**Suspension of Services**	**Unspecified Quarantine**	**Total Papers**
Deaths			Bell et al^33^		Weiss et al,^30^ Jewell et al,^32^ Hogan et al^55^		4
Reduced utilisation of healthcare services	Awucha et al^51^			Gichuna et al^56^	Ly et al,^34^ McQuilkin et al,^35^ Quaglio et al,^36^ Kolie et al ^57^	Hung et al,^58^ Carias et al^59^	8
Dietary patterns, nutrition and physical exercise				Ammar et al^49^		Kodish et al,^41^ Kodish et al^44^	3
Disruption of routine mother and child services					Siekmans et al,^60^ Plucinski et al,^46^ Parpia et al,^47^ Chandir et al,^59^ Sun et al,^62^ Salama et al,^63^ Takahashi et al,^64^ Abbas et al,^65^ Wagenaar et al^48^	Saleem et al,^66^ Chelo et al,^67^ Roberton et al,^45^ Delamou et al,^68^ Kc et al,^69^	14
Domestic violence				Hamadani et al,^70^ Sediri et al ^42^			2
Mental health		Yeasmin et al,^53^ Sama et al^54^		Sharma et al,^71^ Ammar et al,^72^ Ali et al^37^		Darvishi et al,^73^ Bhumika,^74^ Pandey et al^38^	8
Sleeping patterns				Dutta et al^43^			1
Social vulnerability	Aura et al,^50^ Ceballos et al^52^					Mottaleb et al,^75^ Gutiérrez-Romero et al,^39^ Mohapatra,^40^ Mathew et al76	6
**Total papers**	3	2	1	7	16	16	46

 Unfortunately, 16 of the papers did not specify the specific lockdown measure causing health impacts, referring only to the application of ‘generic lockdown’ or ‘quarantine measures’ (see for example,^38,44,45^). Others (16) referred specifically to the suspension of healthcare services implemented to contain the spread of epidemics.^46-48^ Seven studies addressed the effects of ‘Stay at home’ policies (for example,^37,49^), three the restrictions on trade and business,^50-52^ two mentioned school closures,^53,54^ and one social distancing.^33^

 We present in the following sections the extracted evidence below, from the categories of impact for which richer material was found, to the ones subject of comparatively fewer papers (reduced utilisation of healthcare; lockdown-related loss of lives; impact on mental health, and; impact on social vulnerability). Within each category we describe and analyse the link between impacts and specific lockdown measures, type of evidence, and geographical focus of the studies. We also provide a full summary of the evidence from each paper in Table 4.

**Table 4:** Summaries of Evidence for All Included Papers

**First Author and Date**	**Main Findings**	**Sample Population Characteristics/Data Sources**
**Deaths**		
Bell et al,^33^ 2020	Decline of 75% in reporting of new AIDS cases and initiation of ART. The authors predicted an overall loss of 475 319 DALYs from disruption of therapy and detection of new cases. Different scenarios of mortality (from 3000 to 31 000), and DALY lost to malaria (most of them for children), ranging from 257 000 to 2 450 000. Maternal mortality: A 29% (28 939) reduction in facility deliveries is recorded in the Ministry of Health Uganda data in March compared with January 2020, 28% less than the 12-month average for 2019. Over the same period, an 82% increase in maternal mortality was recorded (from 92 to 167 women), an increase of 87% over the 12-month 2019. An excess 486 deaths are predicted for a 6-month period, incurring 31 343 DALYs lost.	Secondary data from a range of sources including Uganda Bureau of Statistics census report PEPFAR weekly surge reports Uganda population-based HIV impact assessment final report Uganda HMIS quarterly reporting.
Weiss et al,^30^ 2020	Under 9 different scenarios of disruption of services, there could be additional 215-262 million cases of malaria worldwide, and between 101-382 thousand extra malaria deaths. Malaria control relies heavily on the decision making of patients and their families, including choosing to leave their homes to seek care for febrile children and receiving ITNs delivered at antenatal clinics or schools. A substantial proportion of the additional cases and deaths would be from children <5 years.	Secondary data from a wide range of sources, including some data which are modelled resulted from previous analyses. Data is from the Malaria Atlas Project, Global Burden of Disease Study and the World Malaria Report.
Jewell et al,^32^ 2020	According to different scenarios of disruption of services (from 20% to 100% disruption), the models predict between 92 000 and 956 000 excess deaths in sub-Saharan Africa in one year. Interruption of ART would increase mother-to-child transmission of HIV by approximately 1.6 times, with a similar increase of mortality for newborns.	Study used five existing HIV models to estimate impact of disruption of HIV services: Goals, Optima HIV, HIV Synthesis, Imperial College London Model, EMOD.
Hogan et al,^55^ 2020	Under different scenarios of COVID-19 mitigation and suppression, it was calculated there would be up to 596 extra deaths per million people due to HIV/AIDS, 987 per million people due to TB, and up to 1018 per million people due to malaria.	Modelling uses different models/data sources for each health condition. HIV model draws on data from South Africa and Malawi, Malaria model draws on data from Malaria Atlas Project, TB model uses WHO 2018 estimates.
**Reduced Utilisation of Healthcare Services**		
Emmanuel Awucha et al,^51^ 2020	35.2% of the respondents managing chronic illnesses had difficulties accessing essential medicines during the COVID-19 lockdown, with 84.0% experiencing deteriorating chronic health conditions in the light of difficulty in accessing their medicines. Increase in the cost of medicines was observed by 77.7% of participants, with 73.9% of respondents living with chronic illness affirming that their income was negatively affected by the pandemic.	Cross-sectional survey of 374 participants, sampled online and through social media. 58% aged between 16-30. 46% of the sample population were female. Health workers were excluded from the study. Data not disaggregated by sex.
Gichuna et al,^56^ 2020	Unable to access healthcare due to movement restrictions and also having to conduct more business during the day (when clinics were open), Reduction of peer support, Reduced access to Challenges accessing ARV treatment and PrEP – potentially increased HIV risk, Reduced access to family planning services – noted that there would be ‘corona babies’ and unwanted pregnancies, Reduced supply of condoms.	Qualitative interviews with 117 female sex workers from informal settlements in Nairobi East. Age ranges: 16-24 (32.4%), 25-33 (50.4%), 34-42 (6.8%), 43+ (1.7%). 15 healthcare providers also interviewed.
Ly et al,^34^ 2016	The authors detected a 30% decreased odds of facility-based-delivery after the start of Ebola epidemic in a rural Liberian county with relatively few cases. The odds of facility-based delivery were 41% lower among women who reported a belief that Ebola was or may be transmitted in health facilities, but not significantly lower among women who reported believing that Ebola was not transmitted in health facilities.	Household survey of 1298 women in Rivercess county. 941 households participated. All women agreed 18-49 were surveyed. Median age of participants was 29 years, 86.3% had less than secondary education.
McQuilkin et al,^35^ 2017	More than half (67%) of urban respondents and 46% of rural respondents stated that it was very difficult or impossible to access healthcare during the epidemic. For those who sought care at government hospitals and were unable to receive it, the major barriers were closure of facilities (50%), healthcare workers refusing to see patients (42%), and fear of referral to Ebola treatment units (2%).	Survey with participants sampled from 5 communities that were located within a one-hour drive of the 21 government hospitals in Liberia. Total number of participants was 548 (282 rural, 266 urban), 50.2% women, 45% of respondents had secondary school education or higher education.
Quaglio et al,^36^ 2019	The study found decrease in all MCH indicators and service uptake immediately after the onset of the outbreak, with a levelling or increase during the Ebola period. In the post-Ebola period, all indicators (except for maternal deaths) showed an increase in utilisation of health services compared to pre-Ebola period. The study highlights that increase in service utilizations particularly in hospital settings was due to the post-Ebola reinforcement of the RS with special reference to paediatric admissions, maternal admissions, and consequently a rise of institutional deliveries, C-sections and major direct obstetric complications.	Prospective data gathered from routinely collected health services data in Pujehun district, including hospital registers, hospital data bases and the district health management information system. Three different time periods (pre-Ebola, Ebola, post-Ebola) were defined and compared.
Kolie et al,^57^ 2018	In the Ebola-affected district of GueÂckeÂdou, there was a 30% decrease in total clinical visits, malaria testing for >5 children. During the peak of the Ebola outbreak, there was a significant decrease in oral antimalarial drug administration, which corresponded to an increase in injectable antimalarial treatments. Stock-outs in rapid diagnostic tests were evident and prolonged in GueÂckeÂdou during the outbreak, while more limited in Koubia.	Retrospective cross-sectional study. Data used was routine malaria surveillance data reported to the Guinean National Malaria Control Programme. Data covered all under five children who presented at 19 health centres.
Hung et al,^58^ 2020	Total visits and visits for pneumonia and diarrhoea initially increased more than two-fold relative to the control areas, while institutional deliveries and first antenatal care increased between 20% and 50%. Visits for DTP, fourth antenatal care visits and postnatal care visits were not significantly affected.	Retrospective, controlled ITS study. Data was from the HMIS, an electronic database derived from facility-level data. 10 zones included in the analysis.
Carias et al,^59^ 2016	Administration of preventive ACT to contacts of patients with Ebola virus disease was cost saving for contacts of all ages, as it avoided hospitalization or being mistakenly admitted to Ebola treatment units. The intervention was calculated to be cost saving in contacts in areas with malaria parasite prevalence in children aged 2–10 years as low as 10%.	Economic evaluation using a decision tree model. Data used from a wide range sources including the Malaria Atlas Project, population statistics for the different African countries reported by the US Census Bureau’s International Database and previous academic studies.
**Dietary Patterns, Nutrition and Physical Exercise**		
Ammar et al,^49^ 2020	The COVID-19 home confinement had a negative effect on all PA intensity levels (vigorous, moderate, walking and overall). The number of days/week and minutes/day of vigorous intensity PA during, compared to before, home confinement decreased by 22.7% Additionally, daily sitting time increased from 5 to 8 hours per day. Food consumption and meal patterns (the type of food, eating out of control, snacks between meals, number of main meals) were more unhealthy during confinement, with only alcohol binge drinking decreasing significantly.	Online multi-country survey. This article uses first 1047 responses. 53.8% of sample were women. Regional distribution: 40% North Africa, 36% western Asia, 21% Europe, 3% other. 55% of participants aged 18-35, 35.1% aged 36-55, 9.9% above 55. Education levels 88.2% had a bachelors degree or above.
Kodish et al,^41^ 2019	Negative impact on food security and nutrition due to reduction in production (people could not go to their farms or the market). Shops were closed. Most mothers and family heads were not able to work. For those that could afford it, they were unable to buy baby food at the shops. Altered infants and young children feeding practices. Reduced screening for malnutrition cases.	42 interviews: 21 with key informants (33% women) such as Government, UN, NGOs and hospital management 21 with Community informants (85.7% women) including household members, community leaders and CHWs.
Kodish et al,^44^ 2019	Stakeholders agreed that infant and young child nutrition was adversely impacted by: (a) Poor access to the health system (b) Household food (c) insecurity (d) Changing breastfeeding practices.	Qualitative approach involved participatory workshops with 17 and 19 participants in Guinea and Sierra Leone, respectively. Stakeholder interviews were conducted with representatives from a range of stakeholders including from government, UN bodies, civil society, non-governmental organizations and local communities 27 interviews were conducted in Guinea, 42 in Sierra Leone.
**Disruption of Routine Mother And Child Services**		
Siekmans et al,^60^ 2017	73% of CHWs reported decrease in cases that they consulted. Evidence from health facilities that service provision decreased at the peal of the crisis. Medicine stocks were available during the outbreak but CHWs reported inability to access them due to travel restrictions and facility closures.	Mixed methods study which included a survey and focus group discussions with CHWs, government facility workers and project staff from 3 different counties in Liberia. Routine monitoring data also used.
Plucinski et al,^46^ 2015	The survey found: 11% reduction in all-cause outpatient visits 15% reduction in cases of fever 24% reduction in patients treated with oral antimalarial drugs 30% reduction in patients treated with injectable antimalarial drugs Antenatal visits by pregnant women also significantly reduced Malaria management by CHWs in affected areas decreased.	Cross sectional survey of 120 public health facilities in eight prefectures. This included health facilities from the four most impacted prefectures plus an additional 4 randomly sampled. 15 facilities in each prefecture were randomly selected.
Parpia et al,^47^ 2016	An estimated 50% reduction in access to healthcare services during the Ebola outbreak exacerbated malaria, HIV/AIDS, and tuberculosis mortality rates by additional death counts of 6269 (2564–12 407) in Guinea; 1535 (522–28 780) in Liberia; and 2819 (844–4844) in Sierra Leone. Mortality attributable to malaria increased by 48% in Guinea, 53.6% in Liberia and 50% in Sierra Leone. 50% reduction in ART coverage increased HIV-related deaths by 16.2%, 13% and 9.1% respectively. Increase in TB deaths due to reduced treatment coverage estimated to be 51.1%, 59% and 61.4%.	Three computational simulation models: disease progression model for malaria and 2 decision tree models for HIV/AIDS and active TB cases. Models drew on data from Global Burden of Disease studies and estimates from the published literature.
Chandir et al,^61^ 2020	There was a 52.5% decline in the daily average total number of vaccinations administered during lockdown compared to baseline. The highest decline was seen for BCG (40.6%; 958/2360) immunization at fixed sites. Around 8438 children/day were missing immunization during the lockdown. Enrolments declined furthest in rural districts, urban sub-districts with large slums, and polio-endemic super high-risk sub-districts. Daily average vaccinator attendance was 7.4% lower during the lockdown compared to baseline (78.8% [79 252/100 600 person days] vs. 86.2% [312 386/362 551 person days] respectively).	Analysis of immunisation records from Government of Sindh’s Zindagi Mehfooz (Safe Life) Electronic Immunization Registry. The registry covers all 29 districts of Sindh. 276 districts included in the study due to lack of baseline data for 2 districts.
Sun et al,^62^ 2017	Measles vaccine coverage among age-eligible children was 71.3% before the Ebola outbreak and 45.7 during the outbreak. Pentavalent vaccine (Pentavalent3) coverage among age-eligible children was 79.8% before the outbreak and 40% during the outbreak of Ebola.	Survey covered parents of 168 children aged under 4 born between May 2011 and April 2015 (94 boys, 74 girls). Children sampled from a total of 3 villages in 3 of the communities covered by the China Public Health Training Team. Villages selected randomly (total of 35 villages across the 3 communities.
Salama et al,^63^ 2020	There was a substantial reduction of the availability score for Available fertility preservation options for girls with cancer in India. Significant reduction of availability of cancer treatment for boys in India for testicular cancer treatment, as well as availability of chemio and radio therapy. There was a substantial reduction of the availability score for available fertility preservation options for girls with ovarian and breast cancer in India and Nigeria.	Survey of oncofertility centres in 14 developing countries. Centres were part of existing network OPEN.
Takahashi et al,^64^ 2015	Assuming a 75% reduction of vaccination rates, the study projects that after 6 to 18 months of disruptions, a large cluster of children unvaccinated for measles will accumulate across Guinea, Liberia, and Sierra Leone. This pool of susceptibility increases the expected size of a regional measles outbreak from 127 000 to 227 000 cases after 18 months, resulting in 2000 to 16 000 additional deaths from multiple infectious diseases in the community. With every month of healthcare disruptions, the study estimated that the number of children between 9 months and 5 years of age who are not vaccinated against measles increases by an average of 19 514, reaching 1 129 376 after 18 months. In the likely case of outbreaks, this susceptibility could generate up to 5209 additional deaths from measles only.	Estimation of vaccine coverage and modelled projections of impact of disruptions. Data drawn from Demographic and Health Surveys in Guinea, Liberia, Sierra Leone, and surrounding countries.
Abbas et al,^65^ 2020	The benefit of routine childhood immunisation programmes in all 54 African countries was found to be greater than the COVID-19 risk associated with these vaccination clinic visits. For every one excess COVID-19 death attributable to SARS-CoV-2 infections acquired during routine vaccination clinic visits, 84 deaths in children up to 5 years of age could be prevented by sustaining routine childhood immunisation in Africa.	Benefit risk model. Parameters drawn from the existing literature on health impacts of vaccine disruption, and country specific population estimates of the vaccinated cohort, country-specific and antigen-specific official country reported estimates of vaccination coverage.
Wagenaar et al,^48^ 2018	The authors found that it took only 4 months during the Ebola epidemic to lose between 35% and 67% of essential primary care health system outputs across Liberian clinics, and that 19 months post-Ebola, all health system indicators had recovered to their pre-Ebola levels. They estimated a loss of an estimated 776 110 clinic visits; 101 857 artemisinin-based combination therapy treatments for malaria, and 45 024 treatments of acute respiratory infections due to the EVD outbreak will continue to severely affect population health. They estimated a loss of 24 449 bacille Calmette-GueÂrin vaccinations, 9129 measles vaccinations, 12 941 first pentavalent vaccinations, 5122 institutional births, 17 191 postnatal care visits within 6 weeks of birth.	Time-series analysis using data from the Liberian Ministry of Health RHIS which covers all health facilities nationwide. Health facilities in Montserrado county were excluded.
Saleem et al,^66^ 2020	38% of the patients lost their job during the crisis. 64% had an appointment cancelled due to COVID-19 pandemic. 17.4% had medication discontinued due to disruption 26.8% reported worsening of seizures. Reported reliance on free antiepileptic drug supplies from hospital: Totally 30.5%, partially 57.3%	Cross sectional study of caregivers of paediatric patients with active epilepsy who had been recruited for a previous study. Sample size was 213 caregivers, 60.1% female.
Chelo et al,^67^ 2020	27% and 47% drop in hospitalizations during the months of April and May 2020 respectively as compared to the same period in 2019. Mortality doubled during the months of April and May 2020 with 9.9% and 11.2% respectively of hospital deaths compared to 4.9% and 5.1% during the same period of the previous year.	Retrospective cross-sectional survey drawing from data from all children attending the Mother and Child Center of the Chantal Biya Foundation in Yaounde. Average annual attendance was 34 600 children.
Roberton et al,^45^ 2020	Scenario 1 (smallest reductions coverage of essential maternal and child interventions) resulted in an additional 42 240 child deaths per month, and scenario 3 (greatest reductions) resulting in an additional and 192 830 child deaths per month. The additional child deaths would represent relative increases of 9·8% (scenario 1), 17·3% (scenario 2), and 44·7% (scenario 3) in child deaths per month. Main causes are an increase in wasting prevalence, reduced coverage of antibiotics for pneumonia and neonatal sepsis and of oral rehydration solution for diarrhoea.	Lives Saved Tool used to estimate impact of reduced coverage of essential maternal and child interventions. 118 LMICs included.
Delamou et al,^68^ 2017	Most maternal and child health indicators significantly declined during the Ebola virus disease outbreak in 2014. Despite a reduction in this negative trend in the post-outbreak period, the use of essential maternal and child health services has not recovered to their pre-outbreak levels. Fewer institutional deliveries occurred and fewer women achieved at least one antenatal care visit after the outbreak. The greatest reductions between the pre and during phases were noted for polio and tuberculosis at -3594 and -3048 fewer vaccines administered, respectively.	A retrospective, observational cohort study of women and children attending public health facilities for antenatal care, institutional delivery, and immunisation services in six of seven health districts in the Forest region. Data collected from all health facilities in these districts. Time series analysis of 3 periods (pre, during and post epidemic).
Kc et al,^69^ 2020	The mean weekly number of hospital births decreased from 1261.1 births before lockdown to 651.4 births during lockdown—a reduction of 52.4%. The institutional stillbirth rate increased from 14 per 1000 total births before lockdown to 21 per 1000 total births during lockdown and institutional neonatal mortality increased from 13 per 1000 livebirths to 40 per 1000 livebirths. The average weekly reduction in institutional births during lockdown was 7.4%, resulting in the overall reduction of 52.4% by the end of lockdown.	Prospective observational study. 9 health institutions in Nepal that were enrolled previously in two improvement programmes. Institutions located across all 7 provinces. 21 763 women enrolled in the study.
**Domestic Violence**		
Hamadani et al,^70^ 2020	A reduction in work for the father or other family members was reported by 2321 (96.0%) of the families in the sample. Median monthly income fell from US$212 at baseline to $59 during lockdown. At baseline, 5 (0.2%) of 2422 families were earning less than $1.90 per day, and during the lockdown this number increased to 992 (47.3%,) of 2096 comparing baseline with lockdown period. Maternal mental health deteriorated during the lockdown. Symptoms of depression increased among women during lockdown. 68.4% of women who reported emotional violence at baseline (19.9%) reported an increase during lockdown. 56% of women who reported. experiencing physical violence at baseline (6.5%) reported an increase during lockdown. 50.8% of women who reported experiencing sexual violence at baseline (3%) reported an increase during lockdown.	Time series analysis using data collected from mothers (or female guardians) of children enrolled in the BRISC trial in Rupganj upazila (county) of Narayanganj district. Participants randomly selected. Sample size was 2424. 97.3% of mothers were unemployed at baseline.
Sediri et al,^42^ 2020	More than half of the survey participants (57.3%) reported extremely severe distress symptoms. Violence against women y increased significantly during the lockdown (from 4.4 to 14.8%; *P* < .001). Psychological abuse was the most frequent type of violence (96%). Women who had experienced abuse before the lockdown were at an increased risk of violence during lockdown (OR = 19.34).	Online survey using snowball sampling from an initial sample of 5 participants in Tunisia. Final sample consisted of 751 women. Median age was 37 years. 66.6% had high school or university education. 69% were married.
**Mental Health**		
Yeasmin et al,^53^ 2020	43% of child had subthreshold mental health disturbances (mean depression: 2.8, anxiety: 2, and sleeping disorder: 1), 30.5% had mild disturbances (mean depression: 8.9, anxiety: 4.9, and sleeping disorder: 3), 19.3% suffered from moderate disturbances (mean depression:15.9, anxiety: 9.2, and sleeping: 6), and 7.2% suffered from severe disturbances (mean depression: 25.2, anxiety: 13.4, and sleeping disorder: 8).	Online survey conducted with parents of children aged between 5 and 15 years. Total sample of 384. Potential participants contacted via social media. 63.3% urban, 36.7% rural. 46.6% aged between 36–45 years.
Sama et al,^54^ 2020	73.2% and 51.6% of the children displayed signs of increased irritation and anger, respectively; 18.7% and 17.6% of the parents also mentioned the symptoms of depression and anxiety, respectively, among their children, which were also augmented by the changes in their diet, sleep, weight and more usage of electronic equipment.	Telephone survey completed with 310 parents from four districts of Punjab, India, (Ludhiana, Sahibzada Ajit Singh Nagar, Sangrur and Ferozepur). Two districts had the highest and two the lowest number of COVID-19 cases.
Sharma et al,^71^ 2020	General Anxiety Disorder Scale scores higher for: LBGT^a^ adults vs. heterosexual adults (β = 2.44), High risk groups vs. low risk groups (β = 2.20); History of depression vs. no history of depression (β = 3.89).	Online survey, snowball sampling and distribution via social media. 282 responses from Indian citizens aged 18 and over. 75% of sample were aged 30 or lower. Women represented 36% of the LBGT survey sample. 15 qualitative interviews also conducted.
Ammar et al,^49^ 2020	Participants reported lower life satisfaction because of home confinement. Statistical analysis showed the total score of SSPQL (decreased significantly by 42% “during” compared to “before” home confinement (t = 69.19, *P* < .001, d = 2.14).	Online multi-country survey. This article uses first 1,047 responses. 53.8% of sample were women. Regional distribution: 40% North Africa, 36% western Asia, 21% Europe, 3% other. 55% of participants aged 18-35, 35.1% aged 36-55, 9.9% above 55. Education levels 88.2% had a bachelors degree or above.
Ali et al,^37^ 2020	The overall mean score for well-being was 42.4, indicating that 51.9% of adults suffered from poor mental health. The participants who were involved in business had worse mental health than government employees healthcare workers and employees of private companies. 57% of women were in poor mental health (ie, WEMWBS score ≤42), whereas for men it was at 48.9%. Unmarried women report higher well-being scores than the married women.	Online survey promoted via social media. 1523 responses received, 1404 participants included in final analysis. 63.2% male, 36.8% female. 54.6% aged between 20-29. 83.5% had an undergraduate or postgraduate degree.
Darvishi et al,^73^ 2020	67.3% of participants may have demonstrated OCD symptomatology. The prevalence of obsessive-compulsive disorder symptoms in female students was slightly higher than in male students (72.1% compared to 60.3%).	Survey of 150 high school students aged 13-19 years. 67.4% were women. Average age was 16.37 for women and 16.97 for men.
Bhumika,^74^ 2020	Women reported more emotional exhaustion than men due to personal life interference in work during work from home period. Work Interference with Personal Life was found to be positively related to emotional exhaustion. Personal life interfering with work) was found to be positively related to emotional exhaustion.	Online survey with 180 working professionals in North India contacted through professional network. 51.7% of respondents were male. 78.3% worked in the private sector. 64.4% were agreed between 25 and 35 years.
Pandey et al,^38^ 2020	The reported prevalence of depression was 30.5%. Anxiety was reported by 22.4%, followed by stress which was seen in 10.8% of respondents. In the third week the incidence of depression (37.8% versus 23.4%), anxiety (26.6% versus 18.2%) and stress (12.2% versus 9.3%) was reported to be significantly higher as compared to second week. Women were more susceptible to suffer from all forms of psychological symptoms (depression, anxiety and stress) in comparison to men. A statistically significantly higher proportion of women had mild to severe level of depression anxiety and stress in comparison to men.	Online survey with a snowball sampling strategy, distributed via social media. 1395 responses were received. 82.8% were aged between 18-30 years, with 50.4% in the age bracket of 18–20 years. 82.7% were unmarried and 76% were students. 58.1% were women.
**Sleeping Patterns**		
Dutta et al,^43^ 2020	While 42.9% of respondents experienced deeper sleep during lockdown, 31.4% experienced more discontinuous sleep during the lockdown phase. About 37.1% reported more daytime sleepiness during the lockdown phase. Higher frequencies of naps were reported, with 25.7% of participants taking daily naps. Overall, screen exposure did not change significantly in a high percentage of subjects before and during lockdown – with the exception of the comparison between week-days usage.	Online survey distributed via social media and email. Final sample included 153 children aged 8-16.
**Impact on Social Vulnerability**		
Aura et al,^50^ 2020	Restrictions (Cessation of movement to cities that are the main fish markets, curfews and social distancing) impacted fishing trips and duration: Fishing time was reduced (76%, n = 116) Fishing trips per week reduced from an average of seven to five trips (n = 103) 79% of respondents said that COVID-19 measures impacted the fishing industry ‘very much’ (as opposed to ‘much’ or ‘a little’). Fish traders and processors reported being affected the most due to closures of markets. There was a notable decline in the average crew (fishing inputs) and boat fuel (consumable) used in fishing activities resulting into a cross-cutting decline in catch quantities and prices.	Study sites were Kenyan major lakes (Victoria, Turkana, Baringo and Naivasha). Study involved a socio-economics survey on perceptions and attitudes of purposively selected categories of stakeholders at the lake landing sites. 336 respondents. 80% fishers and fish-traders, 52% of the sample were men.
Ceballos et al,^52^ 2020	Because of the shortages of seasonal labourers linked to travelling restrictions, 41%-80% of farmers responded that they had to spend more on labour to harvest. Because of the closures of the local markets, 61%-74% responded they had to store their harvest and sell in the future. No difference in access to food was reported before and after the lockdowns.	Phone survey conducted in Haryana and Odisha. Farmers were enrolled in an existing study. Sample came from 100 villages in 4 districts in Haryana, and 50 villages in Jajpur district in Odisha. Final sample was 1515 farmers.
Abdul Mottaleb et al,^75^ 2020	Under the assumption of a complete lockdown with no-one allowed to work, the economic loss in one day is estimated at BDT 5389.03 million or approximately US$ 64.2 million. Assuming 50% of the daily wage workers are not allowed to work, the economic loss/day will be BDT 2694.5 million or US$ 32.1 million. It is estimated that on average it is necessary to provide BDT 51-104 or around US$ 1 per day to wage-based households during lockdown to ensure minimum food security.	Logistic regression using data from Bangladesh’s 2016–2017 HIES. Final sample was 50 671. 34 301 (67.7%) were paid other than daily basis mode, 7552 (14.9%) worked in the farm sector and were paid daily, and 8818 (17.4%) worked in the nonfarm sector and were paid daily. Average of 36 years old, with nearly five years of schooling. More than 80% were married, and more than 67% were from rural areas.
Gutiérrez-Romero and Ahamed,^39^ 2020	Results forecast that globally, the percentage of people living under $1.90 a day would increase from 13.1% in 2019, to about 13.8% in 2020 and 14.5% by 2021. This represents an increase of 107.8 million people in poverty, using the $1.90 dollars a day poverty line. The percentage of people worldwide living under $3.20 a day would increase from 24.8% in 2019 to nearly 27% by 2021, pushing nearly 169.4 million people in poverty.	Modelling exercise. Study uses three key data sources: Financial Access Survey database, IMF and World Bank economic growth data, and PovcalNet.
Mohapatra,^40^ 2020	An economic growth shock creates a sharp decline in female employment by 3 percentage points within the first 5 years after the shock. The magnitudes of the employment reductions are large. For instance, applied to the Indian context, the total number of female workers according to the 2011 census is about 150 million. A 3 percentage point drop implies that, following a GDP contraction, there are 4.5 million jobs lost 5 years after the shock over the previous year.	Study uses panel data from the World Development Indicators database. Data used is from 1991-2019 for two regions, South and South East Asia and West Africa.
Mathew et al,^76^ 2020	100% of the research participants reported inadequate food supplies as a result of closing down of their self-run businesses. 100% of the research participants reported hopelessness to revive their business due to spending their savings during lockdown. They also reported a loss of all their perishable goods at the start of the lockdown. 100% of the research participants reported poor access to reproductive health services due to poor transport network during lockdown. They also reported poor access to maternal health services and all participants reported feelings of anxiety and depression.	Qualitative approach involving in-depth interviews with 40 participants. Sampling approach was through faith groups and local community organisations. Interviews conducted online. 60% of sample were aged 21-40, 40% married, 40% single, 20% widowed.

Abbreviations: PEPFAR, President’s Emergency Plan for Aids Relief; ART, anti-retroviral therapy; COVID-19, coronavirus disease 2019; DALY, disability-adjusted life year; HMIS, Health Management Information System; TB, Tuberculosis; WHO, World Health Organization; ITS, interrupted time-series; UN, United Nations; NGOs, non-governmental Organisations; CHWs, community health workers; BCG, Bacille Calmette Guérin; OPEN, Oncofertility Professional Engagement Network; SARS-CoV-2, Severe acute respiratory syndrome coronavirus 2; LMICs, low- and middle-income countries; BRISC, Benefits and risks of iron interventions in children; OR, odds ratio; SSPQL, Short Social Participation Questionnaire-Lockdowns, WEMWBS, Warwick Edinburgh Mental Well-being Scale; BDT, Bangladeshi Taka; HIES, Household Income and Expenditure Survey; GDP, gross domestic product; IMF, International Monetary Fund; ITNs, insecticide-treated nets; EMOD, Epidemiological Modeling Software; ARV, antiretroviral; PrEP, pre-exposure profilaxis; MCH, mother and child health; RS, re-organised referral system; DTP, diphtheria, pertussis and tetanus; ACT, artemisinin-based combination treatment; EVD, Ebola virus disease; RHIS, routine health information system; OCD, Obsessive compulsory disorder; PA, physical activity. ^a^LGBT stands for lesbian, gay, bisexual, and transgender.

###  Impact on Utilisation of Mother and Child Services

 Six studies reported on the effects of mother and child services being disrupted in the course of both the Ebola and COVID-19 epidemics. Takahashi and colleagues’ modelling study on reduced measles vaccination rates in Guinea, Liberia, and Sierra Leone due to the Ebola epidemic projected that concluded that there would be up to 1 129 376 children unvaccinated for measles after 18 months of the start from the epidemic.^64^

 Delamou and colleagues’ retrospective observational study in Guinea found that most maternal and child health service indicators significantly worsened during the 2014 Ebola virus disease epidemic in 2014.^68^ The most significant reductions were noted for polio and tuberculosis vaccinations at –3594 (*P* < .001) and –3048 (*P* = .04) administered, respectively. Fewer institutional deliveries, and fewer pregnant women attended at least one antenatal care visit or at least three antenatal care visits per month (*P* < .001 for all).

 A prospective observational study by Kc et al on the effects of the COVID-19 epidemic in Nepal found that during lockdown the institutional neonatal mortality rate increased from 13 per 1000 livebirths to 40 per 1000 livebirths (*P* = .002); the average weekly reduction in institutional births during lockdown was 7.4%, with a total decrease of 52.4% by the end of lockdown.^69^ In terms of quality of care, intrapartum foetal heart rate monitoring decreased by 13·4% and breastfeeding by 3·5%. The proportion of women who had a complication during childbirth increased from 6.7% before lockdown to 8.7% during lockdown (*P* = .01). The proportion of women who had caesarean section increased from 24.5% before lockdown to 26.2% during lockdown (*P* = .007).

 Facing the prospects of disruption of vaccination services during lockdowns, Abbas and colleagues’ risk-benefit analysis derived from their modelling suggested that the benefit of continuing with routine childhood immunisation programmes in all 54 African countries would outweigh the risk of COVID-19 infection associated with vaccination clinic visits.^65^ They found that for every excess COVID-19 death attributable to infections acquired during routine vaccination clinic visits, there would be an estimated 84 deaths in children up to 5 years of age due to reduced childhood immunisation.

 There is also evidence that fear of acquiring infection from health facilities can reduce utilisation, and Ly et al detected a 30% decreased odds of institutional birth deliveries after the start of Ebola epidemic in a rural Liberian county despite the relatively few cases.^34^ The odds of facility-based delivery were 41% lower among women who reported a belief that Ebola was or may be transmitted in health facilities, but not significantly lower among women who reported believing that Ebola was not transmitted in health facilities.

 Finally, Quaglio and colleagues’ analysis of routine surveillance and utilisation data in a rural district in Sierra Leone showed statistically significant differences in trends between pre-Ebola versus post-Ebola for paediatric admissions, maternal admissions, major direct obstetric complications, and institutional deliveries.^36^

###  Loss of Lives Due to Lockdown Measures

 Our review also revealed evidence that lockdown measures result in excess mortality. A modelling study on the potential effects of disruption to HIV programmes caused by COVID-19 found that, according to different scenarios (ranging from 20% to 100% disruption levels), there could be between 92 000 and 956 000 excess deaths in sub-Saharan Africa in one year, with a substantial proportion of expecting mothers.^32^ Interruption of anti-retroviral treatment would increase mother-to-child transmission of HIV by approximately 1.6 times, with a similar increase of mortality for new-borns.

 Another modelling study on the effects of COVID-19-related lockdown measures in Uganda predicted a decline of 75% in the reporting of new AIDS cases and initiation of anti-retroviral therapy (ART), resulting in a loss of 475 319 disability-adjusted life years (DALYs) due to disruption of treatment and detection of new cases; and different scenarios of mortality (from 3000 to 31 000), and DALYs lost to malaria (most of them for children), ranging from 257 000 to 2 450 000.^33^ The same study reported a 29% (28 939) reduction in monthly facility deliveries recorded by the Ministry of Health compared with January 2020, a reduction of 28% with respect to the 12-month average for 2019. Over the same period, an 82% increase in maternal deaths was recorded (from 92 to 167 women, in absolute terms), an increase of 87% over the 12-month 2019 average of 89.5. An excess 486 deaths are predicted for a 6-month period, incurring a loss of 31 343 DALYs.

###  Impact on Mental Health

 The impact of lockdowns measures on mental health was also an important topic highlighted by our review. A survey of 1404 adults in Bangladesh using the Warwick Edinburgh Mental Well-being Scale (WEMWBS) found that 51.9% of adults suffered from poor mental health during the first 4 months of the COVID-19 epidemic.^37,70^ Participants involved in business had poorer mental health compared to government employees (decreased by 5.9 units, *P* = .01), healthcare workers (by 5, *P* ≤ .001), and employees of private companies (by 3.3, *P* = .02). Depression seemed to disproportionally affect women - 57.2% of female participants resulted in poor mental health (WEMWBS score ≤42), whereas for males it was at 48.9% (*P* = .002). Furthermore, it was found that unmarried women recorded higher well-being scores than the married women (by 3.31, *P* < .001).

 Similar results were reported from ‘The COVID-19 Lockdown (COLD) Study’ in India, according to which the prevalence of depression among adults was 30.5%, anxiety was reported by 22.4% participants, followed by stress, seen in 10.8% of respondents. In the third week of lockdown the prevalence of depression (37.8% versus 23.4%; *P* < .001), anxiety (26.6% versus 18.2%; *P* < .001) and stress (12.2% versus 9.3%; *P* = .045) was reported to be significantly higher in comparison to the second week.^38^ In this study women were also more likely to suffer from all forms of psychological symptoms (depression, anxiety and stress); a significantly higher proportion of women reported higher levels of mild to severe depression (*P* = .002), anxiety (*P* = .002) and stress (*P* < .001) as compared to the men.

 A worldwide multicentre study also found that COVID-19-related home confinement negatively impacts social participation and life satisfaction as measured by the SSPQL (Short Social Participation Questionnaire-Lockdowns), a short modified questionnaire to assess social participation before and during a lockdown period on the basis of reported participation in 18 types of social activities.^72^ Among the study participants, the total score of SSPQL score decreased significantly by 42% “during” confinement compared to “before” home confinement (t = 69.2, *P* < .001, d = 2.1). Women comprised 53.8% of the overall study sample.

 Another online cross-sectional study conducted between April and May 2020 in Bangladesh showed that 43% of children had subthreshold mental health disturbances (mean depression: 2.8, anxiety: 2, and sleeping disorder: 1); 30.5% had mild disturbances (mean depression score of 8.9, anxiety: 4.9, and sleeping disorder: 3); 19.3% suffered from moderate disturbances (mean depression:15.9, anxiety: 9.2, and sleeping: 6), and; 7.2% suffered from severe disturbances (mean depression score of: 25.2, anxiety: 13.4, and sleeping disorder: 8).^53^

###  Changes in Dietary Patterns, Sleeping and Domestic Violence

 The evidence on how lockdown measures affect people’s health-related behaviour is more heterogeneous.

 A qualitative study in Sierra Leone highlighted the main pathways by which lockdown measures implemented during the Ebola epidemic impacted on child nutrition, including through reduced availability of milk formula in shops, altered infant and young child feeding practices, reduced monitoring of growth and detection of malnutrition, and restrictions in the ability of people to forage for food.^41^ The study also described how these effects of lockdown were mediated by other factors including mistrust between the government and communities. Similar findings about the impact of lockdown measures on child nutrition were found in a consensus building exercise on feeding practices during Ebola with stakeholders in Sierra Leone and Guinea by the same authors.^44^

 Preliminary results from a worldwide online survey on eating and physical activity during the COVID-19 pandemic showed that home confinement measures reduced physical activity intensity levels, and increased daily sitting time from 5 to 8 hours per day (*P* > .001).^49^ Food consumption patterns, including the type of food consumed, the frequency of binge eating and snacking between meals, and the number of main meals, were found to be less healthy during confinement. The frequency of binge drinking, however, was reported to have decreased significantly.

 An online survey on the impact of COVID-19 home confinement measures on children’s sleeping patterns and screen time in India revealed that while 42.9% of respondents experienced deeper sleep during lockdown, 31.4% experienced more discontinuous sleep during the lockdown phase.^43^ Higher frequencies of napping during the day were also recorded among participants (25.7%). However, screen exposure did not change significantly among children before and during lockdown – with the exception of the comparison between week-day usage.

 Finally, from the domestic violence perspective, an interrupted time series analysis study among households participating in a Randomised Controlled trial in Bangladesh found that symptoms of depression increased among women during lockdown (10, 3–17; 6-point increase, in the interquartile range 0–11 scale); *P* < .001; emotional violence increased with respect to baseline, including insults (initially reported by 19.9% of participants) for which 68.4% reported an increase, humiliation (66.0% of 191 reported an increase), and intimidation (68.7%) of 291 reported an increase.^70^ Physical violence (eg, being slapped or having something thrown at them) was initially reported by 6.5%, and 56% reported an increase during lockdown. Sexual violence was less common (3.0%), but of those affected, 33 (50.8%) reported an increase since the lockdown. Along the same lines, another piece of research among Tunisian women found that during the 2020 COVID-19 confinement more than half of the participants (57.3%) reported extremely severe distress symptoms, as per the Depression Anxiety and Stress Scales assessment tool; violence against women also was reported to have increased significantly during the lockdown (from 4.4% to 14.8%; *P* < .001).^42^ Psychological abuse was the most frequent type of violence (96%). Women who had experienced abuse before the lockdown were at an increased risk of violence during lockdown (odds ratio = 19.3 [8.7-43.0]).

###  Impact on Social Vulnerability

 Six papers that presented evidence on the impact of COVID-19-related lockdown measures on livelihoods and poverty levels (Table 3). A World Development paper used a modelling methodology to forecast that the percentage of people living under $1.90 a day would increase worldwide from 13.1% in 2019, to about 13.8% in 2020 and 14.5% by 2021 as a result of the policies adopted to contain the COVID-19 epidemic.^39^ A survey of smallholder farmers in Haryana and Odisha states designed to assess the impacts of India’s national lockdown on farmers’ income and food security found that, because of the shortages of seasonal labourers linked to travel restrictions, 41%-80% of farmers faced steeper labour costs to harvest. Because of the closures of the local markets, 61%-74% responded having had to store their harvest and sell in the future.^52^ No difference in access to food was reported before and after the lockdowns.

 From their interviews of 40 self-employed women in Zambia, Mathew et al relate that 100% of the research participants experienced inadequate food supplies as a result of closing down of their self-run businesses. All the research participants also reported a sense of hopelessness about being able to revive their business due to having spent their savings during the lockdown or because of a loss of all their perishable goods at the start of the lockdown.^76^

 In the same vein, Aura and colleagues’ survey of the Great Lakes’ fishing communities in Kenya assessed the consequences of COVID-19 pandemic on fish capture and trade.^50^ They concluded that the cessation of movement to cities where the main fish markets are located, curfews and social distancing, affected fishing trips and duration, disrupted the fish value chain, diminished trade, and negatively affected the livelihoods of fishermen and fisherwomen.

 A modelling study on food security for vulnerable groups in Bangladesh estimated that under the assumption that 50% of the country’s daily wage workers were not allowed to work during lockdown, a one-day complete lockdown would generate $64.2 million worth of losses to the country’s economy.^75^ The study also estimated that income support of around US$1 per day per household would be needed to ensure basic food security for daily wage-based worker households.

 Another study drawing from participants in a randomised controlled trial of iron supplementation in children (the BRISC trial – Benefits and risks of iron interventions in children) in Bangladesh found a reduction in work for the father or other family members in 96.0% (n = 2321) of the families in the sample. Median monthly income fell from US$212 at baseline to $59 during lockdown.^70^ While only 0.2% (5) of families lived below the income poverty line of $1.90 per day at baseline, this figure increased to 47.3% (*P* < .001) during the lockdown.

## Discussion

 This review found substantial and diverse evidence of the health impacts of lockdown measures on women and children from two distinct epidemics, reflecting a wide range of different impacts and related mechanisms. The data on the decrease of utilisation of health services seemed convincing, with studies from the COVID-19 and Ebola lockdowns showing plummeting immunisation rates and faltering use of maternal and perinatal services. Modelling work has linked such effects to an increase in the number of premature deaths in LLMICs. The negative impact of lockdown measures on the mental health of children and women also seems well-established, with home confinement measures associated with rises in levels of depression and anxiety, and a lowering of feelings of life satisfaction.

 We also found evidence of socio-economic vulnerability increasing with lockdown measures as livelihoods were disrupted and poverty levels increased amid trade restrictions and bans, particularly for women-led households. Some evidence was also uncovered of rises in domestic violence during home confinement, although pre-existing violent relations appear to play a role in the occurrence of new cases.

 This review also identified a general paucity of research on the impact of lockdown measures, as we only found evidence related to the Ebola and COVID-19 epidemics. This may be because the terms ‘lockdown’ and ‘non-pharmaceutical intervention’ are relatively recent, and because research in this area has traditionally been dominated by clinical and epidemiology health specialists focussing on the direct impacts of epidemics.^77^ It is however noteworthy that evidence was found from these two epidemics on the health impacts on children and women from the associated lockdown restrictions. We did not find much on the effects of school closures or other social determinants on the health of women and children either, even though also non-health specific databases were also searched. This may be in part explained by the complexity of disentangling these complex relations and associations, but also by the timing of our search, as it will take time for such macro effects to produce an impact on the health of vulnerable populations.^78^

 The evidence uncovered was very heterogeneous and of diverse quality, and with a notable exception, a lack of large-scale, representative studies was noticeable.^69^ This is in part due to restrictions on collection of primary data, and a reliance on online surveys that do not provide representative samples in LLMICs contexts. As measures to contain the COVID-19 epidemic take their toll, it is likely that new studies on their impact will be carried out, and an attempt should be made to monitor and document the production of new and stronger evidence beyond the one reviewed here. In the absence of alternatives, modelling methods have taken prominence in the analysis of the projected effects of the current epidemic, and it is out for debate whether there is enough good quality empirical data to feed into the models to produce accurate predictions.

 Another methodological challenge of this review was that a detailed description of the ‘lockdown measures’ being implemented was not always clearly provided by the studies retrieved. As such, it was not always possible to determine which communicable disease control measures were impacting on women and children, and through which pathways. Often the ‘lockdown measures’ were vaguely defined or used as a collective term for a broad range of restrictions designed to contain epidemic.^27^ Future research needs to include clearer definitions of which aspects of lockdown are under investigation.

 This issue also limited the understanding of the mechanisms through which lockdown measures impact on health. For example, how the suspension of healthcare services during an epidemic impacts on healthcare utilization was not always fully explored in the studies we reviewed. For example, while a supply-side effect on utilisation is to be expected when providers suspend their services, some evidence from the Ebola epidemic in West Africa,^34,48^ and COVID-19 in Nepal,^69^ suggests that the suspension of services carries a longer-term effect on the demand-side too, as patients will keep avoiding health providers for fear of contagion even when services resume. Whether these are supply or demand-side effects matters, as the policy interventions needed to counterbalance low utilisation would differ accordingly.^79^

 Our review finds emerging evidence that lockdown measures have a disproportionate impact on vulnerable groups such as women and children in LLMICs. Evidence from the lockdown measures in West Africa suggests some pattern of impacts, as interruption of services would be responsible for the largest share of health effects on vulnerable groups.^34,48,64^ This would be consistent with evidence that poorer individuals have lower levels of healthcare utilisation compared to other population groups, and are among the first to stop seeking care when barriers are created to their access.^80^ Suspending basic primary healthcare services during an epidemic would therefore have a disproportionate, long-term effect on women and children, particularly when cost-effective immunization programmes and institutional childbirth strategies are considered.^69,81^ As the consequences of lockdowns appear to be different for HICs and upper middle-income countries, this highlights the importance of monitoring the evidence in low-income settings, and consider that even in HICs, socioeconomic differences can amplify the negative impact of restrictive lockdown measures on the most sensitive sectors.^82^

 Our review emphasises that preliminary evidence points to very divergent impacts for different social groups with respect to changes on food and nutrition, with wealthier households reporting over-eating, snacking and binge drinking, an experience that is in contrast to poorer households that face increased food insecurity.^44,83^ Until effective treatments or vaccines are made widely available in LLMICs, lockdown restrictions are likely to remain in place at least during the peak of epidemics.^84^ It will be essential to gauge their impacts on specific vulnerable groups with more precision to understand when and how to intervene to avoid long-lasting damage.

 Ultimately, our analysis of the available evidence shows that the consequences of epidemic-related lockdown measures can be greater than expected for voiceless, vulnerable groups in LLMICs. Measures successfully applied in some high-income contexts may not necessarily work in LLMICs, and it will be critical for local governments to constantly monitor the balance of the trade-offs between the imperative of containing epidemics, and the inevitable damage that these policies leave behind. However, the range of both predictable and unintended consequences of lockdowns on women’s and child health presents a significant challenge to policy makers. The evidence is clear that lockdown policies need to be accompanied with other measures designed to mitigate these impacts; prioritisation of competing health instances will be reliant on future high-quality research to inform responses to potential future waves of COVID-19 or new infectious disease outbreaks.

 The following limitations apply to this review. The searches were conducted in late 2020, as COVID-19 publications continue to be published and fresh evidence is produced at an unprecedented rate, faster than our ability to process the information from continuous updates. Therefore, there is clearly an opportunity for continuous review updates for this area of lockdown measure impacts, such as those allowed by living systematic review methodologies^85^ used in other COVID-19 topics.^86^ Our searches only covered articles with titles and/or abstracts in the English language, which may have reduced access to evidence from low-income countries. Our initial database searches did not include pre-print servers such as medRxiv. Although at least two reviewers were always involved in each stage of the review, the second reviewer was mostly used in a verification role, rather than as an independent reviewer, to accelerate the review process. However, no crucial step was missed in conducting the review (eg, skipping quality assessments, focus in one sole or a few databases, neglecting snowballing searches), meaning that our review process was consistent with that of a fully-fledged mixed-methods systematic review.^20^ Although we did conduct quality assessments of the included papers for exclusion purposes and for highlighting existing methodological weaknesses, we did not use this procedure to rank the existing evidence according to its quality, as per the definitions of the Joanna Briggs Institute checklists.^29^ Such checklists were considered adequate as our review had a large and exploratory scope, a configurative purpose, and involved a narrative, nuanced synthesis rather than meta-analyses.

## Conclusion

 Lockdown measures have been put in places for centuries to contain epidemics worldwide, but their consequences are largely unknown, particularly for the health of vulnerable populations. We looked at the evidence on impacts of lockdown measures on the health of women and children in LLMICs, to provide an evidence base for governments to weight costs and benefits of such measures. Seven scientific databases were searched for research papers from the 2000-2020 period reporting on lockdowns’ impacts on mental health, nutrition, utilisation of services, exposure to interpersonal violence, increased poverty and social vulnerability. The Joanne Briggs Institute’s critical appraisal tools and the PRISMA guidelines were used for assessing the quality of the studies and for reporting.

 We identified 46 research papers meeting the inclusion criteria, all focussing on the two most recent COVID-19 and Ebola epidemics. The evidence on the decrease of utilisation of health services showed plummeting immunisation rates and faltering use of maternal and perinatal services, which was linked to a growth of premature deaths. Lockdowns’ impact on mental health of children and women appeared to be well-established, with stay-at-home measures associated with surges in depression, anxiety and low life satisfaction. The evidence suggests that vulnerability may be compounded by lockdowns, as livelihoods are disrupted, and poverty levels increase. We concluded that epidemic-related lockdown measures do carry unintended consequences for the health of women and children in LLMIC settings; governments will need to ponder the trade-offs of such measures and consider policies to mitigate the impacts for the most vulnerable.

## Ethical issues

 Not applicable.

## Competing interests

 Authors declare that they have no competing interests.

## Authors’ contributions

 GR contributed to designing the review, extracting the evidence, and drafted the manuscript. TSJ contributed to designing the review and revised the manuscript. KD contributed to designing the review, extracting the evidence, and revised the manuscript. AYO contributed to extracting the evidence and reviewing the manuscript. DMC contributed to designing the review and reviewing the manuscript.

## Supplementary files


Supplementary file 1. Search Strategy.

Supplementary file 2. List of Papers Included in the Review.

Supplementary file 3. PRISMA Checklist.

Supplementary file 4. List of Papers Excluded, With Reason for Exclusion.

Supplementary file 5. Data Extraction and Quality Appraisal Form.
