# Supplementary material for: Epidemics, Lockdown Measures and Vulnerable Populations: A Mixed-Methods Systematic Review of the Evidence of Impacts on Mother and Child Health in Low-and Lower-Middle-Income Countries
Source: Int J Health Policy Manag. 2021 Nov 7;11(10):2003–21. doi: 10.34172/ijhpm.2021.155 (PMC9808285; doi:10.34172/ijhpm.2021.155)
Supplement: Supplementary file 1 — Search Strategy. [file ijhpm-11-2003-s001.pdf]

Article title: Epidemics, Lockdown Measures and Vulnerable Populations: A Mixed-Methods Systematic Review of the Evidence of Impacts on Mother and Child Health in Lowand Lower-Middle-Income Countries

Journal name: International Journal of Health Policy and Management (IJHPM)

Authors' information: Giuliano Russo[1]*, Tiago Silva Jesus[2], Kevin Deane[3], Abdinasir Yusuf Osman[4], David McCoy[1]

[1]Wolfson Institute of Population Health, Queen Mary University of London, London, UK.

[2]Global Health & Tropical Medicine, Instituto de Higiene e Medicina Tropical, Nova University of Lisbon, Lisbon, Portugal.

[3]Faculty of Arts & Social Sciences, School of Social Sciences & Global Studies, Open University, Milton Keynes, UK.

[4]Royal Veterinary College, University of London, London, UK

(*Corresponding author: g.russo@qmul.ac.uk)

Supplementary file 1. Search Strategy

PubMed 16/11/2020 **536**

("Epidemics"[Major] OR "Pandemics"[Major] OR "COVID-19" [Supplementary Concept] OR "severe acute respiratory syndrome coronavirus 2" [Supplementary Concept] OR "COVID-19" OR "SARS-COV-2" OR "SARS Virus"[Mesh] OR "Middle East Respiratory Syndrome Coronavirus"[Mesh] OR "Hemorrhagic Fever, Ebola"[Mesh] OR "Ebolavirus"[Mesh]) **AND** (Lockdown* or closure* or shutdown* or "stay at home" or curfew* or confinement* or restriction* or ban or prohibition or suppression* or disrupt* or constraint* or (consequence* or impact* not "Therapeutics"[Mesh] not "Chemicals and Drugs Category"[Mesh] not "Diagnosis"[Mesh] not "Surgical Procedures, Operative"[Mesh]) or "Quarantine"[Mesh] or "Poverty"[Mesh] or "Social Determinants of Health"[Mesh] or "Health Policy"[Mesh] or "Economics"[Mesh]) **AND** ("Study Characteristics" [Publication Type] OR "Empirical Research"[MeSH] OR "Epidemiologic Methods"[MeSH] OR (Review[ptyp] AND systematic[tw] AND systematic[sb]) OR "Cochrane Database Syst Rev"[Journal] OR ("systematic review"[ti] OR "scoping review"[ti] OR "realist review"[ti]) OR "Support of Research" [Publication Type]) **AND** ("2000/01/01"[PDAT]: "2020/12/31"[PDAT]) **AND** ("Developing Countries"[Mesh] OR "Africa South of the Sahara"[Mesh] OR "Argentina"[Mesh] OR "Africa"[Mesh] OR ("China"[Mesh] NOT "Hong Kong"[Mesh] NOT "Macau"[Mesh]) OR "Democratic People's Republic of Korea"[Mesh] OR "Mongolia"[Mesh] OR "Asia, Central"[Mesh] OR "Asia, Northern"[Mesh] OR "Bangladesh"[Mesh] OR "Bhutan"[Mesh] OR "India"[Mesh] OR "Nepal"[Mesh] OR "Pakistan"[Mesh] OR "Sri Lanka"[Mesh] OR "Afghanistan"[Mesh] OR "Iran"[Mesh] OR "Iraq"[Mesh] OR "Jordan"[Mesh] OR "Lebanon"[Mesh] OR "Syria"[Mesh] OR "Turkey"[Mesh] OR "Yemen"[Mesh] OR "Cambodia"[Mesh] OR "Indonesia"[Mesh] OR "Laos"[Mesh] OR "Malaysia"[Mesh] OR "Myanmar"[Mesh] OR "Philippines"[Mesh] OR "Thailand"[Mesh] OR "Timor-Leste"[Mesh] OR "Vietnam"[Mesh] OR "Mexico"[Mesh] OR "Belize"[Mesh] OR "Costa Rica"[Mesh] OR "El Salvador"[Mesh] OR "Guatemala"[Mesh] OR "Honduras"[Mesh] OR

"Nicaragua"[Mesh] OR "Cuba"[Mesh] OR "Dominica"[Mesh] OR "Dominican Republic"[Mesh] OR "Grenada"[Mesh] OR "Haiti"[Mesh] OR "Jamaica"[Mesh] OR "Saint Lucia"[Mesh] OR "Saint Vincent and the Grenadines"[Mesh] OR "Bolivia"[Mesh] OR "Brazil"[Mesh] OR "Colombia"[Mesh] OR "Ecuador"[Mesh] OR "Paraguay"[Mesh] OR "Peru"[Mesh] OR "Suriname"[Mesh] OR "Venezuela"[Mesh] OR "Albania"[Mesh] OR "Bosnia and Herzegovina"[Mesh] OR "Bulgaria"[Mesh] OR "Kosovo"[Mesh] OR "Republic of North Macedonia"[Mesh] OR "Montenegro"[Mesh] OR "Moldova"[Mesh] OR "Republic of Belarus"[Mesh] OR "Serbia"[Mesh] OR "Ukraine"[Mesh] OR "Transcaucasia"[Mesh] OR "Kazakhstan"[Mesh] OR "Kyrgyzstan"[Mesh] OR "Uzbekistan"[Mesh] OR "Samoa"[Mesh] OR ("Micronesia"[Mesh] NOT "Guam"[Mesh] NOT "Palau"[Mesh]) OR "Fiji"[Mesh] OR "Papua New Guinea"[Mesh] OR "Vanuatu"[Mesh] OR "Tonga"[Mesh]) **AND** ("Infant"[Mesh] OR "Child"[Mesh] OR "Adolescent"[Mesh] OR "Women"[Mesh] OR "Women's Health Services"[Mesh] OR "Pregnant Women"[Mesh] OR "Women, Working"[Mesh] OR "Women's Rights"[Mesh] OR "Battered Women"[Mesh] OR "Female"[Mesh] OR "Disabled Children"[Mesh] OR "Aid to Families with Dependent Children"[Mesh] OR "Homeless Youth"[Mesh] OR "Child, Adopted"[Mesh] OR "Child of Impaired Parents"[Mesh] OR "Child, Orphaned"[Mesh] OR "Child, Institutionalized"[Mesh] OR "Child, Hospitalized"[Mesh] OR "Child, Abandoned"[Mesh] OR "Child, Foster"[Mesh] OR "Battered Child Syndrome"[Mesh] OR girl*)

Web of Science – core collection 16/11/2020: **108**

(TS="COVID-19" OR TS="SARS-COV-2" OR TS="severe acute respiratory syndrome coronavirus 2" OR TS="Middle East Respiratory Syndrome Coronavirus" OR TS="Ebola*") AND (TS=Lockdown* or TS=closure* or TS=shutdown* or TS="stay at home" or TS=curfew* or TS=confinement* or TS=restriction* or TS=ban or TS=prohibition or TS=suppression* or TS=disrupt* or TS=constraint* or TS=Polic*) AND (TS=child or TS=children or TS=woman or TS=women or TS=girl or TS=girls)
Refined by: DOCUMENT TYPES: (ARTICLE OR REVIEW) AND **WEB OF SCIENCE CATEGORIES:** (PUBLIC ENVIRONMENTAL OCCUPATIONAL HEALTH OR EDUCATION SPECIAL OR SOCIAL ISSUES OR ENVIRONMENTAL SCIENCES OR DEVELOPMENT STUDIES OR WOMEN S STUDIES OR ENVIRONMENTAL STUDIES OR MANAGEMENT OR HEALTH CARE SCIENCES SERVICES OR POLITICAL SCIENCE OR EDUCATION EDUCATIONAL RESEARCH OR PUBLIC ADMINISTRATION OR SOCIAL WORK OR SOCIOLOGY OR ECONOMICS OR MULTIDISCIPLINARY SCIENCES OR HEALTH POLICY SERVICES OR LAW OR FAMILY STUDIES OR MEDICAL ETHICS OR PSYCHOLOGY APPLIED OR SOCIAL SCIENCES INTERDISCIPLINARY OR BUSINESS OR PSYCHOLOGY OR PSYCHOLOGY DEVELOPMENTAL) AND **COUNTRIES/REGIONS:** (GUINEA OR TUNISIA OR VIETNAM OR ARGENTINA OR PEOPLES R CHINA OR CAMEROON OR EGYPT OR COLOMBIA OR INDIA OR DEM REP CONGO OR SIERRA LEONE OR MALAYSIA OR EL SALVADOR OR MEXICO OR GAMBIA OR BRAZIL OR NEPAL OR IRAN OR NIGERIA OR MOZAMBIQUE OR RUSSIA OR ALGERIA OR PHILIPPINES OR SOUTH AFRICA OR BOTSWANA OR BULGARIA OR RWANDA OR ECUADOR OR SENEGAL OR GHANA OR LIBERIA OR THAILAND OR PAKISTAN OR JORDAN OR UGANDA OR KENYA OR UKRAINE OR TURKEY OR BANGLADESH OR ZAMBIA)

Timespan: 2000-2020. **Indexes:** SCI-EXPANDED, SSCI, A&HCI, ESCI.

Scopus 16/11/2020 **119**

(TITLE-ABS-KEY ("COVID-19") OR TITLE-ABS-KEY ("SARS-COV-2") OR TITLE-ABS-KEY ("severe acute respiratory syndrome coronavirus 2") OR TITLE-ABS-KEY ("Middle East Respiratory Syndrome Coronavirus") OR TITLE-ABS-KEY ("Ebola*")) AND (TITLE-ABS-KEY (lockdown*) OR TITLE-ABS-KEY (closure*) OR TITLE-ABS-KEY (shutdown) * OR TITLE-ABS-KEY ("stay at home") OR TITLE-ABS-KEY ("impact") OR TITLE-ABS-KEY (curfew*) OR TITLE-ABS-KEY (confinement*) OR TITLE-ABS-KEY (consequence*) OR TITLE-ABS-KEY (restriction*) OR TITLE-ABS-KEY (ban) OR TITLE-ABS-KEY (prohibition) OR TITLE-ABS-KEY (suppression*) OR TITLE-ABS-KEY (disrupt*) OR TITLE-ABS-KEY (constraint*) OR TITLE-ABS-KEY (polic*)) AND (TITLE-ABS-KEY (child*) OR TITLE-ABS-KEY (woman) OR TITLE-ABS-KEY (women) OR TITLE-ABS-KEY (girl*)) AND (LIMIT-TO (AFFILCOUNTRY , "India") OR LIMIT-TO (AFFILCOUNTRY , "China") OR LIMIT-TO (AFFILCOUNTRY , "South Africa") OR LIMIT-TO (AFFILCOUNTRY , "Brazil") OR LIMIT-TO (AFFILCOUNTRY , "Pakistan") OR LIMIT-TO (AFFILCOUNTRY , "Turkey") OR LIMIT-TO (AFFILCOUNTRY , "Bangladesh") OR LIMIT-TO (AFFILCOUNTRY , "Indonesia") OR LIMIT-TO (AFFILCOUNTRY , "Iran") OR LIMIT-TO (AFFILCOUNTRY , "Nigeria") OR LIMIT-TO (AFFILCOUNTRY , "Argentina") OR LIMIT-TO (AFFILCOUNTRY , "Malaysia") OR LIMIT-TO (AFFILCOUNTRY , "Mexico") OR LIMIT-TO (AFFILCOUNTRY , "Philippines") OR LIMIT-TO (AFFILCOUNTRY , "Russian Federation") OR LIMIT-TO (AFFILCOUNTRY , "Thailand") OR LIMIT-TO (AFFILCOUNTRY , "Afghanistan") OR LIMIT-TO (AFFILCOUNTRY , "Botswana") OR LIMIT-TO (AFFILCOUNTRY , "Cameroon") OR LIMIT-TO (AFFILCOUNTRY , "Egypt") OR LIMIT-TO (AFFILCOUNTRY , "Jordan") OR LIMIT-TO (AFFILCOUNTRY , "Kenya") OR LIMIT-TO (AFFILCOUNTRY , "Lebanon") OR LIMIT-TO (AFFILCOUNTRY , "Liberia") OR LIMIT-TO (AFFILCOUNTRY , "Mauritius")

OR LIMIT-TO (AFFILCOUNTRY , "Morocco") OR LIMIT-TO (AFFILCOUNTRY , "Nepal") OR LIMIT-TO (AFFILCOUNTRY , "Palestine") OR LIMIT-TO (AFFILCOUNTRY , "Papua New Guinea") OR LIMIT-TO (AFFILCOUNTRY , "Suriname") OR LIMIT-TO (AFFILCOUNTRY , "Tunisia") OR LIMIT-TO (AFFILCOUNTRY , "Ukraine") OR LIMIT-TO (AFFILCOUNTRY , "Undefined")) AND (LIMIT-TO (DOCTYPE , "ar") OR LIMIT-TO (DOCTYPE , "re")) AND (LIMIT-TO (SUBJAREA , "MEDI") OR LIMIT-TO (SUBJAREA , "SOCI") OR LIMIT-TO (SUBJAREA , "PSYC") OR LIMIT-TO (SUBJAREA , "ENVI") OR LIMIT-TO (SUBJAREA , "AGRI") OR LIMIT-TO (SUBJAREA , "MULT") OR LIMIT-TO (SUBJAREA , "BUSI"))

Scielo 24

("COVID-19" OR "SARS-COV-2" OR "severe acute respiratory syndrome coronavirus 2" OR "Middle East Respiratory Syndrome Coronavirus" OR "Ebola*") AND (lockdown* OR closure* OR shutdown OR "stay at home" OR "impact" OR curfew* OR confinement* OR consequence* OR restriction* OR ban OR prohibition OR suppression* OR disrupt* OR constraint* OR polic*) AND (child* OR woman OR women OR girl*)

Filters on wok_subject_categories:("health policy & services" OR "health care sciences & services" OR "public, environmental & occupational health" OR "medical ethics" OR "psychology, multidisciplinary" OR "sociology" OR "management" OR "psychology, social" OR "public administration" OR "social sciences, interdisciplinary" OR "women's studies")

Cochrane Library 84

("COVID-19" OR "SARS-COV-2" OR "severe acute respiratory syndrome coronavirus 2" OR "Middle East Respiratory Syndrome Coronavirus" OR "Ebola*") AND (lockdown* OR closure* OR shutdown OR "stay at home" OR "impact" OR curfew* OR confinement* OR consequence* OR restriction* OR ban OR prohibition OR suppression* OR disrupt* OR constraint* OR polic*) AND (child* OR woman OR women OR girl*)

PDQ Evidence – 4

("COVID-19" OR "SARS-COV-2" OR "severe acute respiratory syndrome coronavirus 2" OR "Middle East Respiratory Syndrome Coronavirus" OR "Ebola*") AND (lockdown* OR closure* OR shutdown OR "stay at home" OR "impact" OR curfew* OR confinement* OR consequence* OR restriction* OR ban OR prohibition OR suppression* OR disrupt* OR constraint* OR polic*) AND (child* OR woman OR women OR girl*)
