# Supplementary material for: Epidemics, Lockdown Measures and Vulnerable Populations: A Mixed-Methods Systematic Review of the Evidence of Impacts on Mother and Child Health in Low-and Lower-Middle-Income Countries
Source: Int J Health Policy Manag. 2021 Nov 7;11(10):2003–21. doi: 10.34172/ijhpm.2021.155 (PMC9808285; doi:10.34172/ijhpm.2021.155)
Supplement: Supplementary file 5 — Data Extraction and Quality Appraisal Form. [file ijhpm-11-2003-s005.pdf]

Article title: Epidemics, Lockdown Measures and Vulnerable Populations: A Mixed-Methods Systematic Review of the Evidence of Impacts on Mother and Child Health in Lowand Lower-Middle-Income Countries

Journal name: International Journal of Health Policy and Management (IJHPM)

Authors' information: Giuliano Russo[1]*, Tiago Silva Jesus[2], Kevin Deane[3], Abdinasir Yusuf Osman[4], David McCoy[1]

[1]Wolfson Institute of Population Health, Queen Mary University of London, London, UK. [2]Global Health & Tropical Medicine, Instituto de Higiene e Medicina Tropical, Nova University of Lisbon, Lisbon, Portugal.

[3]Faculty of Arts & Social Sciences, School of Social Sciences & Global Studies, Open University, Milton Keynes, UK.

[4]Royal Veterinary College, University of London, London, UK

(*Corresponding author: g.russo@qmul.ac.uk)

Supplementary file 5. Data Extraction and Quality Appraisal Form

Table S1. Data extraction form for SLR on the health impact of lockdown measures for women and children in LMICs

Title of the paper:	Burden of acute respiratory disease of epidemic and pandemic potential in the WHO Eastern Mediterranean Region: A literature review
Author(s) and date:	A. Abubakar , M. Malik , R.G. Pebody , A.A. Elkholy , W. Khan , A. Bellos and P. Mala
Quality appraisal according to the relevant Johanna Briggs Institute tool (please cross out or delete accordingly)	Exclude but not due to JBI criteria
Epidemic of reference:	No specific epidemic mentioned
Research methods employed (e.g. qualitative interviews; modelling; surveys; systematic review)	Systematic review
Specific lockdown measure investigated in the study (e.g. generic quarantine or unspecified lockdown measure; curfew; stay at home policy; travel restrictions; school closures; market and trade shutdowns; social distancing)	Not about the impact of lockdown
Broad area of impact explored in the study (please cross out or delete as appropriate)	Description of the burden of acute respiratory diseases in the WHO east Mediterranean region

Evidence of impact to the general population from lockdown measures (e.g. 10% decrease of disposable income before and after the peak of the epidemic; 30% reduction of hospital visits for maternal services)	
Specific evidence of impact on children (e.g. 30% increase of weight during two weeks of implementation of stay at home policy; or: 'calculated 365,000 extra malaria deaths calculated as a consequence of disruption of bed nets distribution)	
Specific evidence of impact on women (e.g. 20% reduction of female employment in the fishing sector, as a result of decreased demand for fish during the epidemic)	
Relevance and importance of the paper for our own systematic review	exclude
Other comments or issues to be noted about this paper	

Table S1. Data extraction form for SLR on the health impact of lockdown measures for women and children in LMICs

Title of the paper:	COVID-19 Home Confinement Negatively Impacts Social Participation and Life Satisfaction: A Worldwide Multicenter Study
Author(s) and date:	Ammar et al (too many to list!)
Quality appraisal according to the relevant Johanna Briggs Institute tool (please cross out or delete accordingly)	☐ Include
Epidemic of reference:	Covid
Research methods employed (e.g. qualitative interviews; modelling; surveys; systematic review)	Online Survey<br>N=1047 from 47 countries<br><br>Shared online and via networks, so not a representative sample per se
Specific lockdown measure investigated in the study (e.g. generic quarantine or	No specific measure, talks about 'social confinement'<br><br>Paper aims to understand the impact of 'home confinement'

unspecified lockdown measure; curfew; stay at home policy; travel restrictions; school closures; market and trade shutdowns; social distancing)	
Broad area of impact explored in the study (please cross out or delete as appropriate)	1. Mental Health;<br>2. Dietary patterns and nutrition;<br>3. Physical exercise and impacts on obesity and specific diseases such as diabetes;<br>4. Disruption of routine services for mother and child services;<br>5. Demand for healthcare / Healthcare seeking behaviour;<br>6. Impact on partner relationships, sexual and reproductive behaviour;<br>7. Increases in social vulnerability, poverty and impact on human development;<br>8. Other (please specify).
Evidence of impact to the general population from lockdown measures (e.g. 10% decrease of disposable income before and after the peak of the epidemic; 30% reduction of hospital visits for maternal services)	Statistical analysis showed the total score of SSPQL (Short Social Participation Questionnaire-Lockdowns) (decreased significantly by 42% "during" compared to "before" home confinement ($t = 69.19$, $p < 0.001$, $d = 2.14$).<br><br>SLSQL (Short Life Satisfaction Questionnaire—Lockdowns) decreased significantly by 16% "during" compared to "before" home confinement ($t = 21.05$, $p < 0.001$, $d = 0.65$).<br><br>SLSQL made up of:<br><br>1. In most ways my life is close to my ideal.<br>2. So far, I have gotten the important things I want in life.<br>3. I am satisfied with my life.<br><br>So the headline is that respondents reported lower life satisfaction during home confinement compared to before home confinement!
Specific evidence of impact on children (e.g. 30% increase of weight during two weeks of implementation of stay at home policy; or: 'calculated 365,000 extra malaria deaths calculated as a consequence of disruption of bed nets distribution)	
Specific evidence of impact on women (e.g. 20% reduction of female employment in the fishing sector, as a result of decreased demand for fish during the epidemic)	Women comprise 53.8% of the sample

Relevance and importance of the paper for our own systematic review	☐ Paper of some importance for some sections of the review
Other comments or issues to be noted about this paper	

Table 1: Data extraction form for SLR on the health impact of lockdown measures for women and children in LMICs

Title of the paper:	The potential impact of the COVID-19 pandemic on child growth and development: a systematic review
Author(s) and date:	Arantes de Araújo et al (2020)
Quality appraisal according to the relevant Johanna Briggs Institute tool (please cross out or delete accordingly)	☐ Seek further information
Epidemic of reference:	COVID-19
Research methods employed (e.g. qualitative interviews; modelling; surveys; systematic review)	Systematic review
Specific lockdown measure investigated in the study (e.g. generic quarantine or unspecified lockdown measure; curfew; stay at home policy; travel restrictions; school closures; market and trade shutdowns; social distancing)	Social restrictions, shutdowns, and school closures
Broad area of impact explored in the study (please cross out or delete as appropriate)	9. Mental Health;<br>10. Dietary patterns and nutrition;<br>11. Physical exercise and impacts on obesity and specific diseases such as diabetes;<br>12. Disruption of routine services for mother and child services;<br>13. Demand for healthcare / Healthcare seeking behaviour;<br>14. Impact on partner relationships, sexual and reproductive behaviour;<br>15. Increases in social vulnerability, poverty and impact on human development;<br>16. Other (please specify).
Evidence of impact to the general population from lockdown measures (e.g. 10% decrease of disposable income before and after the peak of the epidemic; 30% reduction of hospital visits for maternal services)	

Specific evidence of impact on children (e.g. 30% increase of weight during two weeks of implementation of stay at home policy; or: 'calculated 365,000 extra malaria deaths calculated as a consequence of disruption of bed nets distribution)	The review reports findings from two cases in Sierra Leone and Nigeria where children were more susceptible of mental healh issues because of the Ebola pandemic – but not necessarily because of the lockdown measures…<br><br>Interesting discussion on the lack of evidence on the effectiveness of school closures from the US
Specific evidence of impact on women (e.g. 20% reduction of female employment in the fishing sector, as a result of decreased demand for fish during the epidemic)	In addressing the challenges for pregnant women during pandemics, fetal losses caused by possible maternal infection have been investigated; however, losses due to secondary causes, such as mood changes during pregnancy and in the postpartum period are also relevant.
Relevance and importance of the paper for our own systematic review	☐ Secondary paper
Other comments or issues to be noted about this paper	The quality was dubious, but I woud not exclude the study completely. But will not use its findings extensively.

Table S1. Data extraction form for SLR on the health impact of lockdown measures for women and children in LMICs

Title of the paper:	Consequences of calamities and their management: The case of COVID-19 pandemic and flooding on inland capture fisheries in Kenya
Author(s) and date:	Christopher Mulanda Aura, Chrisphine S. Nyamweya , Cyprian O. Odoli , Horace Owiti ,James M. Njiru , Patrick W. Otuo , Edna Waithaka , John Malala
Quality appraisal according to the relevant Johanna Briggs Institute tool (please cross out or delete accordingly)	☐ Include
Epidemic of reference:	Covid-19
Research methods employed (e.g. qualitative interviews; modelling; surveys; systematic review)	Socio-economic survey with purposively sampled stakeholders from across the fish value chain, including:<br><br>Input providers (boat builders and engine repairers); producers (fishers); middlemen (fish traders and processors); ancillary service providers (transporters and community health workers); and managers (Beach Manage ment<br>Units' official)<br><br>N=336 across 4 different lakes in Kenya<br><br>80% of respondents either fishers or traders
Specific lockdown measure investigated in the study (e.g. generic quarantine or unspecified lockdown	Measures mentioned"<br><br>"These included restricting international travel, cessation of movement from some cities including the capital city of Nairobi,

measure; curfew; stay at home policy; travel restrictions; school closures; market and trade shutdowns; social distancing)	imposing curfews, social distancing and closure of areas of mass gathering such as schools and places of worship, and dusk to dawn curfews”
Broad area of impact explored in the study (please cross out or delete as appropriate)	17. Increases in social vulnerability, poverty and impact on human development;<br>18.
Evidence of impact to the general population from lockdown measures (e.g. 10% decrease of disposable income before and after the peak of the epidemic; 30% reduction of hospital visits for maternal services)	These measures, and specifically the cessation of movement to cities that are the main fish markets, curfews and social distancing affected fishing trips and duration, disrupted the fish value chain and affected the livelihoods of fishers.<br><br>79% of respondents said that COVID measures impacted the fishing industry ‘very much’ (as opposed to ‘much’ or ‘a little’)<br><br>Fish traders and processors affected the most due to closures of markets<br><br>“The COVID-19 pandemic containment regulations impacted fishing and fish trade in all the freshwater lakes examined, for instance, dusk to dawn curfew (66%, n = 128), lock-down to major markets such as Nairobi and Mombasa (28%, n = 55),”<br><br>“fishing time was reduced (76%, n = 116) as well as fishing trips per week from an average of seven to five trips (n = 103) due to the COVID-19 pandemic. The reduction in fishing time and trips were occasioned by the dusk to dawn (1900 h _ 0500 h) curfew. In addition, during the pandemic, there was a notable decline in the average crew (fishing inputs) and boat fuel (consumable) used in fishing activities<br>resulting into a cross-cutting decline in catch quantities and prices”
Specific evidence of impact on children (e.g. 30% increase of weight during two weeks of implementation of stay at home policy; or: ‘calculated 365,000 extra malaria deaths calculated as a consequence of disruption of bed nets distribution)	
Specific evidence of impact on women (e.g. 20% reduction of female employment in the fishing sector, as a result of decreased demand for fish during the epidemic)	

Relevance and importance of the paper for our own systematic review	☐ Secondary paper
Other comments or issues to be noted about this paper	Shows impacts on livelihoods that will have further implications for women and child health

Table 2: Data extraction form for SLR on the health impact of lockdown measures for women and children in LMICs

Title of the paper:	Predicting the Impact of COVID-19 and the Potential Impact of the Public Health Response on Disease Burden in Uganda
Author(s) and date:	Bell et al (2020)
Quality appraisal according to the relevant Johanna Briggs Institute tool (please cross out or delete accordingly)	☐ Include
Epidemic of reference:	COVID-19
Research methods employed (e.g. qualitative interviews; modelling; surveys; systematic review)	Modelling publicly available data for burden of disease in Uganda
Specific lockdown measure investigated in the study (e.g. generic quarantine or unspecified lockdown measure; curfew; stay at home policy; travel restrictions; school closures; market and trade shutdowns; social distancing)	Generic social distancing policies (involving stay at home, travel restrictions, but also disruption of services)
Broad area of impact explored in the study (please cross out or delete as appropriate)	19. Deaths.
Evidence of impact to the general population from lockdown measures (e.g. 10% decrease of disposable income before and after the peak of the epidemic; 30% reduction of hospital visits for maternal services)	Decline of 75% in reporting of new AIDS cases and initiation of ART therapy. The authors predicted an overall loss of 475,319 DALYs from disruption of therapy and detection of new cases. Different scenarios of mortality (from 3,000 to 31,000), and DALY lost to malaria (most of them for children), ranging from 257,000 to 2,450,000.
Specific evidence of impact on children (e.g. 30% increase of weight during two weeks of implementation of stay at home policy; or: 'calculated 365,000 extra malaria deaths calculated as a consequence of disruption of bed nets distribution)	

Specific evidence of impact on women (e.g. 20% reduction of female employment in the fishing sector, as a result of decreased demand for fish during the epidemic)	Maternal mortality. A 29% (28,939) reduction in facility deliveries is recorded in the Ministry of Health Uganda data in March compared with January 2020, 28% less than the 12-month average for 2019. Over the same period, an 82% increase in maternal mortality was recorded (from 92 to 167 women), an increase of 87% over the 12-month 2019 average of 89.5 (Figure 1, Supplemental Table S4). An excess 486 deaths are predicted for a 6-month period, incurring 31,343 DALYs lost.
Relevance and importance of the paper for our own systematic review	☐ Key paper for the review
Other comments or issues to be noted about this paper	Very important paper, particularly as it focuses on Uganda.

Table S1. Data extraction form for SLR on the health impact of lockdown measures for women and children in LMICs

Title of the paper:	Challenges for work–life balance during COVID-19 induced nationwide lockdown: exploring gender difference in emotional exhaustion in the Indian setting
Author(s) and date:	Bhumika
Quality appraisal according to the relevant Johanna Briggs Institute tool (please cross out or delete accordingly)	☐ Include<br>☐
Epidemic of reference:	Covid-19
Research methods employed (e.g. qualitative interviews; modelling; surveys; systematic review)	Online survey with "employed individuals (employed on payroll) who were working from home during the COVID-19-induced countrywide lockdown. The selected participants were full time employees, and their employers expected them to be available online at least for the normal office hours during the working days. The research participants belonged to a heterogeneous sample of industries, including IT, education, finance and automobile"<br><br>N=180
Specific lockdown measure investigated in the study (e.g. generic quarantine or unspecified lockdown measure; curfew; stay at home policy; travel restrictions; school closures; market and trade shutdowns; social distancing)	No specific measure<br><br>"On 24 March, Indian Prime Minister announced a 21 days' nationwide lockdown till 3 April 2020, which got further extended till 3 May 2020."
Broad area of impact explored in the study (please cross out or delete as appropriate)	20. Mental Health;
Evidence of impact to the general population from lockdown measures (e.g. 10%	Work Interference with person like (WIPL) (b = 0.69, $p < 0.05$, 95%CI = 0.55 to 0.82) was found to be positively related to emotional exhaustion.

decrease of disposable income before and after the peak of the epidemic; 30% reduction of hospital visits for maternal services)	Personal life interfering with work (b = 0.81, p < 0.05, 95% CI = 0.61 to 1.01) was found to be positively related to emotional exhaustion.
Specific evidence of impact on children (e.g. 30% increase of weight during two weeks of implementation of stay at home policy; or: 'calculated 365,000 extra malaria deaths calculated as a consequence of disruption of bed nets distribution)	
Specific evidence of impact on women (e.g. 20% reduction of female employment in the fishing sector, as a result of decreased demand for fish during the epidemic)	The relationship between personal life interfering with work and emotional exhaustion was moderated by gender such that it was stronger for women than men.<br><br>The authors put this down to<br><br>"family and work are considered to be the primary domains for women, whereas work becomes the primary domain for men. Due to such prescribed gender specific roles, working women might experience a double bind when family and work are equally demanding. In the case of dual career phenomena, couples juggle between work and family demands, and in such cases, women might feel a double bind due to the prescribed gender specific roles existing in a particular society. The working women might have to struggle to create a balance between work and family, particularly when both simultaneously demand higher attention and efforts."
Relevance and importance of the paper for our own systematic review	☐ Secondary paper
Other comments or issues to be noted about this paper	Some limited statistical imapcts on women.

Table 3: Data extraction form for SLR on the health impact of lockdown measures for women and children in LMICs

Title of the paper:	Preventive malaria treatment for contacts of patients with Ebola virus disease in the context of the west Africa 2014-15 Ebola virus disease response: an economic analysis
Author(s) and date:	Carias et al (2016)
Quality appraisal according to the relevant Johanna Briggs Institute tool (please cross out or delete accordingly)	☐ Include
Epidemic of reference:	Ebola
Research methods employed (e.g. qualitative interviews;	Economic evaluation of preventive malaria treatment for all contacts of patients with Ebola virus disease

modelling; surveys; systematic review)	
Specific lockdown measure investigated in the study (e.g. generic quarantine or unspecified lockdown measure; curfew; stay at home policy; travel restrictions; school closures; market and trade shutdowns; social distancing)	Quarantine, contact tracing and isolating patients
Broad area of impact explored in the study (please cross out or delete as appropriate)	21. Demand for healthcare / Healthcare seeking behaviour;<br>22. Other – treating preventively for malaria patients isolated for Ebola to avoid being admitted to ETU
Evidence of impact to the general population from lockdown measures (e.g. 10% decrease of disposable income before and after the peak of the epidemic; 30% reduction of hospital visits for maternal services)	Administration of preventive ACTs to contacts of patients with Ebola virus disease was cost saving for contacts of all Ages, as it avoided hospitalization or being mistakenly admitted to Ebola Treatment Units
Specific evidence of impact on children (e.g. 30% increase of weight during two weeks of implementation of stay at home policy; or: 'calculated 365,000 extra malaria deaths calculated as a consequence of disruption of bed nets distribution)	The intervention was calculated to be cost saving in contacts in areas with malaria parasite prevalence in children aged 2–10 years as low as 10%
Specific evidence of impact on women (e.g. 20% reduction of female employment in the fishing sector, as a result of decreased demand for fish during the epidemic)	N/A
Relevance and importance of the paper for our own systematic review	☐ Secondary paper
Other comments or issues to be noted about this paper	This study looks at the effects of being put in quarantine/isolation and displaying malaria symptoms that can be mistaken for Ebola, particularly fo rchildren. We can make an argument that once in isolation, patients do not seek/are not given services to correctly diagnose malaria

Table 4: Data extraction form for SLR on the health impact of lockdown measures for women and children in LMICs

Title of the paper:	Impacts of a National Lockdown on Smallholder Farmers' Income and Food Security: Empirical Evidence from Two States in India
Author(s) and date:	Ceballos, Kannan and Kramer (2020)
Quality appraisal according to the relevant Johanna Briggs Institute tool (please cross out or delete accordingly)	☐ Include
Epidemic of reference:	COVID-19
Research methods employed (e.g. qualitative interviews; modelling; surveys; systematic review)	Phone-based surveys on disruptions to agricultural production and food security, complemented by key informants interviews and desk review
Specific lockdown measure investigated in the study (e.g. generic quarantine or unspecified lockdown measure; curfew; stay at home policy; travel restrictions; school closures; market and trade shutdowns; social distancing)	Restriction of movement and travel across states for seasonal workers. Closure of local markets for procurement of crops (wheat and black gram).
Broad area of impact explored in the study (please cross out or delete as appropriate)	23. Dietary patterns and nutrition;<br>24. Increases in social vulnerability, poverty and impact on human development;<br>25. Reduction of disposable income
Evidence of impact to the general population from lockdown measures (e.g. 10% decrease of disposable income before and after the peak of the epidemic; 30% reduction of hospital visits for maternal services)	Because of the shortages of seasonal labourers linked to travelling restrictions, 41-80% of farmers responded that they had to spend more on labour to harvest. Because of the closures of the local markets (modis), 61-74% responded they had to store their harvest and sell in the future. No difference in access to food was reported before and after the lockdowns.
Specific evidence of impact on children (e.g. 30% increase of weight during two weeks of implementation of stay at home policy; or: 'calculated 365,000 extra malaria deaths calculated as a consequence of disruption of bed nets distribution)	N/A
Specific evidence of impact on women (e.g. 20% reduction of female employment in the fishing sector, as a result of	N/A

decreased demand for fish during the epidemic)	
Relevance and importance of the paper for our own systematic review	☐ Secondary paper
Other comments or issues to be noted about this paper	

Table S1. Data extraction form for SLR on the health impact of lockdown measures for women and children in LMICs

Title of the paper:	Decrease in Hospitalizations and Increase in Deaths during the Covid-19 Epidemic in a Pediatric Hospital, Yaounde-Cameroon and Prediction for the Coming Months
Author(s) and date:	David Chelo , Isabelle Mekone Nkwelle , Félicitée Nguefack , Hubert Désiré, Mbassi Awa , Dominique Enyama , Séraphin Nguefack , Diomède Noukeu Njinkui , Jocelyn Tony Nengom , Georges Nguefack-Tsague & Paul Olivier Koki Ndombo
Quality appraisal according to the relevant Johanna Briggs Institute tool (please cross out or delete accordingly)	☐ Include
Epidemic of reference:	Covid-19
Research methods employed (e.g. qualitative interviews; modelling; surveys; systematic review)	A descriptive and retrospective cross-sectional study was carried out using hospitalization and death statistics collected from a pediatric hospital. We compared the data before and after the pandemic and made predictions for the next 12months.
Specific lockdown measure investigated in the study (e.g. generic quarantine or unspecified lockdown measure; curfew; stay at home policy; travel restrictions; school closures; market and trade shutdowns; social distancing)	No specific measure used<br><br>The study was conducted from 1st to 30th June, 2020 and covered the period from January 1st, 2016 to May 31st, 2020.
Broad area of impact explored in the study (please cross out or delete as appropriate)	26. Disruption of routine services for mother and child services;
Evidence of impact to the general population from lockdown measures (e.g. 10% decrease of disposable income before and after the peak of the epidemic; 30% reduction of hospital visits for maternal services)	

Specific evidence of impact on children (e.g. 30% increase of weight during two weeks of implementation of stay at home policy; or: 'calculated 365,000 extra malaria deaths calculated as a consequence of disruption of bed nets distribution)	27% and 47% drop in hospitalizations during the months of April and May 2020 respectively as compared to the same period in 2019 (p¼0.00001).<br><br>Mortality doubled during the months of April and May 2020 with 9.9% and 11.2% respectively of hospital deaths compared to 4.9% (p¼0.0008) and 5.1%(p¼0.0001) during the same period of the previous year.
Specific evidence of impact on women (e.g. 20% reduction of female employment in the fishing sector, as a result of decreased demand for fish during the epidemic)	
Relevance and importance of the paper for our own systematic review	☐ Key paper for the review
Other comments or issues to be noted about this paper	Concrete data of the impact of the epidemic (which will include control measures but also other things)

Table S1. Data extraction form for SLR on the health impact of lockdown measures for women and children in LMICs

Title of the paper:	A Cross-Sectional Study on Cognitive Errors and Obsessive-Compulsive Disorders among Young People During the Outbreak of Coronavirus Disease 2019
Author(s) and date:	Elham Darvishi, Solmaz Golestan, Farangis Demehri and Sheida Jamalnia, 2020
Quality appraisal according to the relevant Johanna Briggs Institute tool (please cross out or delete accordingly)	☐ Include
Epidemic of reference:	Covid-19
Research methods employed (e.g. qualitative interviews; modelling; surveys; systematic review)	Cross sectional survey (n=150)
Specific lockdown measure investigated in the study (e.g. generic quarantine or unspecified lockdown measure; curfew; stay at home policy; travel restrictions; school closures; market and trade shutdowns; social distancing)	No specific lockdown measure. The issue of lockdown is mentioned early on but no background to the lockdown measures implemented in Iran was given.
Broad area of impact explored in the study (please cross out or delete as appropriate)	27. Mental Health;

Evidence of impact to the general population from lockdown measures (e.g. 10% decrease of disposable income before and after the peak of the epidemic; 30% reduction of hospital visits for maternal services)	
Specific evidence of impact on children (e.g. 30% increase of weight during two weeks of implementation of stay at home policy; or: 'calculated 365,000 extra malaria deaths calculated as a consequence of disruption of bed nets distribution)	The sample age range was 13-19 high school students<br><br>67.3% of the subjects may have demonstrated OCD symptoms<br><br>The prevalence of obsessive-compulsive disorder symptoms in female students was slightly higher than in male students (72.1% compared to 60.3%)<br><br>highest prevalence of obsessive-compulsive disorder symptom belonged to the washing compulsion<br><br>For those exhibiting some OCD symptons, higher levels of Cognitive Errors were reported
Specific evidence of impact on women (e.g. 20% reduction of female employment in the fishing sector, as a result of decreased demand for fish during the epidemic)	The prevalence of obsessive-compulsive disorder symptoms in female students was slightly higher than in male students (72.1% compared to 60.3%)
Relevance and importance of the paper for our own systematic review	■ Paper of some importance for some sections of the review
Other comments or issues to be noted about this paper	Not much detail on lockdown. No idea how the random sample was conducted!<br>I am also not sure I fully understand their data, a difficult paper to read.

Table S1. Data extraction form for SLR on the health impact of lockdown measures for women and children in LMICs

Title of the paper:	Early impact of COVID-19 lockdown on children's sleep: a 4-week longitudinal study
Author(s) and date:	Antonio Dellagiulia, Francesca Lionetti, Mirco Fasolo, Chiara Verderame,; Alessandra Sperati, Guido Alessandri,
Quality appraisal according to the relevant Johanna Briggs Institute tool (please cross out or delete accordingly)	Exclude but on the basis of this study being conducted in Italy, not a LMIC
Epidemic of reference:	
Research methods employed (e.g. qualitative interviews;	

modelling; surveys; systematic review)	
Specific lockdown measure investigated in the study (e.g. generic quarantine or unspecified lockdown measure; curfew; stay at home policy; travel restrictions; school closures; market and trade shutdowns; social distancing)	
Broad area of impact explored in the study (please cross out or delete as appropriate)	28. Mental Health;<br>29. Dietary patterns and nutrition;<br>30. Physical exercise and impacts on obesity and specific diseases such as diabetes;<br>31. Disruption of routine services for mother and child services;<br>32. Demand for healthcare / Healthcare seeking behaviour;<br>33. Impact on partner relationships, sexual and reproductive behaviour;<br>34. Increases in social vulnerability, poverty and impact on human development;<br>35. Other (please specify).
Evidence of impact to the general population from lockdown measures (e.g. 10% decrease of disposable income before and after the peak of the epidemic; 30% reduction of hospital visits for maternal services)	
Specific evidence of impact on children (e.g. 30% increase of weight during two weeks of implementation of stay at home policy; or: 'calculated 365,000 extra malaria deaths calculated as a consequence of disruption of bed nets distribution)	
Specific evidence of impact on women (e.g. 20% reduction of female employment in the fishing sector, as a result of decreased demand for fish during the epidemic)	
Relevance and importance of the paper for our own systematic review	☐ Key paper for the review<br>☐ Paper of some importance for some sections of the review<br>☐ Secondary paper
Other comments or issues to be noted about this paper	

Table S1. Data extraction form for SLR on the health impact of lockdown measures for women and children in LMICs

Author(s) and date:	A. Kc, R. Gurung, M. V. Kinney, A. K. Sunny, M. Moinuddin, O. Basnet, P. Paudel, P. Bhattarai, K. Subedi, M. P. Shrestha, J. E. Lawn and M. Målqvist;
Year	2020
Title of the paper:	Effect of the COVID-19 pandemic response on intrapartum care, stillbirth, and neonatal mortality outcomes in Nepal: a prospective observational study
Study design	a prospective observational study
Setting	hospital
PS	Women
Study location	Nepal
Quality appraisal according to the relevant Johanna Briggs Institute tool (please cross out or delete accordingly)	☐ Include
Epidemic of reference:	Covid-19
Research methods employed (e.g. qualitative interviews; modelling; surveys; systematic review)	Modelling; quantitaive surveillance
Specific lockdown measure investigated in the study (e.g. generic quarantine or unspecified lockdown measure; curfew; stay at home policy; travel restrictions; school closures; market and trade shutdowns; social distancing)	generic quarantine or unspecified lockdown measure
Broad area of impact explored in the study (please cross out or delete as appropriate)	Disruption of routine services for mother and child services;
Evidence of impact to the general population from lockdown measures (e.g. 10% decrease of disposable income before and after the peak of the epidemic; 30% reduction of hospital visits for maternal services)	the mean weekly number of births decreased from 1261·1 births (SE 66·1) before lockdown to 651·4 births (49·9) during lockdown—a reduction of 52·4%;
Specific evidence of impact on children (e.g. 30% increase of weight during two weeks of implementation of stay at home policy; or: 'calculated 365,000 extra malaria deaths calculated as a consequence of disruption of bed nets distribution)	The institutional stillbirth rate increased from 14 per 1000 total births before lockdown to 21 per 1000 total births during lockdown (p=0·0002), and institutional neonatal mortality increased from 13 per 1000 livebirths to 40 per 1000 livebirths (p=0·0022); The average weekly reduction in institutional births during lockdown was 7·4%, with a total decrease of 52·4% by the end of lockdown
Specific evidence of impact on women (e.g. 20% reduction of female employment in the fishing sector, as a result of decreased demand for fish during the epidemic)	In terms of quality of care, intrapartum fetal heart rate monitoring decreased by 13·4% (–15·4 to –11·3; p<0·0001), and breastfeeding within 1 h of birth decreased by 3·5% (–4·6 to –2·6; p=0·0032); Decrease in attendance was seen among the more disadvantaged ethnic group Madhesi during lockdown (1228 [17·1%]) compared with before lockdown (2840 [21·5%]; p=0·0015); The proportion of women who had a complication during admission increased from 6·7% (n=884) before lockdown to 8·7% (n=587) during lockdown (p=0·0126). The proportion of women whose labour was induced increased from 17·1% (n=2258) before lockdown to 32·1% (n=2282) during lockdown (p<0·0001). The proportion of women who had caesarean section increased from 24·5% (n=3234) before lockdown to 26·2% (n=1879) during lockdown (p=0·0075).
Relevance and importance of the paper for our own systematic review	☐ Key paper for the review
Other comments or issues to be noted about this paper	

Author(s) and date:	Fitzgerald, F., Awonuga, W., Shah, T., & Youkee, D. (2016). Ebola response in Sierra Leone: The impact on children. Journal of Infection, 72, S6-S12.
Year	2016
Title of the paper:	Ebola response in Sierra Leone: The impact on children
Study design	Survey
Setting	Ebola Holding Unit
PS	Children
Study location	Sierra Leone
Quality appraisal according to the relevant Johanna Briggs Institute tool (please cross out or delete accordingly)	Exclude
Epidemic of reference:	Ebola
Research methods employed (e.g. qualitative interviews; modelling; surveys; systematic review)	Not Clear
Specific lockdown measure investigated in the study (e.g. generic quarantine or unspecified lockdown measure; curfew; stay at home policy; travel restrictions; school closures; market and trade shutdowns; social distancing)	generic quarantine or unspecified lockdown measure
Broad area of impact explored in the study (please cross out or delete as appropriate)	Disruption of routine services for mother and child services;
Evidence of impact to the general population from lockdown measures (e.g. 10% decrease of disposable income before and after the peak of the epidemic; 30% reduction of hospital visits for maternal services)	The provision of care to unaccompanied children in a Red Zone (full barrier nursing, where risk of Ebola exposure was judged to be highest) environment extremely challenging. many children had already lost family members to EVD, meant that approximately 40% of children were admitted unaccompanied to EHU
Specific evidence of impact on children (e.g. 30% increase of weight during two weeks of implementation of stay at home policy; or: 'calculated 365,000 extra malaria deaths calculated as a consequence of disruption of bed nets distribution)	difficulty of PPE; PPE hinders effective communication with a child as it makes any worker look terrifying; PPE is flimsy; Ambulant unaccompanied children are difficult to control increasing the risk of cross-contamination and EVD transmission.; Red Zones are full of risks beyond EVD exposure, including sharps bins and buckets of strong chlorine solution that children may mistake for a bath or attempt to drink, both situations; children trying to escape out of the Red Zone and back to their parents.
Specific evidence of impact on women (e.g. 20% reduction of female employment in the fishing sector, as a result of decreased demand for fish during the epidemic)	Forbidding asymptomatic mothers from accompanying their unwell children into EHUs.
Relevance and importance of the paper for our own systematic review	Secondary paper
Other comments or issues to be noted about this paper	

Author(s) and date:	A. Delamou, A. M. E. Ayadi, S. Sidibe, T. Delvaux, B. S. Camara, S. D. Sandouno, A. H. Beavogui, G. W. Rutherford, J. Okumura, W. H. Zhang and V. De Brouwere
Year	2017
Title of the paper:	Effect of Ebola virus disease on maternal and child health services in Guinea: a retrospective observational cohort study
Study design	a retrospective observational cohort study
Setting	public health facilities
PS	Woman & children
Study location	Guinea
Quality appraisal according to the relevant Johanna Briggs Institute tool (please cross out or delete accordingly)	☐ Include
Epidemic of reference:	Ebola
Research methods employed (e.g. qualitative interviews; modelling; surveys; systematic review)	quantitaive surveillance
Specific lockdown measure investigated in the study (e.g. generic quarantine or unspecified lockdown measure; curfew; stay at home policy; travel restrictions; school closures; market and trade shutdowns; social distancing)	generic quarantine or unspecified lockdown measure
Broad area of impact explored in the study (please cross out or delete as appropriate)	Disruption of routine services for mother and child services;
Evidence of impact to the general population from lockdown measures (e.g. 10% decrease of disposable income before and after the peak of the epidemic; 30% reduction of hospital visits for maternal services)	Most maternal and child health indicators significantly declined during the Ebola virus disease outbreak in 2014. Despite a reduction in this negative trend in the post-outbreak period, the use of essential maternal and child health services have not recovered to their pre-outbreak levels, nor are they all on a course that suggests that they will recover without targeted interventions.
Specific evidence of impact on children (e.g. 30% increase of weight during two weeks of implementation of stay at home policy; or: 'calculated 365,000 extra malaria deaths calculated as a consequence of disruption of bed nets distribution)	The greatest reductions were noted for polio and tuberculosis at –3594 (95% CI –4811 to –2377; p<0·0001) and –3048 (95% CI –5879 to –216; p=0·0362) fewer vaccines administered, respectively.
Specific evidence of impact on women (e.g. 20% reduction of female employment in the fishing sector, as a result of decreased demand for fish during the epidemic)	These increasing trends were reversed during the epidemic: fewer institutional deliveries occurred (–240, 95% CI –293 to –187), and fewer women achieved at least one antenatal care visit (–418, 95% CI –535 to –300) or at least three antenatal care visits (–363, 95% CI –485 to –242) per month (p<0·0001 for all). Compared with the negative trend during the outbreak, the change in trend during the post-outbreak period showed that 173 more women per month (95% CI 51–294; p=0·0074) had at least one antenatal care visit, 257 more (95% CI 117–398; p=0·0010) had at least three antenatal care visits and 149 more (95% CI 91–206; p<0·0001) had institutional deliveries.
Relevance and importance of the paper for our own systematic review	☐ Key paper for the review
Other comments or issues to be noted about this paper	

Author(s) and date:	Mateusz M Plucinski, Timothée Guilavogui, Sidibe Sidikiba, Nouman Diakité, Souleymane Diakité, Mohamed Dioubaté, Ibrahima Bah, Ian Hennessee, Jessica K Butts, Eric S Halsey, Peter D McElroy, S Patrick Kachur, Jamila Aboulhab, Richard James, Moussa Keita
Year	2015
Title of the paper:	Effect of the Ebola-virus-disease epidemic on malaria case management in Guinea, 2014: a cross-sectional survey of health facilities
Study design	cross-sectional survey of health facilities
Setting	health facilities
PS	Not specific
Study location	Guinea
Quality appraisal according to the relevant Johanna Briggs Institute tool (please cross out or delete accordingly)	☐ Seek further information
Epidemic of reference:	Ebola
Research methods employed (e.g. qualitative interviews; modelling; surveys; systematic review)	quantitaive surveillance; qualitative interviews
Specific lockdown measure investigated in the study (e.g. generic quarantine or unspecified lockdown measure; curfew; stay at home policy; travel restrictions; school closures; market and trade shutdowns; social distancing)	generic quarantine or unspecified lockdown measure
Broad area of impact explored in the study (please cross out or delete as appropriate)	Disruption of routine services for mother and child services;
Evidence of impact to the general population from lockdown measures (e.g. 10% decrease of disposable income before and after the peak of the epidemic; 30% reduction of hospital visits for maternal services)	The reduction in the delivery of malaria care because of the Ebola-virus-disease epidemic threatens malaria control in Guinea. Untreated and inappropriately treated malaria cases lead to excess malaria mortality and more fever cases in the community, impeding the Ebola-virus-disease response; Nationwide, the Ebola-virus-disease epidemic was estimated to have resulted in 74 000 (71 000–77 000) fewer malaria cases seen at health facilities in 2014.
Specific evidence of impact on children (e.g. 30% increase of weight during two weeks of implementation of stay at home policy; or: 'calculated 365,000 extra malaria deaths calculated as a consequence of disruption of bed nets distribution)	We noted substantial reductions in all-cause outpatient visits (by 23 103 [11%] of 214 899), cases of fever (by 20249 [15%] of 131 330), and patients treated with oral (by 22 655 [24%] of 94 785) and injectable (by 5219 [30%] of 17 684) antimalarial drugs in surveyed health facilities.; . In Ebola-affected prefectures, 73 of 98 interviewed community<br>health workers were operational (74%, 95% CI 65–83) and 35 of 73 were actively treating malaria cases (48%, 36–60)<br>compared with 106 of 112 (95%, 89–98) and 102 of 106 (96%, 91–99), respectively, in Ebola-unaff ected prefectures.
Specific evidence of impact on women (e.g. 20% reduction of female employment in the fishing sector, as a result of decreased demand for fish during the epidemic)	We noted substantial reductions in all-cause outpatient visits (by 23 103 [11%] of 214 899), cases of fever (by 20249 [15%] of 131 330), and patients treated with oral (by 22 655 [24%] of 94 785) and injectable (by 5219 [30%] of 17 684) antimalarial drugs in surveyed health facilities.; . In Ebola-affected prefectures, 73 of 98 interviewed community<br>health workers were operational (74%, 95% CI 65–83) and 35 of 73 were actively treating malaria cases (48%, 36–60)<br>compared with 106 of 112 (95%, 89–98) and 102 of 106 (96%, 91–99), respectively, in Ebola-unaff ected prefectures.
Relevance and importance of the paper for our own systematic review	☐ Paper of some importance for some sections of the review
Other comments or issues to be noted about this paper	Not specific to Women and Child and therfore the imact was dulicated both in women and children

Author(s) and date:	A. S. Parpia, M. L. Ndeffo-Mbah, N. S. Wenzel and A. P. Galvani
Year	2016
Title of the paper:	Effects of Response to 2014-2015 Ebola Outbreak on Deaths from Malaria, HIV/AIDS, and Tuberculosis, West Africa
Study design	
Setting	NA
PS	Not specific
Study location	West Africa (Guinea, Liberia, and Sierra Leone)
Quality appraisal according to the relevant Johanna Briggs Institute tool (please cross out or delete accordingly)	☐ Seek further information
Epidemic of reference:	Ebola
Research methods employed (e.g. qualitative interviews; modelling; surveys; systematic review)	Modelling
Specific lockdown measure investigated in the study (e.g. generic quarantine or unspecified lockdown measure; curfew; stay at home policy; travel restrictions; school closures; market and trade shutdowns; social distancing)	generic quarantine or unspecified lockdown measure
Broad area of impact explored in the study (please cross out or delete as appropriate)	Disruption of routine services for mother and child services;
Evidence of impact to the general population from lockdown measures (e.g. 10% decrease of disposable income before and after the peak of the epidemic; 30% reduction of hospital visits for maternal services)	We estimated that a 50% reduction in access to healthcare services during the Ebola outbreak exacerbated malaria, HIV/AIDS, and tuberculosis mortality rates by additional death counts of 6,269 (2,564–12,407) in Guinea; 1,535 (522–2,8780) in Liberia; and 2,819 (844–4,844) in Sierra Leone.
Specific evidence of impact on children (e.g. 30% increase of weight during two weeks of implementation of stay at home policy; or: 'calculated 365,000 extra malaria deaths calculated as a consequence of disruption of bed nets distribution)	N/A
Specific evidence of impact on women (e.g. 20% reduction of female employment in the fishing sector, as a result of decreased demand for fish during the epidemic)	N/A
Relevance and importance of the paper for our own systematic review	☐ Paper of some importance for some sections of the review
Other comments or issues to be noted about this paper	Not specific to Women and Child

Author(s) and date:	J. Ly, V. Sathananthan, T. Griffiths, Z. Kanjee, A. Kenny, N. Gordon, G. Basu, D. Battistoli, L. Dorr, B. Lorenzen, D. R. Thomson, A. Waters, U. G. Moore, R. Roberts, W. L. Smith, M. J. Siedner and J. D. Kraemer
Year	2016
Title of the paper:	Facility-Based Delivery during the Ebola Virus Disease Epidemic in Rural Liberia: Analysis from a Cross-Sectional, Population-Based Household Survey
Study design	a Cross-Sectional, Population-Based Household Survey
Setting	Facility-Based Delivery
PS	Women
Study location	
Quality appraisal according to the relevant Johanna Briggs Institute tool (please cross out or delete accordingly)	☐ Seek further information
Epidemic of reference:	Ebola
Research methods employed (e.g. qualitative interviews; modelling; surveys; systematic review)	
Specific lockdown measure investigated in the study (e.g. generic quarantine or unspecified lockdown measure; curfew; stay at home policy; travel restrictions; school closures; market and trade shutdowns; social distancing)	generic quarantine or unspecified lockdown measure
Broad area of impact explored in the study (please cross out or delete as appropriate)	Disruption of routine services for mother and child services;
Evidence of impact to the general population from lockdown measures (e.g. 10% decrease of disposable income before and after the peak of the epidemic; 30% reduction of hospital visits for maternal services)	We detected a 30% decreased odds of FBD after the start of EVD in a rural Liberian county with relatively few cases
Specific evidence of impact on children (e.g. 30% increase of weight during two weeks of implementation of stay at home policy; or: 'calculated 365,000 extra malaria deaths calculated as a consequence of disruption of bed nets distribution)	N/A
Specific evidence of impact on women (e.g. 20% reduction of female employment in the fishing sector, as a result of decreased demand for fish during the epidemic)	The odds of facility-based delivery were 41% lower among women who reported a belief that Ebola was or may be transmitted in health facilities, but not significantly lower among women who reported believing that Ebola was not transmitted in health facilities.
Relevance and importance of the paper for our own systematic review	☐ Paper of some importance for some sections of the review
Other comments or issues to be noted about this paper	

Author(s) and date:	Y. W. Hung, M. R. Law, L. Cheng, S. Abramowitz, L. Alcayna-Stevens, G. Lurton, S. M. Mayaka, R. Olekhnovitch, G. Kyomba, H. Ruton, S. Y. Ramazani and K. A. Grepin
Year	2020
Title of the paper:	Impact of a free care policy on the utilisation of health services during an Ebola outbreak in the Democratic Republic of Congo: an interrupted time-series analysis
Study design	quasi-experimental timeseries analysis
Setting	health facilities
PS	Woman & children
Study location	
Quality appraisal according to the relevant Johanna Briggs Institute tool (please cross out or delete accordingly)	☐ Include
Epidemic of reference:	Ebola
Research methods employed (e.g. qualitative interviews; modelling; surveys; systematic review)	Modelling
Specific lockdown measure investigated in the study (e.g. generic quarantine or unspecified lockdown measure; curfew; stay at home policy; travel restrictions; school closures; market and trade shutdowns; social distancing)	generic quarantine or unspecified lockdown measure
Broad area of impact explored in the study (please cross out or delete as appropriate)	Demand for healthcare / Healthcare seeking behaviour;
Evidence of impact to the general population from lockdown measures (e.g. 10% decrease of disposable income before and after the peak of the epidemic; 30% reduction of hospital visits for maternal services)	Total visits and visits for pneumonia and diarrhoea initially increased more than two-fold relative to the control areas (p<0.001), while institutional deliveries and first antenatal care increased between 20% and 50% (p<0.01). Visits for DTP, fourth antenatal care visits and postnatal care visits were not significantly affected.
Specific evidence of impact on children (e.g. 30% increase of weight during two weeks of implementation of stay at home policy; or: ‘calculated 365,000 extra malaria deaths calculated as a consequence of disruption of bed nets distribution)	visit rates increased more than twofold in EVD and FCP health zones (incidence rate ratio (IRR): 2.7, 95%CI: 2.2–3.2, p<0.001) and FCP-only health zones (IRR: 2.7, 95%CI: 2.2–3.1, p<0.001).Following the end of the FCP, the level of total clinic visits dropped by 21% in EVD and FCP health zones (95%CI: 0.65–0.96, p=0.02) and by 18% in FCP-only health zones (95%CI: 0.68–1.0, p=0.05) compared to the FCP period, with no significant change in trend after the FCP ended (EVD and FCP: IRR: 1.0, 95%CI: 0.97–1.1, p=0.26; FCP only: IRR: 1.1, 95%CI: 0.98–1.2, p=0.12);Compared to facilities in control health zones, clinic visits for pneumonia doubled at the beginning of FCP in EVD and FCP health zones (IRR: 2.1, 95%CI: 1.5–2.9, p<0.001).
Specific evidence of impact on women (e.g. 20% reduction of female employment in the fishing sector, as a result of decreased demand for fish during the epidemic)	Maternal Health: Compared to control zones, deliveries at clinics increased in all of the free care policy (FCP) health zones immediately following the implementation of the policy (EVD and FCP: IRR: 1.3, 95% CI: 1.1–1.5, p=0.004; FCP-only: IRR: 1.2, 95% CI: 1.1–1.4, p=0.01), but the increase did not continue over the whole implementation period (EVD and FCP: IRR: 1.0, 95% CI: 0.96–1.1, p=0.83; FCP only: IRR: 0.99, 95% CI: 0.94–1.1, p=0.75).
Relevance and importance of the paper for our own systematic review	☐ Paper of some importance for some sections of the review
Other comments or issues to be noted about this paper	

Author(s) and date:	N. Sharma and H. Vaish
Year	2020
Title of the paper:	Impact of COVID-19 on mental health and physical load on women professionals: an online cross-sectional survey
Study design	cross-sectional survey
Setting	
PS	Women
Study location	Congo
Quality appraisal according to the relevant Johanna Briggs Institute tool (please cross out or delete accordingly)	☐ Exclude
Epidemic of reference:	Covid-19
Research methods employed (e.g. qualitative interviews; modelling; surveys; systematic review)	Survey
Specific lockdown measure investigated in the study (e.g. generic quarantine or unspecified lockdown measure; curfew; stay at home policy; travel restrictions; school closures; market and trade shutdowns; social distancing)	generic quarantine or unspecified lockdown measure
Broad area of impact explored in the study (please cross out or delete as appropriate)	Mental Health
Evidence of impact to the general population from lockdown measures (e.g. 10% decrease of disposable income before and after the peak of the epidemic; 30% reduction of hospital visits for maternal services)	Mental health was moderately and severely affected in 27.5% and 27% of participants respectively. 34.3% experienced great increase in physical load due to house hold chores during lockdown. 45.81% reported pain in neck and back region with 36.31% participants reported strain in their eyes sometimes. 15.08% and 8.37% had a tendency to over react in the present situation often and always respectively.
Specific evidence of impact on children (e.g. 30% increase of weight during two weeks of implementation of stay at home policy; or: 'calculated 365,000 extra malaria deaths calculated as a consequence of disruption of bed nets distribution)	
Specific evidence of impact on women (e.g. 20% reduction of female employment in the fishing sector, as a result of decreased demand for fish during the epidemic)	Mental health was moderately and severely affected in 27.5% and 27% of participants respectively. 34.3% experienced great increase in physical load due to house hold chores during lockdown. 45.81% reported pain in neck and back region with 36.31% participants reported strain in their eyes sometimes. 15.08% and 8.37% had a tendency to over react in the present situation often and always respectively.
Relevance and importance of the paper for our own systematic review	☐ Secondary paper
Other comments or issues to be noted about this paper	

Author(s) and date:	M. Ali, G. U. Ahsan, R. Khan, H. R. Khan and A. Hossain
Year	2020
Title of the paper:	Immediate impact of stay-at-home orders to control COVID-19 transmission on mental well-being in Bangladeshi adults: Patterns, Explanations, and future directions
Study design	cross-sectional study
Setting	
PS	Women & Men adults
Study location	Bangladeshi
Quality appraisal according to the relevant Johanna Briggs Institute tool (please cross out or delete accordingly)	☐ Include
Epidemic of reference:	Covid-19
Research methods employed (e.g. qualitative interviews; modelling; surveys; systematic review)	Survey
Specific lockdown measure investigated in the study (e.g. generic quarantine or unspecified lockdown measure; curfew; stay at home policy; travel restrictions; school closures; market and trade shutdowns; social distancing)	stay at home policy
Broad area of impact explored in the study (please cross out or delete as appropriate)	Mental Health;
Evidence of impact to the general population from lockdown measures (e.g. 10% decrease of disposable income before and after the peak of the epidemic; 30% reduction of hospital visits for maternal services)	The overall mean score for well-being was 42.4, indicating that 51.9% of adults sufered from poor mental health; Te participants who were involved in business had worse mental health than government employees (decreased by 5.87 units, p=0.01), health care workers (by 4.98, p≤0.001), and employees of private companies (by 3.31, p=0.02).
Specific evidence of impact on children (e.g. 30% increase of weight during two weeks of implementation of stay at home policy; or: 'calculated 365,000 extra malaria deaths calculated as a consequence of disruption of bed nets distribution)	
Specific evidence of impact on women (e.g. 20% reduction of female employment in the fishing sector, as a result of decreased demand for fish during the epidemic)	57% of females were depressed; Depression seemed to be heavily skewed towards women, raising agonizing concerns. For instance, 57.2% of female participants were in poor mental health (i.e. WEMWBS score≤42), whereas for males it was at 48.9%.; Interestingly, the unmarried females appear to have higher well-being scores than the married women (by 3.31, p=0.01).
Relevance and importance of the paper for our own systematic review	☐ key paper for the review
Other comments or issues to be noted about this paper	

Author(s) and date:	X. J. Sun, T. T. Samba, J. Y. Yao, W. W. Yin, L. Xiao, F. Q. Liu, X. Q. Liu, J. K. Zhou, Z. Q. Kou, H. W. Fan, H. Zhang, A. Williams, P. M. Lansana and Z. D. Yin
Year	2017
Title of the paper:	Impact of the Ebola outbreak on routine immunization in western area, Sierra Leone - a field survey from an Ebola epidemic area
Study design	cross-sectional study
Setting	Children
PS	Sierra Leone
Study location	
Quality appraisal according to the relevant Johanna Briggs Institute tool (please cross out or delete accordingly)	☐ Include
Epidemic of reference:	Ebola
Research methods employed (e.g. qualitative interviews; modelling; surveys; systematic review)	qualitative interviews
Specific lockdown measure investigated in the study (e.g. generic quarantine or unspecified lockdown measure; curfew; stay at home policy; travel restrictions; school closures; market and trade shutdowns; social distancing)	generic quarantine or unspecified lockdown measure
Broad area of impact explored in the study (please cross out or delete as appropriate)	Disruption of routine services for mother and child services;
Evidence of impact to the general population from lockdown measures (e.g. 10% decrease of disposable income before and after the peak of the epidemic; 30% reduction of hospital visits for maternal services)	Measles Vaccine (MV) coverage among age-eligible children was 71.3% (95% confidence interval [CI]: 62.1% - 80.4%) and 45.7% (95% CI: 29.2% - 62.2%) before and during the outbreak of EVD, respectively, and was 56.8% (95% CI: 40.8% - 72.7%) after the campaign. Pentavalent vaccine (Pentavalent3) coverage among age-eligible children was 79.8% (95% CI: 72.6% - 87.0%) and 40.0% (95% CI: 22.5% - 57.5%) before and during the outbreak of EVD, and was 56.4% (95% CI: 39.1% - 73.4%) after the campaign.
Specific evidence of impact on children (e.g. 30% increase of weight during two weeks of implementation of stay at home policy; or: 'calculated 365,000 extra malaria deaths calculated as a consequence of disruption of bed nets distribution)	In phase 1 (before EVD), the age-eligible, MV-vaccinated rate was 71.3% (95% CI: 62.1% - 80.4%) while in phase 2 (during EVD), the MV-vaccinated rate was 45.7% (95% CI: 29.2% - 62.2%). Therefore, age-eligible coverage during the EVD outbreak was 25.6 percentage points (95% CI: -44.2 to −7.0 percentage points) lower than before the EVD outbreak (χ 2 = 7.3, P < 0.01).; In phase 1 (before EVD), the age-eligible Pentavalent3- vaccinated coverage was 79.8% (95% CI: 72.6% - 87.0%), while in phase 2 (during EVD), Pentavalent3-vaccinated coverage was 40.0% (95% CI: 22.5% - 57.5%). Therefore, age-eligible coverage during the EVD outbreak was 39.8 percentage points (95% CI: -57.8 to −21.8 percentage points) lower than before the EVD outbreak (χ 2 = 18.8, P < 0.01).
Specific evidence of impact on women (e.g. 20% reduction of female employment in the fishing sector, as a result of decreased demand for fish during the epidemic)	Following the MCHW campaign, MV coverage increased to 56.8% (95% CI: 40.8% - 72.7%), but was not statistically different than MV coverage during the EVD outbreak (χ 2 = 0.9, P > 0.05). Following the MCHW campaign, Pentavalent3 coverage increased to 56.3% (95% CI: 39.1% - 73.4%), but was not statistically different than coverage during the EVD outbreak (χ 2 = 1.6, P > 0.05)
Relevance and importance of the paper for our own systematic review	☐ Paper of some importance for some sections of the review
Other comments or issues to be noted about this paper	

Author(s) and date:	G. Quaglio, F. Tognon, L. Finos, D. Bome, S. Sesay, A. Kebbie, F. Di Gennaro, B. S. Camara, C. Marotta, V. Pisani, Z. Bangura, D. Pizzol, A. Saracino, W. Mazzucco, S. Jones and G. Putoto
Year	2019
Title of the paper:	Impact of Ebola outbreak on reproductive health services in a rural district of Sierra Leone: a prospective observational study
Study design	a prospective observational study
Setting	community health facilities and hospital
PS	Woman & children
Study location	Sierra Leone
Quality appraisal according to the relevant Johanna Briggs Institute tool (please cross out or delete accordingly)	☐ Include
Epidemic of reference:	Ebola
Research methods employed (e.g. qualitative interviews; modelling; surveys; systematic review)	survey
Specific lockdown measure investigated in the study (e.g. generic quarantine or unspecified lockdown measure; curfew; stay at home policy; travel restrictions; school closures; market and trade shutdowns; social distancing)	generic quarantine or unspecified lockdown measure
Broad area of impact explored in the study (please cross out or delete as appropriate)	Disruption of routine services for mother and child services;
Evidence of impact to the general population from lockdown measures (e.g. 10% decrease of disposable income before and after the peak of the epidemic; 30% reduction of hospital visits for maternal services)	At hospital level, there is a significant difference between trends Ebola versus pre-Ebola for maternal admissions (7, 95%CI 4 to 11, p<0.001). MDOCs (4, 95%CI 1 to 7, p=0.006) and institutional deliveries (4, 95%CI 2 to 6, p=0.001).There is also a negative trend in the transition from Ebola to post-Ebola for maternal admissions (−7, 95%CI −10 to −4, p<0.001), MDOCs (−4, 95%CI −7 to −1, p=0.009) and institutional deliveries (−3, 95%CI −5 to −1, p=0.001); The differences between trends pre-Ebola versus post-Ebola are only significant for paediatric admissions (3, 95%CI 0 to 5, p=0.035).; The differences between trends pre-Ebola versus post-Ebola show a negative difference for institutional deliveries (−7, 95%CI −10 to −4, p<0.001), ANC 1 (−6, 95%CI −10 to −3, p<0.001), ANC 4 (−8, 95%CI −11 to −5, p<0.001) and family planning (−85, 95%CI −119 to −51, p<0.001).
Specific evidence of impact on children (e.g. 30% increase of weight during two weeks of implementation of stay at home policy; or: 'calculated 365,000 extra malaria deaths calculated as a consequence of disruption of bed nets distribution)	
Specific evidence of impact on women (e.g. 20% reduction of female employment in the fishing sector, as a result of decreased demand for fish during the epidemic)	At hospital level, there is a significant difference between trends Ebola versus pre-Ebola for maternal admissions (7, 95%CI 4 to 11, p<0.001). MDOCs (4, 95%CI 1 to 7, p=0.006) and institutional deliveries (4, 95%CI 2 to 6, p=0.001).There is also a negative trend in the transition from Ebola to post-Ebola for maternal admissions (−7, 95%CI −10 to −4, p<0.001), MDOCs (−4, 95%CI −7 to −1, p=0.009) and institutional deliveries (−3, 95%CI −5 to −1, p=0.001); The differences between trends pre-Ebola versus post-Ebola are only significant for paediatric admissions (3, 95%CI 0 to 5, p=0.035).; The differences between trends pre-Ebola versus post-Ebola show a negative difference for institutional deliveries (−7, 95%CI −10 to −4, p<0.001), ANC 1 (−6, 95%CI −10 to −3, p<0.001), ANC 4 (−8, 95%CI −11 to −5, p<0.001) and family planning (−85, 95%CI −119 to −51, p<0.001).
Relevance and importance of the paper for our own systematic review	☐ key paper for the review
Other comments or issues to be noted about this paper	

Author(s) and date:	S. Yeasmin, R. Banik, S. Hossain, M. N. Hossain, R. Mahumud, N. Salma and M. M. Hossain
Year	2020
Title of the paper:	Impact of COVID-19 pandemic on the mental health of children in Bangladesh: A cross-sectional study
Study design	A cross-sectional study
Setting	
PS	Children
Study location	Bangladeshi
Quality appraisal according to the relevant Johanna Briggs Institute tool (please cross out or delete accordingly)	☐ Include
Epidemic of reference:	Covid-19
Research methods employed (e.g. qualitative interviews; modelling; surveys; systematic review)	Survey
Specific lockdown measure investigated in the study (e.g. generic quarantine or unspecified lockdown measure; curfew; stay at home policy; travel restrictions; school closures; market and trade shutdowns; social distancing)	generic quarantine or unspecified lockdown measure
Broad area of impact explored in the study (please cross out or delete as appropriate)	Mental Health
Evidence of impact to the general population from lockdown measures (e.g. 10% decrease of disposable income before and after the peak of the epidemic; 30% reduction of hospital visits for maternal services)	Children were classified into four groups where 43% of child had subthreshold mental disturbances (mean Major Depressive Disorder (MDD)-10; 2.8), 30.5% had mild (mean MDD-10; 8.9), 19.3% suffered moderately (mean MDD-10; 15.9), and 7.2% of child suffered from severe disturbances (mean MDD-10; 25.2)
Specific evidence of impact on children (e.g. 30% increase of weight during two weeks of implementation of stay at home policy; or: 'calculated 365,000 extra malaria deaths calculated as a consequence of disruption of bed nets distribution)	Results depict that 43% of child had subthreshold mental health disturbances (mean depression: 2.8, anxiety: 2, and sleeping disorder: 1), 30.5% had mild disturbances (mean depression: 8.9, anxiety: 4.9, and sleeping disorder: 3), 19.3% suffered from moderate disturbances (mean depression:15.9, anxiety: 9.2, and sleeping: 6), and 7.2% suffered from severe disturbances (mean depression: 25.2, anxiety: 13.4, and sleeping disorder: 8).
Specific evidence of impact on women (e.g. 20% reduction of female employment in the fishing sector, as a result of decreased demand for fish during the epidemic)	
Relevance and importance of the paper for our own systematic review	☐ Paper of some importance for some sections of the review
Other comments or issues to be noted about this paper	

Author(s) and date:	N. Emmanuel Awucha, O. Chinelo Janefrances, A. Chima Meshach, J. Chiamaka Henrietta, A. Ibilolia Daniel and N. Esther Chidiebere
Year	2020
Title of the paper:	Impact of the COVID-19 Pandemic on Consumers' Access to Essential Medicines in Nigeria
Study design	A cross-sectional study
Setting	
PS	Women and Men
Study location	Nigeria
Quality appraisal according to the relevant Johanna Briggs Institute tool (please cross out or delete accordingly)	☐ Include
Epidemic of reference:	Covid-19
Research methods employed (e.g. qualitative interviews; modelling; surveys; systematic review)	Survey
Specific lockdown measure investigated in the study (e.g. generic quarantine or unspecified lockdown measure; curfew; stay at home policy; travel restrictions; school closures; market and trade shutdowns; social distancing)	generic quarantine or unspecified lockdown measure
Broad area of impact explored in the study (please cross out or delete as appropriate)	ease of access to essential medicines
Evidence of impact to the general population from lockdown measures (e.g. 10% decrease of disposable income before and after the peak of the epidemic; 30% reduction of hospital visits for maternal services)	The results showed that 35.2% of the respondents managing chronic illnesses had difficulties accessing essential medicines during the COVID-19 lockdown, with 84.0% experiencing deteriorating chronic health conditions in the light of difficulty in accessing their medicines. The proportion of respondents who sourced for orthodox medicines before COVID-19 lockdown (98.4%) was significantly ($P < 0.05$) higher than that of those who sourced for the same during the lockdown (89.0%). Increase in cost of medicines was observed by 77.7% of participants, with 73.9% of respondents living with chronic illness affirming that their income was negatively affected by the pandemic.
Specific evidence of impact on children (e.g. 30% increase of weight during two weeks of implementation of stay at home policy; or: 'calculated 365,000 extra malaria deaths calculated as a consequence of disruption of bed nets distribution)	
Specific evidence of impact on women (e.g. 20% reduction of female employment in the fishing sector, as a result of decreased demand for fish during the epidemic)	For respondents living with chronic conditions, there was an increase in the proportion of those facing difficulties to essential medicine access, from 10.6% before the lockdown to 35.2% during the lockdown; 84.0% experienced a worsening of the chronic conditions, which was significant; Overall, those who had acute illnesses during the lockdown had 72.0% essential medicines accessibility, whereas those with chronic conditions had 65.0% accessibility; 77.3% of the respondents observed an increase in medicine costs ; 84.0% of our respondents experienced deteriorating chronic health conditions in the light of difficulty in accessing essential medicines.
Relevance and importance of the paper for our own systematic review	☐ Paper of some importance for some sections of the review
Other comments or issues to be noted about this paper	No pecific, thus we assume women as part of the results

Author(s) and date:	P. A. McQuilkin, K. Udhayashankar, M. Niescierenko and L. Maranda
Year	2017
Title of the paper:	Health-Care Access during the Ebola Virus Epidemic in Liberia
Study design	Community-based survey
Setting	
PS	Women and Men
Study location	Liberia
Quality appraisal according to the relevant Johanna Briggs Institute tool (please cross out or delete accordingly)	☐ Seek further information
Epidemic of reference:	Ebola
Research methods employed (e.g. qualitative interviews; modelling; surveys; systematic review)	Survey
Specific lockdown measure investigated in the study (e.g. generic quarantine or unspecified lockdown measure; curfew; stay at home policy; travel restrictions; school closures; market and trade shutdowns; social distancing)	generic quarantine or unspecified lockdown measure
Broad area of impact explored in the study (please cross out or delete as appropriate)	Health-Care Access
Evidence of impact to the general population from lockdown measures (e.g. 10% decrease of disposable income before and after the peak of the epidemic; 30% reduction of hospital visits for maternal services)	more than half (67%) of urban respondents and 46% of rural respondents stated that it was very difficult or impossible to access health care during the epidemic.; For those who sought care at government hospitals and were unable to receive it, the major barriers were closure of facilities (50%), HCWs refusing to see patients (42%), and fear of referral to EVD treatment units (2%).
Specific evidence of impact on children (e.g. 30% increase of weight during two weeks of implementation of stay at home policy; or: 'calculated 365,000 extra malaria deaths calculated as a consequence of disruption of bed nets distribution)	
Specific evidence of impact on women (e.g. 20% reduction of female employment in the fishing sector, as a result of decreased demand for fish during the epidemic)	In urban areas, only 20–30% of patients seeking care during the epidemic received care, and in rural areas, only 70–80% of those seeking care were able to access it. ; In urban areas, only approximately 25% of patients who sought care received it. Care for pediatric patients (20.7%) and prenatal (22.9%) and obstetric care (22.3%) was especially challenging in urban areas.
Relevance and importance of the paper for our own systematic review	☐ Secondary paper
Other comments or issues to be noted about this paper	

Author(s) and date:	S. Chandir, D. A. Siddiqi, M. Mehmood, H. Setayesh, M. Siddique, A. Mirza, R. Soundardjee, V. K. Dharma, M. T. Shah, S. Abdullah, M. A. Akhter, A. Ali Khan and A. J. Khan
Year	2020
Title of the paper:	Impact of COVID-19 pandemic response on uptake of routine immunizations in Sindh, Pakistan: An analysis of provincial electronic immunization registry data
Study design	A cross-sectional study
Setting	
PS	Children
Study location	Pakistan
Quality appraisal according to the relevant Johanna Briggs Institute tool (please cross out or delete accordingly)	☐ Include
Epidemic of reference:	Covid-19
Research methods employed (e.g. qualitative interviews; modelling; surveys; systematic review)	Survey
Specific lockdown measure investigated in the study (e.g. generic quarantine or unspecified lockdown measure; curfew; stay at home policy; travel restrictions; school closures; market and trade shutdowns; social distancing)	generic quarantine or unspecified lockdown measure
Broad area of impact explored in the study (please cross out or delete as appropriate)	Disruption of routine services for mother and child services;
Evidence of impact to the general population from lockdown measures (e.g. 10% decrease of disposable income before and after the peak of the epidemic; 30% reduction of hospital visits for maternal services)	There was a 52.5% decline in the daily average total number of vaccinations administered during lockdown compared to baseline. The highest decline was seen for Bacille Calmette Guérin (BCG) (40.6% (958/2360) immunization at fixed sites. Around 8438 children/day were missing immunization during the lockdown
Specific evidence of impact on children (e.g. 30% increase of weight during two weeks of implementation of stay at home policy; or: ‘calculated 365,000 extra malaria deaths calculated as a consequence of disruption of bed nets distribution)	Pentavalent-3 (penta-3) immunization rates were higher in infants born in hospitals (RR: 1.09; 95% CI: 1.04–1.15) and those with mothers having higher education (RR: 1.19–1.50; 95% CI: 1.13–1.65). Likelihood of penta-3 immunization was reduced by 5% for each week of delayed enrollment into the immunization program.; Children immunized during lockdown, as compared to baseline, had a higher proportion of facility-based births, more caregivers had provided contact numbers (33.2% vs. 27.4%, p-value 0.01; 95% CI: 5.47–6.13), and the mean age at BCG vaccination was lower (4.3 vs. 6.3 weeks, pvalue 0.01; 95% CI: 1.93–2.07). Additionally, more children with higher maternal education, (in the category 9 years and above) were enrolled during lockdown as compared to baseline (pvalue 0.01; 95% CI: 1.01–1.45). Daily average vaccinator attendance was 7.4% (p-value < 0.0001; 95% CI: 5.29–9.51) lower during the lockdown compared to baseline (78.8% (79,252/100,600 person days) vs. 86.2% (312,386/362,551 person days) respectively).
Specific evidence of impact on women (e.g. 20% reduction of female employment in the fishing sector, as a result of decreased demand for fish during the epidemic)	The reduction in mean proportion of vaccinators who attended work during the lockdown compared with baseline was higher for female vaccinators (13.7%; 95% CI: 12.9–14.5) vs male vaccinators (6.3%; 95% CI: 6.0–6.6).
Relevance and importance of the paper for our own systematic review	☐ Paper of some importance for some sections of the review
Other comments or issues to be noted about this paper	

Author(s) and date:	Mohapatra S
Year	2020
Title of the paper:	Gender differentiated economic responses to crises in developing countries: insights for COVID-19 recovery policies
Study design	NA
Setting	NA
PS	Women and Men
Study location	NA
Quality appraisal according to the relevant Johanna Briggs Institute tool (please cross out or delete accordingly)	☐ Exclude
Epidemic of reference:	Covid-19
Research methods employed (e.g. qualitative interviews; modelling; surveys; systematic review)	Modelling
Specific lockdown measure investigated in the study (e.g. generic quarantine or unspecified lockdown measure; curfew; stay at home policy; travel restrictions; school closures; market and trade shutdowns; social distancing)	NA
Broad area of impact explored in the study (please cross out or delete as appropriate)	NA
Evidence of impact to the general population from lockdown measures (e.g. 10% decrease of disposable income before and after the peak of the epidemic; 30% reduction of hospital visits for maternal services)	An economic growth shock creates a sharp decline in female employment by 3 percentage points within the first 5 years after the shock (Fig. 6a). The magnitudes of the employment declines are large. For instance, applied to the Indian context, the total number of female workers according to the 2011 census is about 150 million. A 3 percentage point drop implies that, following a GDP contraction, there are 4.5 million jobs lost 5 years after the shock over the previous year.
Specific evidence of impact on children (e.g. 30% increase of weight during two weeks of implementation of stay at home policy; or: 'calculated 365,000 extra malaria deaths calculated as a consequence of disruption of bed nets distribution)	
Specific evidence of impact on women (e.g. 20% reduction of female employment in the fishing sector, as a result of decreased demand for fish during the epidemic)	
Relevance and importance of the paper for our own systematic review	☐ Exclude
Other comments or issues to be noted about this paper	

Author(s) and date:	J. Juan, M. M. Gil, Z. Rong, Y. Zhang, H. Yang and L. C. Poon
Year	2020
Title of the paper:	Effect of coronavirus disease 2019 (COVID-19) on maternal, perinatal and neonatal outcome: systematic review
Study design	systematic review
Setting	
PS	Women
Study location	
Quality appraisal according to the relevant Johanna Briggs Institute tool (please cross out or delete accordingly)	☐ Include
Epidemic of reference:	Covid-19
Research methods employed (e.g. qualitative interviews; modelling; surveys; systematic review)	Sstematic review
Specific lockdown measure investigated in the study (e.g. generic quarantine or unspecified lockdown measure; curfew; stay at home policy; travel restrictions; school closures; market and trade shutdowns; social distancing)	generic quarantine or unspecified lockdown measure
Broad area of impact explored in the study (please cross out or delete as appropriate)	The effect of COVID-19 on Women Health
Evidence of impact to the general population from lockdown measures (e.g. 10% decrease of disposable income before and after the peak of the epidemic; 30% reduction of hospital visits for maternal services)	Women: The mostcommon symptoms at presentation were fever, cough,dyspnea/shortness of breath, fatigue and myalgia. Therate of severe pneumonia reported amongst the case seriesranged from 0% to 14%, with the majority of the casesrequiring admission to the intensive care unit. Almostall cases from the case series had positive computedtomography chest findings. All six and 22 cases thathad nucleic-acid testing in vaginal mucus and breastmilk samples, respectively, were negative for severe acuterespiratory syndrome coronavirus 2 (SARS-CoV-2). Onlyfour cases of spontaneous miscarriage or termination werereported. I
Specific evidence of impact on children (e.g. 30% increase of weight during two weeks of implementation of stay at home policy; or: 'calculated 365,000 extra malaria deaths calculated as a consequence of disruption of bed nets distribution)	
Specific evidence of impact on women (e.g. 20% reduction of female employment in the fishing sector, as a result of decreased demand for fish during the epidemic)	In the consecutive case series, 219/295 womenhad delivered at the time of reporting and 78% of themhad Cesarean section. The gestational age at deliveryranged from 28 to 41 weeks. Apgar scores at both 1 and5 min ranged from 7 to 10. Only eight neonates hadbirth weight < 2500 g and nearly one-third of neonateswere transferred to the neonatal intensive care unit. Therewas one case of neonatal asphyxia and death. In 155neonates that had nucleic-acid testing in throat swab, all,except three cases, were negative for SARS-CoV-2. Therewere no cases of maternal death in the eight consecutivecase series. Seven maternal deaths, four intrauterine fetaldeaths (one with twin pregnancy) and two neonatal deaths(twin pregnancy) were reported in a non-consecutive caseseries of nine cases with severe COVID-19. In the casereports, two maternal deaths, one neonatal death and twocases of neonatal SARS-CoV-2 infection were reported
Relevance and importance of the paper for our own systematic review	☐ Paper of some importance for some sections of the review
Other comments or issues to be noted about this paper	

Author(s) and date:	K. Dutta, R. Mukherjee, D. Sen and S. Sahu
Year	2020
Title of the paper:	Effect of COVID-19 lockdown on sleep behavior and screen exposure time: an observational study among Indian school children
Study design	Not available
Setting	Not available
PS	Not available
Study location	Not available
Quality appraisal according to the relevant Johanna Briggs Institute tool (please cross out or delete accordingly)	Not available
Epidemic of reference:	Not available
Research methods employed (e.g. qualitative interviews; modelling; surveys; systematic review)	Not available
Specific lockdown measure investigated in the study (e.g. generic quarantine or unspecified lockdown measure; curfew; stay at home policy; travel restrictions; school closures; market and trade shutdowns; social distancing)	Not available
Broad area of impact explored in the study (please cross out or delete as appropriate)	Not available
Evidence of impact to the general population from lockdown measures (e.g. 10% decrease of disposable income before and after the peak of the epidemic; 30% reduction of hospital visits for maternal services)	Not available
Specific evidence of impact on children (e.g. 30% increase of weight during two weeks of implementation of stay at home policy; or: 'calculated 365,000 extra malaria deaths calculated as a consequence of disruption of bed nets distribution)	Not available
Specific evidence of impact on women (e.g. 20% reduction of female employment in the fishing sector, as a result of decreased demand for fish during the epidemic)	Not available
Relevance and importance of the paper for our own systematic review	Not available
Other comments or issues to be noted about this paper	This website is currently unavailable due to technical difficulties, which we are working to fix. We apologize for this downtime and hope to restore access to the site as soon as possible.

Author(s) and date:	A. Ammar, M. Brach, K. Trabelsi, H. Chtourou, O. Boukhris, L. Masmoudi, B. Bouaziz, E. Bentlage, D. How, M. Ahmed, P. Müller, N. Müller, A. Aloui, O. Hammouda, L. L. Paineiras-Domingos, A. Braakman-Jansen, C. Wrede, S. Bastoni, C. S. Pernambuco, L. Mataruna, M. Taheri, K. Irandoust, A. Khacharem, N. L. Bragazzi, K. Chamari, J. M. Glenn, N. T. Bott, F. Gargouri, L. Chaari, H. Batatia, G. M. Ali, O. Abdelkarim, M. Jarraya, K. El Abed, N. Souissi, L. Van Gemert-Pijnen, B. L. Riemann, L. Riemann, W. Moalla, J. Gómez-Raja, M. Epstein, R. Sanderman, S. V. W. Schulz, A. Jerg, R. Al-Horani, T. Mansi, M. Jmail, F. Barbosa, F. Ferreira-Santos, B. Šimuni?, R. Pišot, A. Gaggioli, S. J. Bailey, J. M. Steinacker, T. Driss, A. Hoekelmann and E.-C. Consortium
Year	2020
Title of the paper:	Effects of COVID-19 home confinement on eating behaviour and physical activity: Results of the ECLB-COVID19 international online survey
Study design	Onlie Survey
Setting	Onlie Survey
PS	Women and Men
Study location	worldiwde
Quality appraisal according to the relevant Johanna Briggs Institute tool (please cross out or delete accordingly)	☐ Include
Epidemic of reference:	Covid-19
Research methods employed (e.g. qualitative interviews; modelling; surveys; systematic review)	Survey
Specific lockdown measure investigated in the study (e.g. generic quarantine or unspecified lockdown measure; curfew; stay at home policy; travel restrictions; school closures; market and trade shutdowns; social distancing)	generic quarantine or unspecified lockdown measure
Broad area of impact explored in the study (please cross out or delete as appropriate)	home confinement on eating behaviour and physical activity:
Evidence of impact to the general population from lockdown measures (e.g. 10% decrease of disposable income before and after the peak of the epidemic; 30% reduction of hospital visits for maternal services)	The COVID-19 home confinement had a negative effect on all PA intensity levels (vigorous, moderate, walking and overall). Additionally, daily sitting time increased from 5 to 8 h per day. Food consumption and meal patterns (the type of food, eating out of control, snacks between meals, number of main meals) were more unhealthy during confinement, with only alcohol binge drinking decreasing significantly
Specific evidence of impact on children (e.g. 30% increase of weight during two weeks of implementation of stay at home policy; or: 'calculated 365,000 extra malaria deaths calculated as a consequence of disruption of bed nets distribution)	
Specific evidence of impact on women (e.g. 20% reduction of female employment in the fishing sector, as a result of decreased demand for fish during the epidemic)	The number of days/week and minutes/day of vigorous intensity PA during, compared to before, home confinement decreased by 22.7% ($t = 7.75$, $p < 0.001$, $d = 0.374$) and 33.1% ($t = 9.75$ $p < 0.001$, $d = 0.542$), respectively. Additionally, MET values of vigorous intensity PA were 36.9% lower during, compared to before, home confinement ($t = 6.68$, $p < 0.001$, $d = 0.315$).
Relevance and importance of the paper for our own systematic review	☐ secondary paper
Other comments or issues to be noted about this paper	

Author(s) and date:	G. Li, D. Tang, B. Song, C. Wang, S. Qunshan, C. Xu, H. Geng, H. Wu, X. He and Y. Cao
Year	
Title of the paper:	Impact of the COVID-19 Pandemic on Partner Relationships and Sexual and Reproductive Health: Cross-Sectional, Online Survey Study
Study design	Cross-Sectional, Online Survey Study
Setting	Not applicable
PS	Not applicable
Study location	Not applicable
Quality appraisal according to the relevant Johanna Briggs Institute tool (please cross out or delete accordingly)	☐ Exclude
Epidemic of reference:	Covid-19
Research methods employed (e.g. qualitative interviews; modelling; surveys; systematic review)	Online Survey
Specific lockdown measure investigated in the study (e.g. generic quarantine or unspecified lockdown measure; curfew; stay at home policy; travel restrictions; school closures; market and trade shutdowns; social distancing)	Not applicable
Broad area of impact explored in the study (please cross out or delete as appropriate)	Not applicable
Evidence of impact to the general population from lockdown measures (e.g. 10% decrease of disposable income before and after the peak of the epidemic; 30% reduction of hospital visits for maternal services)	Not applicable
Specific evidence of impact on children (e.g. 30% increase of weight during two weeks of implementation of stay at home policy; or: 'calculated 365,000 extra malaria deaths calculated as a consequence of disruption of bed nets distribution)	Not applicable
Specific evidence of impact on women (e.g. 20% reduction of female employment in the fishing sector, as a result of decreased demand for fish during the epidemic)	Not applicable
Relevance and importance of the paper for our own systematic review	☐ Exclude
Other comments or issues to be noted about this paper	Not from LMICs

Table S1. Data extraction form for SLR on the health impact of lockdown measures for women and children in LMICs

Title of the paper:	Access to Healthcare in a time of COVID-19: Sex Workers in Crisis in Nairobi, Kenya
Author(s) and date:	Susan Gichuna , Rahma Hassan , Teela Sanders , Rosie Campbell , Mercy Mutonyi & Peninah Mwangi
Quality appraisal according to the relevant Johanna Briggs Institute tool (please cross out or delete accordingly)	☐ Include
Epidemic of reference:	Covid-19
Research methods employed (e.g. qualitative interviews; modelling; surveys; systematic review)	Qualitative – 117 sex workers and 15 healthcare providers
Specific lockdown measure investigated in the study (e.g. generic quarantine or unspecified lockdown measure; curfew; stay at home policy; travel restrictions; school closures;	Mentioned lockdown in general terms. The paper notes that 'These include the cessation of movement, dusk to dawn curfew, stay-at-home requirements and a ban on all forms of gatherings – religious, political and social. The government enforced containment measures by deploying police to ensure adherence to the night curfew, social distancing and wearing of face masks'

market and trade shutdowns; social distancing)	
Broad area of impact explored in the study (please cross out or delete as appropriate)	36. Demand for healthcare / Healthcare seeking behaviour;<br>37. Impact on partner relationships, sexual and reproductive behaviour;<br>38. Increases in social vulnerability, poverty and impact on human development;
Evidence of impact to the general population from lockdown measures (e.g. 10% decrease of disposable income before and after the peak of the epidemic; 30% reduction of hospital visits for maternal services)	
Specific evidence of impact on children (e.g. 30% increase of weight during two weeks of implementation of stay at home policy; or: 'calculated 365,000 extra malaria deaths calculated as a consequence of disruption of bed nets distribution)	
Specific evidence of impact on women (e.g. 20% reduction of female employment in the fishing sector, as a result of decreased demand for fish during the epidemic)	Sex workers could not access healthcare due to movement restrictions and also having to conduct more business during the day (when clinics were open).<br><br>Social distancing reduced opportunities for peer support amongst sex workers, as well as making clinic visits more time consuming.<br><br>Challenges accessing ARVs and PrEP – potentially increased HIV risk<br><br>Reduced access to family planning services – noted that there would be 'corona babies' and unwanted pregnancies. Reduced supply of condoms.
Relevance and importance of the paper for our own systematic review	☐<br>☐ Paper of some importance for some sections of the review<br>☐
Other comments or issues to be noted about this paper	

Table S1. Data extraction form for SLR on the health impact of lockdown measures for women and children in LMICs

Title of the paper:	COVID-19 response needs to broaden financial inclusion to curb the rise in poverty
Author(s) and date:	Roxana Gutiérrez-Romero Mostak Ahamed
Quality appraisal according to the relevant Johanna Briggs Institute tool (please cross out or delete accordingly)	☐ Include<br><br>No JBI tool for modelling
Epidemic of reference:	Covid
Research methods employed (e.g. qualitative interviews; modelling; surveys; systematic review)	Modelling
Specific lockdown measure investigated in the study (e.g. generic quarantine or unspecified lockdown measure; curfew; stay at home policy; travel restrictions; school closures; market and trade shutdowns; social distancing)	Only second half of paper relevant<br><br>forecasts changes in the poverty headcount ratio using the $1.90, $3.20 and $5.50 dollars a day poverty lines.<br><br>Impact of lockdown implied, but only through assumptions around reductions of GDP growth (which are not solely related to lockdowns)
Broad area of impact explored in the study (please cross out or delete as appropriate)	39. Increases in social vulnerability, poverty and impact on human development;<br>40.
Evidence of impact to the general population from lockdown measures (e.g. 10% decrease of disposable income before and after the peak of the epidemic; 30% reduction of hospital visits for maternal services)	Results forecast that "globally, the percentage of people living under $1.90 a day would increase from 13.1% in 2019, to about 13.8% in 2020 and 14.5% by 2021. This represents an increase of 107.8 million people in poverty, using the $1.90 dollars a day poverty line"<br><br>"the percentage of people worldwide living under $3.20 a day would increase from 24.8% in 2019 to nearly 27% by 2021, pushing nearly 169.4 million people in poverty. in terms of the percentage of people living under $5.50 a day, poverty would increase from 40% in 2019 to nearly 43% by 2021, pushing nearly 231 million people in poverty."
Specific evidence of impact on children (e.g. 30% increase of weight during two weeks of implementation of stay at home policy; or: 'calculated 365,000 extra malaria deaths calculated as a consequence of disruption of bed nets distribution)	
Specific evidence of impact on women (e.g. 20% reduction of female employment in the	

fishing sector, as a result of decreased demand for fish during the epidemic)	
Relevance and importance of the paper for our own systematic review	☐ Secondary paper
Other comments or issues to be noted about this paper	Provides high level background on forecasted poverty increases

Table 5: Data extraction form for SLR on the health impact of lockdown measures for women and children in LMICs

Title of the paper:	Immediate impact of stay-at-home orders to control COVID-19 transmission on socioeconomic conditions, food insecurity, mental health, and intimate partner violence in Bangladeshi women and their families: an interrupted time series
Author(s) and date:	Hamadani et al (2020)
Quality appraisal according to the relevant Johanna Briggs Institute tool (please cross out or delete accordingly)	☐ Include
Epidemic of reference:	COVID-19
Research methods employed (e.g. qualitative interviews; modelling; surveys; systematic review)	Interrupted time-series analysis from a randomised control trial
Specific lockdown measure investigated in the study (e.g. generic quarantine or unspecified lockdown measure; curfew; stay at home policy; travel restrictions; school closures; market and trade shutdowns; social distancing)	Stay at home policies
Broad area of impact explored in the study (please cross out or delete as appropriate)	41. Mental Health;<br>42. Livelihood (poverty);<br>43. Other – domestic violence.
Evidence of impact to the general population from lockdown measures (e.g. 10% decrease of disposable income before and after the peak of the epidemic; 30% reduction of hospital visits for maternal services)	A reduction in work for the father or other family members was reported by 2321 (96.0%,) of the families in the sample. Median monthly income fell from US$212 at baseline to $59 during lockdown. At baseline, five (0.2%) of 2422 families were earning less than $1.90 per day, and during the lockdown this number increased to 992 (47.3%,) of 2096 ($p<0.0001$) comparing baseline with lockdown period.
Specific evidence of impact on children (e.g. 30% increase of weight during two weeks of implementation of stay at	N/A

home policy; or: 'calculated 365,000 extra malaria deaths calculated as a consequence of disruption of bed nets distribution)	
Specific evidence of impact on women (e.g. 20% reduction of female employment in the fishing sector, as a result of decreased demand for fish during the epidemic)	Maternal mental health deteriorated during the lockdown. Symptoms of depression increased among women during lockdown (10, 3–17; 6-point increase, in the IQR 0–11 scale); p<0.0001. Emotional violence increased with respect to baseline, including insults (initially reported by 19.9%) 68.4% reported an increase, humiliation [66.0%] of 191 reported an increase), and intimidation (reported by 13.5% [68.7%] of 291 reported an increase). Physical violence (eg, being slapped or having something thrown at them) was initially reported by 6.5%, [56%] of 135 reported an increase). Sexual violence was less common (3.0%, 2.3–3.8), but of those affected, 33 (50.8%) of 65 reported it had increased since the lockdown.
Relevance and importance of the paper for our own systematic review	☐ Key paper for the review
Other comments or issues to be noted about this paper	Very strong paper, particularly for the domestic violence domain. But also presenting data to be used for poverty and livelyhoods.

Table 6: Data extraction form for SLR on the health impact of lockdown measures for women and children in LMICs

Title of the paper:	Potential impact of the COVID-19 pandemic on HIV, tuberculosis, and malaria in low-income and middle-income countries: a modelling study
Author(s) and date:	Hogan et al (2020)
Quality appraisal according to the relevant Johanna Briggs Institute tool (please cross out or delete accordingly)	☐ Include
Epidemic of reference:	COVID-19
Research methods employed (e.g. qualitative interviews; modelling; surveys; systematic review)	Modelling
Specific lockdown measure investigated in the study (e.g. generic quarantine or unspecified lockdown measure; curfew; stay at home policy; travel restrictions; school closures; market and trade shutdowns; social distancing)	Interruption of services, namely: interruption of ART, disruption of TB early TB diagnosis; interruption of planned campaigns for malaria nets distribution

Broad area of impact explored in the study (please cross out or delete as appropriate)	44. Disruption of routine services for mother and child services;<br>45. Other (deaths and increased incidence of Aids, TB and malaria).
Evidence of impact to the general population from lockdown measures (e.g. 10% decrease of disposable income before and after the peak of the epidemic; 30% reduction of hospital visits for maternal services)	Under different scenarios of COVID-19 mitigation and suppression, it was calculated there would be up to 596 extra deaths per million people due to HIV-Aids, 987 pere million people due to TB, and up to 1,018 per million people due to malaria.
Specific evidence of impact on children (e.g. 30% increase of weight during two weeks of implementation of stay at home policy; or: 'calculated 365,000 extra malaria deaths calculated as a consequence of disruption of bed nets distribution)	
Specific evidence of impact on women (e.g. 20% reduction of female employment in the fishing sector, as a result of decreased demand for fish during the epidemic)	
Relevance and importance of the paper for our own systematic review	☐ Paper of some importance for some sections of the review
Other comments or issues to be noted about this paper	It would be cumbersome to make a link to the specific modelled extra deaths for children and women, although a substantial proportion of malaria and aids deaths can be attributed to such vunerable popoulations in LICs settings.

Table 7: Data extraction form for SLR on the health impact of lockdown measures for women and children in LMICs

Title of the paper:	Potential effects of disruption to HIV programmes in sub-Saharan Africa caused by COVID-19: results from multiple mathematical models
Author(s) and date:	Jewell et al (2020)
Quality appraisal according to the relevant Johanna Briggs Institute tool (please cross out or delete accordingly)	☐ Include
Epidemic of reference:	COVID-19
Research methods employed (e.g. qualitative interviews; modelling; surveys; systematic review)	Quantitative modelling to estimate the impact of COVID-19 on the AIDS prevalence and deaths in Africa

Specific lockdown measure investigated in the study (e.g. generic quarantine or unspecified lockdown measure; curfew; stay at home policy; travel restrictions; school closures; market and trade shutdowns; social distancing)	• Suspension of HIv testing;<br>• Interruption of ART drugs and services<br>• Interruption of mother-to-child transmission services;<br>• Interruption of condom supplies.
Broad area of impact explored in the study (please cross out or delete as appropriate)	46. Disruption of routine services for mother and child services;<br>47. Increases in social vulnerability, poverty and impact on human development;
Evidence of impact to the general population from lockdown measures (e.g. 10% decrease of disposable income before and after the peak of the epidemic; 30% reduction of hospital visits for maternal services)	According to different scenarios of disruption of services (from 20% to 100% disruption), the models predict between 92,000 and 956,000 excess deaths in SS Africa in one year.
Specific evidence of impact on children (e.g. 30% increase of weight during two weeks of implementation of stay at home policy; or: 'calculated 365,000 extra malaria deaths calculated as a consequence of disruption of bed nets distribution)	Interruption of ART would increase mother-to-child transmission of HIV by approximately 1·6 times, with a similar increase of mortality for newborns.
Specific evidence of impact on women (e.g. 20% reduction of female employment in the fishing sector, as a result of decreased demand for fish during the epidemic)	N/A
Relevance and importance of the paper for our own systematic review	☐ Key paper for the review
Other comments or issues to be noted about this paper	Difficult to extract specific mortality predictions for children.

Table 8: Data extraction form for SLR on the health impact of lockdown measures for women and children in LMICs

Title of the paper:	Implications of COVID-19-induced nationwide lockdown on children's behaviour in Punjab, India
Author(s) and date:	Kaur Sama et al (2020)
Quality appraisal according to the relevant Johanna Briggs	☐ Include

Institute tool (please cross out or delete accordingly)	
Epidemic of reference:	COVID-19
Research methods employed (e.g. qualitative interviews; modelling; surveys; systematic review)	Telephone questionnaire and interviews
Specific lockdown measure investigated in the study (e.g. generic quarantine or unspecified lockdown measure; curfew; stay at home policy; travel restrictions; school closures; market and trade shutdowns; social distancing)	Stay at home measures; closure of schools.
Broad area of impact explored in the study (please cross out or delete as appropriate)	48. Mental Health;<br>49. Dietary patterns and nutrition;
Evidence of impact to the general population from lockdown measures (e.g. 10% decrease of disposable income before and after the peak of the epidemic; 30% reduction of hospital visits for maternal services)	N/A
Specific evidence of impact on children (e.g. 30% increase of weight during two weeks of implementation of stay at home policy; or: 'calculated 365,000 extra malaria deaths calculated as a consequence of disruption of bed nets distribution)	73.15% and 51.25% of the children were having signs of increased irritation and anger, respectively; 18.7% and 17.6% of the parents also mentioned the symptoms of depression and anxiety, respectively, among their children, which were also augmented by the changes in their diet, sleep, weight and more usage of the electronic equipment.
Specific evidence of impact on women (e.g. 20% reduction of female employment in the fishing sector, as a result of decreased demand for fish during the epidemic)	N/A
Relevance and importance of the paper for our own systematic review	☐ Secondary paper
Other comments or issues to be noted about this paper	

Table S1. Data extraction form for SLR on the health impact of lockdown measures for women and children in LMICs

Title of the paper:	Consensus building around nutrition lessons from the 2014–16 Ebola virus disease outbreak in Guinea and Sierra Leone
Author(s) and date:	Stephen R Kodish , Aline Simen-Kapeu, Jean-Max Beauliere, Ismael Ngnie-Teta, Mohammed B Jalloh, Solade Pyne-Bailey, Helen Schwartz and James PWirth
Quality appraisal according to the relevant Johanna Briggs Institute tool (please cross out or delete accordingly)	☐ Include<br>☐ Exclude<br>☐ Seek further information
Epidemic of reference:	Ebola
Research methods employed (e.g. qualitative interviews; modelling; surveys; systematic review)	Qualitative – workshops with 17 and 19 stakeholders
Specific lockdown measure investigated in the study (e.g. generic quarantine or unspecified lockdown measure; curfew; stay at home policy; travel restrictions; school closures; market and trade shutdowns; social distancing)	Not really a focus on the impact of lockdown or control measures, though this is touched upon a little
Broad area of impact explored in the study (please cross out or delete as appropriate)	50. Dietary patterns and nutrition;<br>51. Demand for healthcare / Healthcare seeking behaviour;<br>52.
Evidence of impact to the general population from lockdown measures (e.g. 10% decrease of disposable income before and after the peak of the epidemic; 30% reduction of hospital visits for maternal services)	
Specific evidence of impact on children (e.g. 30% increase of weight during two weeks of implementation of stay at home policy; or: 'calculated 365,000 extra malaria deaths calculated as a consequence of disruption of bed nets distribution)	Stakeholders mentioned that infant and young child nutrition was adversely impacted by<br><br>Poor access to the health system<br>Household food insecurity<br>Changing breastfeeding practices.
Specific evidence of impact on women (e.g. 20% reduction of female employment in the fishing sector, as a result of	

decreased demand for fish during the epidemic)	
Relevance and importance of the paper for our own systematic review	☐ Secondary paper
Other comments or issues to be noted about this paper	Potentially could also exclude this paper, not sure it really addresses lockdowns specifically

Table S1. Data extraction form for SLR on the health impact of lockdown measures for women and children in LMICs

Title of the paper:	A qualitative study to understand how Ebola Virus Disease affected nutrition in Sierra Leone—A food value-chain framework for improving future response strategies
Author(s) and date:	Stephen R. Kodish, Frank Bio, Rachel Oemcke, James Conteh, Jean Max Beauliere, Solade Pyne-Bailey, Fabian Rohner, Ismael Ngnie-Teta, Mohammad B. Jalloh, James P. Wirth, 2020
Quality appraisal according to the relevant Johanna Briggs Institute tool (please cross out or delete accordingly)	☐ Include
Epidemic of reference:	Ebola Virus Disease 2014-2016 outbreak
Research methods employed (e.g. qualitative interviews; modelling; surveys; systematic review)	Qualitative methods comprised of 42 in-depth interviews (21 Key informants and 21 community informants)
Specific lockdown measure investigated in the study (e.g. generic quarantine or unspecified lockdown measure; curfew; stay at home policy; travel restrictions; school closures; market and trade shutdowns; social distancing)	No specific measure as such. The study includes looking at the impact of control measures on nutrition, but this is not the only focus.<br><br>"To reduce the spread of EVD in Sierra Leone, the government restricted people's movements by blocking roads and imposing household and community quarantines."
Broad area of impact explored in the study (please cross out or delete as appropriate)	53. Dietary patterns and nutrition
Evidence of impact to the general population from lockdown measures (e.g. 10% decrease of disposable income before and after the peak of the epidemic; 30% reduction of hospital visits for maternal services)	Negative impact on food security and nutrition due to reduction in production (people could not go to their farms or the market)<br>Movement was restricted, therefore food was scarce.<br>Shops were closed<br>Most mothers and family heads were not able to work<br><br>Overall conceptual framework:<br>• Reduction in food production<br>○ Reduced planting<br>○ Reduced agricultural capacity<br>• Reduced food processing<br>• Challenges related to bringing food to market (i.e no household surplus)

	• Prices high<br>• Limited markets open, some entirely closed<br><br>Resulting in<br>Reduced food availability and access<br>Altered infants and young children feeding practices
Specific evidence of impact on children (e.g. 30% increase of weight during two weeks of implementation of stay at home policy; or: 'calculated 365,000 extra malaria deaths calculated as a consequence of disruption of bed nets distribution)	For those that could afford it, they were unable to buy baby food at the shops<br><br>Altered infants and young children feeding practices<br><br>Reduced screening for malnutrition cases<br>• Travel restrictions limiting people's movement<br>• Reduced health-seeking behaviors from less movement and distrust<br>• Early denial of outbreak and distrust among community
Specific evidence of impact on women (e.g. 20% reduction of female employment in the fishing sector, as a result of decreased demand for fish during the epidemic)	No specific issues reported
Relevance and importance of the paper for our own systematic review	☐ Key paper for the review
Other comments or issues to be noted about this paper	

Table 9: Data extraction form for SLR on the health impact of lockdown measures for women and children in LMICs

Title of the paper:	The Ebola-effect in Guinea 2014-15: Tangled trends of malaria care in children under-five
Author(s) and date:	Kolie et al (2018)
Quality appraisal according to the relevant Johanna Briggs Institute tool (please cross out or delete accordingly)	☐ Include
Epidemic of reference:	Ebola
Research methods employed (e.g. qualitative interviews; modelling; surveys; systematic review)	Cross sectional analysis of routine malaria surveillance data
Specific lockdown measure investigated in the study (e.g. generic quarantine or unspecified lockdown	Stay at home; curfews and suspension of services

measure; curfew; stay at home policy; travel restrictions; school closures; market and trade shutdowns; social distancing)	
Broad area of impact explored in the study (please cross out or delete as appropriate)	54. Disruption of routine services for mother and child services;<br>55. Demand for healthcare / Healthcare seeking behaviour;
Evidence of impact to the general population from lockdown measures (e.g. 10% decrease of disposable income before and after the peak of the epidemic; 30% reduction of hospital visits for maternal services)	During the peak of the Ebola outbreak, there was a significant decrease in oral antimalarial drug administration, which corresponded to an increase in injectable antimalarial treatments. Stock-outs in rapid diagnostic tests were evident and prolonged in GueÂckeÂdou during the outbreak, while more limited in Koubia.
Specific evidence of impact on children (e.g. 30% increase of weight during two weeks of implementation of stay at home policy; or: 'calculated 365,000 extra malaria deaths calculated as a consequence of disruption of bed nets distribution)	In the Ebola-affected district of GueÂckeÂdou, there was a 30% decrease in total clinical visits, malaria testing for >5 children.
Specific evidence of impact on women (e.g. 20% reduction of female employment in the fishing sector, as a result of decreased demand for fish during the epidemic)	N/A
Relevance and importance of the paper for our own systematic review	☐ Secondary paper
Other comments or issues to be noted about this paper	

Table 10: Data extraction form for SLR on the health impact of lockdown measures for women and children in LMICs

Title of the paper:	The impact of a prolonged ebola outbreak on measles elimination activities in Guinea, Liberia and Sierra Leone, 2014-2015
Author(s) and date:	Maresha et al (2020)
Quality appraisal according to the relevant Johanna Briggs Institute tool (please cross out or delete accordingly)	☐ Include
Epidemic of reference:	Ebola
Research methods employed (e.g. qualitative interviews;	Review of routine surveillance and service delivery data

modelling; surveys; systematic review)	
Specific lockdown measure investigated in the study (e.g. generic quarantine or unspecified lockdown measure; curfew; stay at home policy; travel restrictions; school closures; market and trade shutdowns; social distancing)	Interruption of services and closure of facilities.
Broad area of impact explored in the study (please cross out or delete as appropriate)	56. Disruption of routine services for mother and child services;<br>57. Demand for healthcare / Healthcare seeking behaviour;
Evidence of impact to the general population from lockdown measures (e.g. 10% decrease of disposable income before and after the peak of the epidemic; 30% reduction of hospital visits for maternal services)	Liberia and Guinea experienced a sharp decline of more than 25% in the monthly number of children vaccinated against measles in 2014 and 2015 as compared to the previous years, while there was no reported decline in Sierra Leone.
Specific evidence of impact on children (e.g. 30% increase of weight during two weeks of implementation of stay at home policy; or: 'calculated 365,000 extra malaria deaths calculated as a consequence of disruption of bed nets distribution)	In Liberia, the mean monthly number of children vaccinated for measles declined by 30% in 2014 and by a further 25% during 2015;<br>in Guinea the monthly average declined by 33% in 2014 and by 26% in 2015.<br>In Sierra Leone the decline was much smaller, as the MCV1 coverage showed a 3% decline from an average of 86% for 2012 – 2013 to 83% in 2016 – 2017.
Specific evidence of impact on women (e.g. 20% reduction of female employment in the fishing sector, as a result of decreased demand for fish during the epidemic)	N/A
Relevance and importance of the paper for our own systematic review	☐ Secondary paper
Other comments or issues to be noted about this paper	There are some issues with the statistical methods used in the study.

Table S1. Data extraction form for SLR on the health impact of lockdown measures for women and children in LMICs

Title of the paper:	Awareness and concerns about novel coronavirus disease 2019 (COVID-19) among parents of pediatric liver transplant recipients

Author(s) and date:	Jagadeesh Menon, Naresh Shanmugam, Kinisha Patel, Abdul Hakeem, Mettu Srinivas Reddy, Mohamed Rela
Quality appraisal according to the relevant Johanna Briggs Institute tool (please cross out or delete accordingly)	☐ Include<br>☐ Exclude<br>☐ Seek further information
Epidemic of reference:	Covid-19
Research methods employed (e.g. qualitative interviews; modelling; surveys; systematic review)	Web-based survey conducted with 106 parents of post-liver transplant children
Specific lockdown measure investigated in the study (e.g. generic quarantine or unspecified lockdown measure; curfew; stay at home policy; travel restrictions; school closures; market and trade shutdowns; social distancing)	Not focused on the impact of lockdown
Broad area of impact explored in the study (please cross out or delete as appropriate)	Awareness and sources of information
Evidence of impact to the general population from lockdown measures (e.g. 10% decrease of disposable income before and after the peak of the epidemic; 30% reduction of hospital visits for maternal services)	
Specific evidence of impact on children (e.g. 30% increase of weight during two weeks of implementation of stay at home policy; or: 'calculated 365,000 extra malaria deaths calculated as a consequence of disruption of bed nets distribution)	
Specific evidence of impact on women (e.g. 20% reduction of female employment in the fishing sector, as a result of decreased demand for fish during the epidemic)	
Relevance and importance of the paper for our own systematic review	Not relevant
Other comments or issues to be noted about this paper	Exclude this paper, it doesn't address the impact of lockdown

Table S1. Data extraction form for SLR on the health impact of lockdown measures for women and children in LMICs

Title of the paper:	COVID-19 induced economic loss and ensuring food security for vulnerable groups: Policy implications from Bangladesh
Author(s) and date:	Khondoker Abdul Mottaleb, Mohammed Mainuddin, Tetsushi Sonobe
Quality appraisal according to the relevant Johanna Briggs Institute tool (please cross out or delete accordingly)	☐ Include<br><br>No 'modelling' appraisal tool
Epidemic of reference:	Covid-19
Research methods employed (e.g. qualitative interviews; modelling; surveys; systematic review)	Modelling<br><br>"The present study uses information from 50,000 economically active workers in Bangladesh,<br>collected by the Bangladesh Bureau of Statistics (BBS), to quantify the economic loss due<br>to the COVID-19 lockdown based on the lost wage earnings of the daily wage workers in the<br>farm and nonfarm sectors of Bangladesh."<br><br>"this study quantifies the daily economic loss due to wage earning forgone of daily wage based workers both in farm and nonfarm sectors."<br><br>Econometric analysis<br>Estimates daily wages of farm and non-farm workers<br>Multiplies these wages by the number of workers
Specific lockdown measure investigated in the study (e.g. generic quarantine or unspecified lockdown measure; curfew; stay at home policy; travel restrictions; school closures; market and trade shutdowns; social distancing)	Covers lockdown in general
Broad area of impact explored in the study (please cross out or delete as appropriate)	58. Increases in social vulnerability, poverty and impact on human development;
Evidence of impact to the general population from lockdown measures (e.g. 10% decrease of disposable income before and after the peak of the epidemic; 30% reduction of hospital visits for maternal services)	The estimated daily wage earnings for farm workers is BDT 272.2, and BDT 361.5<br>for nonfarm workers<br>In 2016–17, there were 24.7 million workers in the farm sector and 36.1 million in the nonfarm<br>sector

	Under the assumption of a complete lockdown with no-one allowed to work, the economic loss in one day is estimated at BDT 5389.03 million or approximately US$ 64.2 million. Assuming 50% of the daily wage workers are not allowed to work and the rest are, the economic loss/day will be BDT 2694.5 million or US$ 32.1 million.<br><br>we have estimated that on average it is necessary to provide daily BDT 51–104 or around US $ 1 per daily wage-based households during the COVID-19 induced lockdown time. It is important to mention here is that, the suggested minimum support US$ 1/day/household is calculated based on considering only food expenditure.
Specific evidence of impact on children (e.g. 30% increase of weight during two weeks of implementation of stay at home policy; or: 'calculated 365,000 extra malaria deaths calculated as a consequence of disruption of bed nets distribution)	
Specific evidence of impact on women (e.g. 20% reduction of female employment in the fishing sector, as a result of decreased demand for fish during the epidemic)	
Relevance and importance of the paper for our own systematic review	☐ Secondary paper
Other comments or issues to be noted about this paper	Shows potential economic impacts of control measures

Table 11: Data extraction form for SLR on the health impact of lockdown measures for women and children in LMICs

Title of the paper:	The impact of COVID-19 lockdown in a developing country: narratives of self-employed women in Ndola, Zambia
Author(s) and date:	Nyashanu et al (2020)
Quality appraisal according to the relevant Johanna Briggs Institute tool (please cross out or delete accordingly)	☐ Include<br>☐ Exclude<br>☐ Seek further information
Epidemic of reference:	COVID-19 in Zambia
Research methods employed (e.g. qualitative interviews; modelling; surveys; systematic review)	In-depth qualitative interviews (40 women)
Specific lockdown measure investigated in the study (e.g.	Stay at home, closure of markets and restrictions on comerce

generic quarantine or unspecified lockdown measure; curfew; stay at home policy; travel restrictions; school closures; market and trade shutdowns; social distancing)	
Broad area of impact explored in the study (please cross out or delete as appropriate)	59. Demand for healthcare / Healthcare seeking behaviour;<br>60. Increases in social vulnerability, poverty and impact on human development;
Evidence of impact to the general population from lockdown measures (e.g. 10% decrease of disposable income before and after the peak of the epidemic; 30% reduction of hospital visits for maternal services)	
Specific evidence of impact on children (e.g. 30% increase of weight during two weeks of implementation of stay at home policy; or: 'calculated 365,000 extra malaria deaths calculated as a consequence of disruption of bed nets distribution)	100% of the research participants reported challenges of keeping young people indoors due to boredom and short concentration span. They also reported difficulties in exercising social distancing while in the house due to the size of the room.
Specific evidence of impact on women (e.g. 20% reduction of female employment in the fishing sector, as a result of decreased demand for fish during the epidemic)	100% of the research participants reported inadequate food supplies as a result of closing down of their self-run businesses. 100% of the research participants reported hopelessness to revive their business due to spending up of their savings during COVID-19 Lockdown. They also reported a loss of all their perishable goods at the start of the lockdown. 100% of the research participants reported poor access to reproductive health services due to poor transport network during lockdown. They also reported poor access to maternal health services.
Relevance and importance of the paper for our own systematic review	▣ Paper of some importance for some sections of the review
Other comments or issues to be noted about this paper	Interesting qualitative paper on livelyhoods of women, but the reported evidence from the intereviews is rather weak

Table 12: Data extraction form for SLR on the health impact of lockdown measures for women and children in LMICs

Title of the paper:	Psychological impact of mass quarantine on population during pandemics-The COVID-19 Lock-Down (COLD) study
Author(s) and date:	Pandey et al (2020)

Quality appraisal according to the relevant Johanna Briggs Institute tool (please cross out or delete accordingly)	☐ Include
Epidemic of reference:	COVID-19
Research methods employed (e.g. qualitative interviews; modelling; surveys; systematic review)	Cross sectional survey to quantify prevalence of depressiona and anxiety in India
Specific lockdown measure investigated in the study (e.g. generic quarantine or unspecified lockdown measure; curfew; stay at home policy; travel restrictions; school closures; market and trade shutdowns; social distancing)	Quarantine
Broad area of impact explored in the study (please cross out or delete as appropriate)	61. Mental Health;
Evidence of impact to the general population from lockdown measures (e.g. 10% decrease of disposable income before and after the peak of the epidemic; 30% reduction of hospital visits for maternal services)	The reported prevalence of depression was 30.5%. Anxiety was reported by 22.4%, followed by stress which was seen in 10.8% of respondents. In the third week the incidence of depression (37.8% versus 23.4%; p<0.001), anxiety (26.6% versus 18.2%; p<0.001) and stress (12.2% versus 9.3%; p<0.045) was reported to be significantly higher as compared to second week.
Specific evidence of impact on children (e.g. 30% increase of weight during two weeks of implementation of stay at home policy; or: 'calculated 365,000 extra malaria deaths calculated as a consequence of disruption of bed nets distribution)	N/A
Specific evidence of impact on women (e.g. 20% reduction of female employment in the fishing sector, as a result of decreased demand for fish during the epidemic)	Women were more susceptible to suffer from all forms of psychological symptoms (depression, anxiety and stress) as compared to men;Significantly higher proportion of women had mild to severe level of depression (P<0.002), anxiety (P<0.002) and stress (P<0.001) as compared to the men.
Relevance and importance of the paper for our own systematic review	☐ Key paper for the review
Other comments or issues to be noted about this paper	Results are not generalisable to the whole population, as they are from a smal sample of 1,395 people.

Table S1. Data extraction form for SLR on the health impact of lockdown measures for women and children in LMICs

Title of the paper:	Attitudes and collateral psychological effects of COVID‐19 in pregnant women in Colombia
Author(s) and date:	Miguel Parra-Saavedra et al
Quality appraisal according to the relevant Johanna Briggs Institute tool (please cross out or delete accordingly)	☐ Include
Epidemic of reference:	Covid-19
Research methods employed (e.g. qualitative interviews; modelling; surveys; systematic review)	Cross sectional online survey of pregnant women on 7 cities<br>N=946 valid surveys
Specific lockdown measure investigated in the study (e.g. generic quarantine or unspecified lockdown measure; curfew; stay at home policy; travel restrictions; school closures; market and trade shutdowns; social distancing)	No specific measure<br><br>"Women were evaluated during the mitigation phase of the SARS-Co-2 pandemic between April 13 and May 18, 2020"
Broad area of impact explored in the study (please cross out or delete as appropriate)	62. Mental Health;<br>63.
Evidence of impact to the general population from lockdown measures (e.g. 10% decrease of disposable income before and after the peak of the epidemic; 30% reduction of hospital visits for maternal services)	
Specific evidence of impact on children (e.g. 30% increase of weight during two weeks of implementation of stay at home policy; or: 'calculated 365,000 extra malaria deaths calculated as a consequence of disruption of bed nets distribution)	
Specific evidence of impact on women (e.g. 20% reduction of female employment in the fishing sector, as a result of decreased demand for fish during the epidemic)	The presence of symptoms associated with anxiety was reported by 50.1% of the women. Similarly, 49% of the participants reported insomnia and 25.4% reported symptoms of depression. The authors note this is high but there is no 'pre-epidemic' comparison

Relevance and importance of the paper for our own systematic review	☐ Paper of some importance for some sections of the review<br>☐
Other comments or issues to be noted about this paper	Describes levels of psychological impacts during mitigation measures but does not offer much comparison with pre-covid times

Table S1. Data extraction form for SLR on the health impact of lockdown measures for women and children in LMICs

Title of the paper:	Early estimates of the indirect effects of the COVID-19 pandemic on maternal and child mortality in low-income and middle-income countries: a modelling study
Author(s) and date:	Timothy Roberton, Emily D Carter, Victoria B Chou, Angela R Stegmuller, Bianca D Jackson, Yvonne Tam, Talata Sawadogo-Lewis, Neff Walker
Quality appraisal according to the relevant Johanna Briggs Institute tool (please cross out or delete accordingly)	☐ Include<br>☐ Exclude<br>☐ Seek further information
Epidemic of reference:	Covid
Research methods employed (e.g. qualitative interviews; modelling; surveys; systematic review)	Modelling<br><br>"We modelled three scenarios in which the coverage of essential maternal and child health interventions is reduced by 9·8–51·9% and the prevalence of wasting is increased by 10–50%."<br><br>Data covers 118 LMICs
Specific lockdown measure investigated in the study (e.g. generic quarantine or unspecified lockdown measure; curfew; stay at home policy; travel restrictions; school closures; market and trade shutdowns; social distancing)	No specific measure<br><br>"Governments are restricting population movement by closing borders, reducing public transport, halting nonessential activities, and issuing shelter-in-place orders"<br><br>But other mechanisms are also identified, so the findings cannot be solely attributed to lockdowns
Broad area of impact explored in the study (please cross out or delete as appropriate)	64. Disruption of routine services for mother and child services;<br>65. Demand for healthcare / Healthcare seeking behaviour;
Evidence of impact to the general population from lockdown measures (e.g. 10% decrease of disposable income before and after the peak of the epidemic; 30% reduction of hospital visits for maternal services)	
Specific evidence of impact on children (e.g. 30% increase of weight during two weeks of	"scenario 1 (smallest reductions) resulting in an additional 42 240 child deaths per month, and scenario 3 (greatest reductions)

implementation of stay at home policy; or: 'calculated 365,000 extra malaria deaths calculated as a consequence of disruption of bed nets distribution)	resulting in an additional and 192 830 child deaths per month"<br><br>"The additional child deaths would represent relative increases of 9·8% (scenario 1), 17·3% (scenario 2), and 44·7% (scenario 3) in child deaths per month."<br><br>"In children, an increase in wasting prevalence would account for 18–23% of additional deaths, depending on the scenario, while reduced coverage of antibiotics for pneumonia and neonatal sepsis and of oral rehydration solution for diarrhoea would together account for around 41% of additional child deaths"
Specific evidence of impact on women (e.g. 20% reduction of female employment in the fishing sector, as a result of decreased demand for fish during the epidemic)	"with scenario 1 (smallest reductions) resulting in an additional 2030 maternal deaths , and scenario 3 (greatest reductions) resulting in an additional 9450 maternal deaths"<br><br>"The additional maternal deaths would represent relative increases of 8·3% (scenario 1), 14·7% (scenario 2), and 38·6% (scenario 3) in maternal deaths per month"<br><br>"The reduced coverage of four childbirth interventions (namely, parenteral administration of uterotonics, antibiotics, and anticonvulsants, and clean birth environments, which reduce mortality due to postpartum haemorrhage, maternal sepsis, and eclampsia) would account for approximately 60% of additional maternal deaths"
Relevance and importance of the paper for our own systematic review	☐ Paper of some importance for some sections of the review<br>☐
Other comments or issues to be noted about this paper	Modelling paper (good as it is)

Table 13: Data extraction form for SLR on the health impact of lockdown measures for women and children in LMICs

Title of the paper:	Perceived stress, parental stress, and parenting during covid-19 lockdown: A preliminary study
Author(s) and date:	Sahithya et al (2020)
Quality appraisal according to the relevant Johanna Briggs Institute tool (please cross out or delete accordingly)	☐ Seek further clarification (sample not representative)
Epidemic of reference:	COVID-19
Research methods employed (e.g. qualitative interviews; modelling; surveys; systematic review)	Online cross sectional survey
Specific lockdown measure investigated in the study (e.g.	School closures

generic quarantine or unspecified lockdown measure; curfew; stay at home policy; travel restrictions; school closures; market and trade shutdowns; social distancing)	
Broad area of impact explored in the study (please cross out or delete as appropriate)	66. Mental Health;
Evidence of impact to the general population from lockdown measures (e.g. 10% decrease of disposable income before and after the peak of the epidemic; 30% reduction of hospital visits for maternal services)	Majority of the parents reported moderate (63%) to high (4%) perceived stress since the COVID-19 lockdown, 37% reported feeling more stressed as a parent after the lockdown, and 8% of the parents said they needed to talk to a counsellor/psychologist
Specific evidence of impact on children (e.g. 30% increase of weight during two weeks of implementation of stay at home policy; or: 'calculated 365,000 extra malaria deaths calculated as a consequence of disruption of bed nets distribution)	It was found that parents who reported increased shouting, yelling and screaming ($t=5.69$, $p<.001$, $g=1.06$), spanking or slapping their child($t=2.96$, $p=.004$, $g=0.43$), or those who reported being preoccupied with worries and unable to focus on parenting ($t=4.11$, $p<.001$, $g=0.78$), or had difficulties in disciplining the child ($t=5.56$, $p<0.001$, $g=1.00$) had higher parental stress compared to parents who did not experience these difficulties.
Specific evidence of impact on women (e.g. 20% reduction of female employment in the fishing sector, as a result of decreased demand for fish during the epidemic)	It was found that mothers ($t=2.06$, $p=.041$) had significantly higher parental stress when compared to fathers.
Relevance and importance of the paper for our own systematic review	☐ Secondary paper
Other comments or issues to be noted about this paper	The sampling is not representative, and I am not sure we should include these results…

Table 14: Data extraction form for SLR on the health impact of lockdown measures for women and children in LMICs

Title of the paper:	Installing oncofertility programs for common cancers in limited resource settings (Repro-Can-OPEN Study): An extrapolation during the global crisis of Coronavirus (COVID-19) pandemic
Author(s) and date:	Salama et al (2020)
Quality appraisal according to the relevant Johanna Briggs Institute tool (please cross out or delete accordingly)	☐ Include
Epidemic of reference:	COVID-19

Research methods employed (e.g. qualitative interviews; modelling; surveys; systematic review)	Multicountry survey questionnaire included questions on the availability and degree of utilization of fertility preservation options in case of childhood cancer, breast cancer, and blood cancer.
Specific lockdown measure investigated in the study (e.g. generic quarantine or unspecified lockdown measure; curfew; stay at home policy; travel restrictions; school closures; market and trade shutdowns; social distancing)	Cancelation or postponement of oncological medical treatments.
Broad area of impact explored in the study (please cross out or delete as appropriate)	67. Disruption of routine services for mother and child services;<br>68. Demand for healthcare / Healthcare seeking behaviour;
Evidence of impact to the general population from lockdown measures (e.g. 10% decrease of disposable income before and after the peak of the epidemic; 30% reduction of hospital visits for maternal services)	There was a substantial reduction of the availability score for Available fertility preservation options for girls with cancer in India (Yes ++)
Specific evidence of impact on children (e.g. 30% increase of weight during two weeks of implementation of stay at home policy; or: 'calculated 365,000 extra malaria deaths calculated as a consequence of disruption of bed nets distribution)	Significant reduction of availability of cancer treatment for boys in India (Yes ++) for testicular cancer treatment, as well as availability of chemio and radio therapy
Specific evidence of impact on women (e.g. 20% reduction of female employment in the fishing sector, as a result of decreased demand for fish during the epidemic)	There was a substantial reduction of the availability score for Available fertility preservation options for girls with ovarian and breast cancer cancer in India and Nigeria (Yes ++)
Relevance and importance of the paper for our own systematic review	☐ Paper of some importance for some sections of the review
Other comments or issues to be noted about this paper	

Table S1. Data extraction form for SLR on the health impact of lockdown measures for women and children in LMICs

Title of the paper:	COVID-19 containment and its unrestrained impact on epilepsy management in resource-limited areas of Pakistan

Author(s) and date:	Tayyaba Saleem, Nadeem Sheikh, Muddasir Hassan Abbasi , Iram Javed , Muhammad Babar khawar
Quality appraisal according to the relevant Johanna Briggs Institute tool (please cross out or delete accordingly)	☐ Include
Epidemic of reference:	Covid-19
Research methods employed (e.g. qualitative interviews; modelling; surveys; systematic review)	Cross sectional survey of caregivers of pediatric patients with active epilepsy<br>n=213<br><br>Participants had already been recruited for a different epilepsy study
Specific lockdown measure investigated in the study (e.g. generic quarantine or unspecified lockdown measure; curfew; stay at home policy; travel restrictions; school closures; market and trade shutdowns; social distancing)	No specific measure, but references 'containment measures'
Broad area of impact explored in the study (please cross out or delete as appropriate)	69. Disruption of routine services for mother and child services;
Evidence of impact to the general population from lockdown measures (e.g. 10% decrease of disposable income before and after the peak of the epidemic; 30% reduction of hospital visits for maternal services)	38% lost their job during the crisis
Specific evidence of impact on children (e.g. 30% increase of weight during two weeks of implementation of stay at home policy; or: 'calculated 365,000 extra malaria deaths calculated as a consequence of disruption of bed nets distribution)	64% had an appointment cancelled due to COVID-19 pandemic<br>17.4% had medication discontinued due to disruption<br>26.8% reported worsening of seizures<br><br>Reliance on free AED supply from hospital:<br>Totally 65 (30.5)<br>Partially 122 (57.3)
Specific evidence of impact on women (e.g. 20% reduction of female employment in the fishing sector, as a result of decreased demand for fish during the epidemic)	
Relevance and importance of the paper for our own systematic review	☐ Paper of some importance for some sections of the review

Other comments or issues to be noted about this paper	

Table S1. Data extraction form for SLR on the health impact of lockdown measures for women and children in LMICs

Title of the paper:	Challenges to Ebola preparedness during an ongoing outbreak: An analysis of borderland livelihoods and trust in Uganda
Author(s) and date:	Megan M. Schmidt-Sane, Jannie O. Nielsen2, Mandi Chikombero,<br>Douglas Lubowa, Miriam Lwanga, Jonathan Gamusi, Richard Kabanda, David Kaawa-Mafigiri
Quality appraisal according to the relevant Johanna Briggs Institute tool (please cross out or delete accordingly)	Exclude but not on JBI criteria
Epidemic of reference:	Ebola
Research methods employed (e.g. qualitative interviews; modelling; surveys; systematic review)	Qualitative study with 287 participants
Specific lockdown measure investigated in the study (e.g. generic quarantine or unspecified lockdown measure; curfew; stay at home policy; travel restrictions; school closures; market and trade shutdowns; social distancing)	No measure. Paper does not focus on the impact of lockdown or control measures
Broad area of impact explored in the study (please cross out or delete as appropriate)	70. Increases in social vulnerability, poverty and impact on human development;<br><br>If any
Evidence of impact to the general population from lockdown measures (e.g. 10% decrease of disposable income before and after the peak of the epidemic; 30% reduction of hospital visits for maternal services)	Ebola control measures hinder or restrict movement<br>across borders and has infringed on individual decisions when to travel. Some may decide to<br>proceed through informal POE to avoid Ebola surveillance activities at the formal POE. Therefore,<br>livelihood strategies impact multiple aspects of Ebola prevention–from driving continued<br>cross-border movement, to limiting cross-border movement (particularly through official<br>POEs), to shifting decisions on how and when to cross the border
Specific evidence of impact on children (e.g. 30% increase of weight during two weeks of implementation of stay at home policy; or: 'calculated 365,000 extra malaria deaths	None mentioned

calculated as a consequence of disruption of bed nets distribution)	
Specific evidence of impact on women (e.g. 20% reduction of female employment in the fishing sector, as a result of decreased demand for fish during the epidemic)	Non mentioned
Relevance and importance of the paper for our own systematic review	Exclude
Other comments or issues to be noted about this paper	I would probably exclude this paper – it touches upon the impact of movement restrictions, but these are not central to the paper

Table 15: Data extraction form for SLR on the health impact of lockdown measures for women and children in LMICs

Title of the paper:	Women's mental health: acute impact of COVID-19 pandemic on domestic violence
Author(s) and date:	Sediri et al (2020)
Quality appraisal according to the relevant Johanna Briggs Institute tool (please cross out or delete accordingly)	☐ Include
Epidemic of reference:	COVID-19
Research methods employed (e.g. qualitative interviews; modelling; surveys; systematic review)	Online survey (sample of 751)
Specific lockdown measure investigated in the study (e.g. generic quarantine or unspecified lockdown measure; curfew; stay at home policy; travel restrictions; school closures; market and trade shutdowns; social distancing)	• Generic lockdown measures by the Tunisian govt;<br>• Travel or movement restriction, suspension of work and studies
Broad area of impact explored in the study (please cross out or delete as appropriate)	71. Mental Health;<br>72. Impact on partner relationships, sexual and reproductive behaviour;
Evidence of impact to the general population from lockdown measures (e.g. 10% decrease of disposable income before and after the peak of the epidemic; 30% reduction	More than half of the survey participants (57.3%) reported extremely severe distress symptoms, as per the DASS-21; Violence against women also reportedly increased significantly during the lockdown (from 4.4 to 14.8%; $p < 0.001$). Psychological abuse was the most frequent type of violence (96%). Women who had experienced abuse before the lockdown

of hospital visits for maternal services)	were at an increased risk of violence during lockdown ($p < 0.001$; OR = 19.34 [8.71– 43.00])
Specific evidence of impact on children (e.g. 30% increase of weight during two weeks of implementation of stay at home policy; or: ‘calculated 365,000 extra malaria deaths calculated as a consequence of disruption of bed nets distribution)	N/A
Specific evidence of impact on women (e.g. 20% reduction of female employment in the fishing sector, as a result of decreased demand for fish during the epidemic)	The Depression Anxiety and Stress Scales (DASS-21) was used to assess depression and anxiety; The history of domestic violence and its types before and during the lockdown were assessed by yes or no questions. Anxiety, depression symptoms, and stress were found in about 85% of the women. More than half of participants had symptoms of extreme severity for the three axes of the DASS-21 scale. They found that violence against women increased during lockdown from 4.4 to 14.8% ($p < 0.001$). Psychological violence seemed to be the most frequent type. All those who were abused (96%) experienced psychological (emotional) violence, followed by economic (41%) and then physical violence (10%)
Relevance and importance of the paper for our own systematic review	☐ Key paper for the review
Other comments or issues to be noted about this paper	One of the few papers on domestic violence and its links to depression on women

Table S1. Data extraction form for SLR on the health impact of lockdown measures for women and children in LMICs

Title of the paper:	A cross-sectional study of psychological wellbeing of Indian adults during the Covid-19 lockdown: Different strokes for different folks
Author(s) and date:	Anupam Joya Sharma and Malavika A. Subramanyam, 2020
Quality appraisal according to the relevant Johanna Briggs Institute tool (please cross out or delete accordingly)	☐ Include
Epidemic of reference:	Covid-19
Research methods employed (e.g. qualitative interviews; modelling; surveys; systematic review)	Mixed methods, but primarily a cross sectional survey (n=282) ,informed by 14 qualitative interviews
Specific lockdown measure investigated in the study (e.g. generic quarantine or unspecified lockdown measure; curfew; stay at	‘lockdown’ in India which the authors noted ‘restricted citizens’ physical mobility, advocated social distancing norms, and limited a majority of public services while allowing the essential ones’

home policy; travel restrictions; school closures; market and trade shutdowns; social distancing)	
Broad area of impact explored in the study (please cross out or delete as appropriate)	73. Mental Health;<br>74. Dietary patterns and nutrition;<br>75. Impact on partner relationships, sexual and reproductive behaviour;<br>76. Other (please specify): social relationships
Evidence of impact to the general population from lockdown measures (e.g. 10% decrease of disposable income before and after the peak of the epidemic; 30% reduction of hospital visits for maternal services)	Gender Anxiety Disorder Scale scores higher for:<br>LBGT adults v heterosexual adults (β = 2.44)<br>High risk groups v low risk groups (β = 2.20)<br>History of depression v no history of depression (β = 3.89)<br><br>Those with a history of depression reported higher levels of depressive symptoms (β = 4.34),<br><br>Those with a history of depression reported higher levels of internet addiction symptoms (β = 4.55)<br><br>Higher levels of pornography usage in:<br>LBGT adults v heterosexual adults (β = 2.72)<br>High risk groups v low risk groups (β = 2.80)<br>Same-sex partnerships v opposite sex partnerships (β = 9.15)<br>History of depression v no history of depression (β = 2.63)<br><br>Higher levels of masturbation<br>LBGT adults and those in same-sex partnerships v heterosexual adults (β = 2.07)<br><br>Those with higher depressive symptoms also reported higher levels of sleep disorders and changes in food consumption
Specific evidence of impact on children (e.g. 30% increase of weight during two weeks of implementation of stay at home policy; or: 'calculated 365,000 extra malaria deaths calculated as a consequence of disruption of bed nets distribution)	No specific evidence of the impact on children was reported
Specific evidence of impact on women (e.g. 20% reduction of female employment in the fishing sector, as a result of decreased demand for fish during the epidemic)	No specific evidence on the impact on women was reported.<br>Women made up 36.4% of the sample

Relevance and importance of the paper for our own systematic review	☐ Paper of some importance for some sections of the review
Other comments or issues to be noted about this paper	This was a very small sample size (n=282). The sample was very skewed towards those living in urban areas (81% from urban areas) and well educated (for example 57.5% had a postgraduate qualification!), so it is unclear how representative this was of the general population.<br>The sampling method was snowballing through personal and professional networks.

Table S1. Data extraction form for SLR on the health impact of lockdown measures for women and children in LMICs

Title of the paper:	Community-based health care is an essential component of a resilient health system: evidence from Ebola outbreak in Liberia
Author(s) and date:	Kendra Siekmans, Salim Sohani, Tamba Boima, Florence Koffa, Luay Basil and Saïd Laaziz
Quality appraisal according to the relevant Johanna Briggs Institute tool (please cross out or delete accordingly)	☐ Include
Epidemic of reference:	Ebola
Research methods employed (e.g. qualitative interviews; modelling; surveys; systematic review)	Mixed methods:<br>descriptive observational study design used mixed methods to collect data from CHWs (structured survey, n = 60; focus group discussions, n = 16), government health facility workers and project staff.<br><br>Monthly data on child diarrhea and pneumonia treatment were gathered from CHW case registers and local health facility records.
Specific lockdown measure investigated in the study (e.g. generic quarantine or unspecified lockdown measure; curfew; stay at home policy; travel restrictions; school closures; market and trade shutdowns; social distancing)	Study assesses<br>"the value of a community-based health system in ensuring continued delivery of essential health services in the context of a national crisis (Ebola epidemic) in three Liberian counties."<br><br>So a bit related to lockdown, assuming that Ebola lead to the closure of the health system, but that this community based health system was able to continue
Broad area of impact explored in the study (please cross out or delete as appropriate)	77. Disruption of routine services for mother and child services;<br>78. Demand for healthcare / Healthcare seeking behaviour;<br>79.
Evidence of impact to the general population from lockdown measures (e.g. 10% decrease of disposable income before and after the peak of the epidemic; 30% reduction	"medicine stocks were available during the outbreak but CHWs reported inability to access them due to travel restrictions and facility closures"

of hospital visits for maternal services)	
Specific evidence of impact on children (e.g. 30% increase of weight during two weeks of implementation of stay at home policy; or: 'calculated 365,000 extra malaria deaths calculated as a consequence of disruption of bed nets distribution)	
Specific evidence of impact on women (e.g. 20% reduction of female employment in the fishing sector, as a result of decreased demand for fish during the epidemic)	
Relevance and importance of the paper for our own systematic review	☐ Secondary paper
Other comments or issues to be noted about this paper	Maybe exclude? The paper talks about Ebola in general but not much is directly related to the impact of control measures

Table 16: Data extraction form for SLR on the health impact of lockdown measures for women and children in LMICs

Title of the paper:	Reduced vaccination and the risk of measles and other childhood infections post-Ebola
Author(s) and date:	Takahashi et al (2015)
Quality appraisal according to the relevant Johanna Briggs Institute tool (please cross out or delete accordingly)	☐ Include
Epidemic of reference:	Ebola
Research methods employed (e.g. qualitative interviews; modelling; surveys; systematic review)	Epidemiological and geospatial modelling to predict the number of deaths from unvaccinated areas
Specific lockdown measure investigated in the study (e.g. generic quarantine or unspecified lockdown measure; curfew; stay at home policy; travel restrictions; school closures; market and trade shutdowns; social distancing)	Disruption of measles immunization services in Guinea, Liberia and Sierra Leone during the first 18 months of the Ebola epidemic
Broad area of impact explored in the study (please cross out or delete as appropriate)	80. Disruption of routine services for mother and child services;

Evidence of impact to the general population from lockdown measures (e.g. 10% decrease of disposable income before and after the peak of the epidemic; 30% reduction of hospital visits for maternal services)	Assuming a 75% reduction of vaccination rates, the study projects that after 6 to 18 months of disruptions, a large connected cluster of children unvaccinated for measles will accumulate across Guinea, Liberia, and Sierra Leone. This pool of susceptibility increases the expected size of a regional measles outbreak from 127,000 to 227,000 cases after 18 months, resulting in 2000 to 16,000 additional deaths from multiple infectious diseases in the community.
Specific evidence of impact on children (e.g. 30% increase of weight during two weeks of implementation of stay at home policy; or: 'calculated 365,000 extra malaria deaths calculated as a consequence of disruption of bed nets distribution)	With every month of health care disruptions, the study estimated that the number of children between 9 months and 5 years of age who are not vaccinated against measles increases by an average of 19,514, reaching 1,129,376 after 18 months. In the likely case of outbreaks, this susceptibility could generate up to 5,209 additional deaths from measles only.
Specific evidence of impact on women (e.g. 20% reduction of female employment in the fishing sector, as a result of decreased demand for fish during the epidemic)	N/A
Relevance and importance of the paper for our own systematic review	☐ Key paper for the review
Other comments or issues to be noted about this paper	Extremely relevant paper from a reputable journal (Science) from a past epidemic.

Table 17: Data extraction form for SLR on the health impact of lockdown measures for women and children in LMICs

Title of the paper:	Mental health and its correlates among children and adolescents during COVID-19 school closure: The importance of parent-child discussion
Author(s) and date:	Tang et al (2020)
Quality appraisal according to the relevant Johanna Briggs Institute tool (please cross out or delete accordingly)	☐ Exclude
Epidemic of reference:	
Research methods employed (e.g. qualitative interviews; modelling; surveys; systematic review)	
Specific lockdown measure investigated in the study (e.g. generic quarantine or unspecified lockdown measure; curfew; stay at home policy; travel	

restrictions; school closures; market and trade shutdowns; social distancing)	
Broad area of impact explored in the study (please cross out or delete as appropriate)	81. Mental Health;<br>82. Dietary patterns and nutrition;<br>83. Physical exercise and impacts on obesity and specific diseases such as diabetes;<br>84. Disruption of routine services for mother and child services;<br>85. Demand for healthcare / Healthcare seeking behaviour;<br>86. Impact on partner relationships, sexual and reproductive behaviour;<br>87. Increases in social vulnerability, poverty and impact on human development;<br>88. Other (please specify).
Evidence of impact to the general population from lockdown measures (e.g. 10% decrease of disposable income before and after the peak of the epidemic; 30% reduction of hospital visits for maternal services)	
Specific evidence of impact on children (e.g. 30% increase of weight during two weeks of implementation of stay at home policy; or: 'calculated 365,000 extra malaria deaths calculated as a consequence of disruption of bed nets distribution)	
Specific evidence of impact on women (e.g. 20% reduction of female employment in the fishing sector, as a result of decreased demand for fish during the epidemic)	
Relevance and importance of the paper for our own systematic review	☐ Key paper for the review<br>☐ Paper of some importance for some sections of the review<br>☐ Secondary paper
Other comments or issues to be noted about this paper	

Table 18: Data extraction form for SLR on the health impact of lockdown measures for women and children in LMICs

Title of the paper:	The 2014-2015 Ebola virus disease outbreak and primary healthcare delivery in Liberia: Time-series analyses for 2010-2016
Author(s) and date:	Wagenaar et al (2018)

Quality appraisal according to the relevant Johanna Briggs Institute tool (please cross out or delete accordingly)	☐ Include
Epidemic of reference:	Ebola
Research methods employed (e.g. qualitative interviews; modelling; surveys; systematic review)	Time-series analysis
Specific lockdown measure investigated in the study (e.g. generic quarantine or unspecified lockdown measure; curfew; stay at home policy; travel restrictions; school closures; market and trade shutdowns; social distancing)	Suspension of healthcare services during the epidemic; stay at home
Broad area of impact explored in the study (please cross out or delete as appropriate)	89. Disruption of routine services for mother and child services;<br>90. Demand for healthcare / Healthcare seeking behaviour;
Evidence of impact to the general population from lockdown measures (e.g. 10% decrease of disposable income before and after the peak of the epidemic; 30% reduction of hospital visits for maternal services)	The authors found that it took only 4 months during the ebola epidemic to lose between 35% and 67% of essential primary care health system outputs across Liberian clinics, and that 19 months post-EVD, all health system indicators had recovered to their pre-EVD levels. They estimated a loss of an estimated 776,110 clinic visits; 101,857 artemisinin-based combination therapy treatments for malaria, and 45,024 treatments of acute respiratory infections due to the EVD outbreak will continue to severely affect population health.
Specific evidence of impact on children (e.g. 30% increase of weight during two weeks of implementation of stay at home policy; or: 'calculated 365,000 extra malaria deaths calculated as a consequence of disruption of bed nets distribution)	They estimated a loss of 24,449 bacille Calmette-GueÂrin vaccinations, 9,129 measles vaccinations, 12,941 first pentavalent vaccinations, 5,122 institutional births, 17,191 postnatal care visits within 6 weeks of birth.
Specific evidence of impact on women (e.g. 20% reduction of female employment in the fishing sector, as a result of decreased demand for fish during the epidemic)	N/A
Relevance and importance of the paper for our own systematic review	☐ Key paper for the review

Other comments or issues to be noted about this paper	Great to show the loss of health services for children during 6 months of the Ebola epidemic in liberia, and that it took almost two years for the system to recover.

Table 19: Data extraction form for SLR on the health impact of lockdown measures for women and children in LMICs

Title of the paper:	Indirect effects of the COVID-19 pandemic on malaria intervention coverage, morbidity, and mortality in Africa: a geospatial modelling analysis
Author(s) and date:	Weiss et al (2020)
Quality appraisal according to the relevant Johanna Briggs Institute tool (please cross out or delete accordingly)	☐ Include
Epidemic of reference:	COVID-19
Research methods employed (e.g. qualitative interviews; modelling; surveys; systematic review)	Spatiotemporal Bayesian geostatistical models to generate geospatial estimates of the clinical case incidence and mortality of malaria
Specific lockdown measure investigated in the study (e.g. generic quarantine or unspecified lockdown measure; curfew; stay at home policy; travel restrictions; school closures; market and trade shutdowns; social distancing)	• Impact of travel restrictions on availability of health workers;<br>• Trade restrictions on disruption of supply chains of antimalarials and distribution of ITNs;<br>• Stay at home policies on treatment seeking behaviour for antimalarials.
Broad area of impact explored in the study (please cross out or delete as appropriate)	91. Disruption of routine services for mother and child services;<br>92. Demand for healthcare / Healthcare seeking behaviour;
Evidence of impact to the general population from lockdown measures (e.g. 10% decrease of disposable income before and after the peak of the epidemic; 30% reduction of hospital visits for maternal services)	Under 9 different scenarios of disruption of services, there could be additional 215-262 million cases of malaria worldwide, and between 101-382 thousand extra malaria deaths.
Specific evidence of impact on children (e.g. 30% increase of weight during two weeks of implementation of stay at home policy; or: 'calculated 365,000 extra malaria deaths calculated as a consequence of disruption of bed nets distribution)	Malaria control relies heavily on the decision making of patients and their families, including choosing to leave their homes to seek care for febrile children and receiving ITNs delivered at antenatal clinics or schools. A substantial proportion of the additional cases and deaths would be from children <5 years.
Specific evidence of impact on women (e.g. 20% reduction of	N/A

female employment in the fishing sector, as a result of decreased demand for fish during the epidemic)	
Relevance and importance of the paper for our own systematic review	☐ Paper of some importance for some sections of the review
Other comments or issues to be noted about this paper	

Table S1. Data extraction form for SLR on the health impact of lockdown measures for women and children in LMICs

Title of the paper:	Association between Physical Activity and Mood States of Children and Adolescents in Social Isolation during the COVID-19 Epidemic
Author(s) and date:	Xinxin Zhang , Wenfei Zhu , Sifan Kang, Longkun Qiu, Zijun Lu and Yuliang Sun
Quality appraisal according to the relevant Johanna Briggs Institute tool (please cross out or delete accordingly)	☐ Include
Epidemic of reference:	Covid-19
Research methods employed (e.g. qualitative interviews; modelling; surveys; systematic review)	Survey. N=9979 students at primary schools years 4-6. Average age 11.63 + or – 1.23 years
Specific lockdown measure investigated in the study (e.g. generic quarantine or unspecified lockdown measure; curfew; stay at home policy; travel restrictions; school closures; market and trade shutdowns; social distancing)	Impact of social isolation. Article mentions:<br><br>"partial blockade" and "social isolation" strategies by closing schools, factories and other public places. People are required to stay at home."<br><br>Conducted online<br>Hosted on an online data collection site in China
Broad area of impact explored in the study (please cross out or delete as appropriate)	93. Mental Health;<br>94. Physical exercise and impacts on obesity and specific diseases such as diabetes;<br>95.
Evidence of impact to the general population from lockdown measures (e.g. 10% decrease of disposable income before and after the peak of the epidemic; 30% reduction of hospital visits for maternal services)	
Specific evidence of impact on children (e.g. 30% increase of weight during two weeks of	51% boys 49% girls

implementation of stay at home policy; or: 'calculated 365,000 extra malaria deaths calculated as a consequence of disruption of bed nets distribution)	Reduced physical activity – 23.19 minutes per day in comparison to other studies in non-epidemic times in China that had reported 35 minutes and 41.1 minutes per day<br><br>This was primarily due to the schools shutting and the need to isolate.<br><br>The study (actually the main focus of it) found a significant relationship between reduced physical activity and mood states. Children with higher physical activity in this sample had lower levels of negative moods (In the negative mood subscale, the total scores of Depression, Confusion, Anger, and Fatigue)<br><br>Boys had higher rates of negative moods than girls<br><br>Boys had less MVPA and moderate PA than girls.
Specific evidence of impact on women (e.g. 20% reduction of female employment in the fishing sector, as a result of decreased demand for fish during the epidemic)	
Relevance and importance of the paper for our own systematic review	☐ Paper of some importance for some sections of the review
Other comments or issues to be noted about this paper	A large sample. However, lockdown was not necessarily the focus here -

Table 20: Data extraction form for SLR on the health impact of lockdown measures for women and children in LMICs

Title of the paper:	Routine childhood immunisation during the COVID-19 pandemic in Africa: a benefit-risk analysis of health benefits versus excess risk of SARS-CoV-2 infection
Author(s) and date:	Abbas et al (2020)
Quality appraisal according to the relevant Johanna Briggs Institute tool (please cross out or delete accordingly)	☐ Include
Epidemic of reference:	COVID-19
Research methods employed (e.g. qualitative interviews; modelling; surveys; systematic review)	Modelling risk-benefit analysis
Specific lockdown measure investigated in the study (e.g. generic quarantine or	Disruption of all immunization campaigns and services

unspecified lockdown measure; curfew; stay at home policy; travel restrictions; school closures; market and trade shutdowns; social distancing)	
Broad area of impact explored in the study (please cross out or delete as appropriate)	96. Disruption of routine services for mother and child services;
Evidence of impact to the general population from lockdown measures (e.g. 10% decrease of disposable income before and after the peak of the epidemic; 30% reduction of hospital visits for maternal services)	The benefit of routine childhood immunisation programmes in all 54 African countries was found to be greater than the COVID-19 risk associated with these vaccination clinic visits.
Specific evidence of impact on children (e.g. 30% increase of weight during two weeks of implementation of stay at home policy; or: 'calculated 365,000 extra malaria deaths calculated as a consequence of disruption of bed nets distribution)	For every one excess COVID-19 death attributable to SARS-CoV-2 infections acquired during routine vaccination clinic visits, 84 deaths in children up to 5 years of age could be prevented by sustaining routine childhood immunisation in Africa.
Specific evidence of impact on women (e.g. 20% reduction of female employment in the fishing sector, as a result of decreased demand for fish during the epidemic)	
Relevance and importance of the paper for our own systematic review	☐ Key paper for the review
Other comments or issues to be noted about this paper	Very important argument that sustaining the immunization programmes in Africa would vastly offsets the COVID-19 deaths under all scenarios.
